# Genome sequencing and analysis of the first spontaneous Nanosilver resistant bacterium *Proteus mirabilis* strain SCDR1

**DOI:** 10.1186/s13756-017-0277-x

**Published:** 2017-11-23

**Authors:** Amr T. M. Saeb, Khalid A. Al-Rubeaan, Mohamed Abouelhoda, Manojkumar Selvaraju, Hamsa T. Tayeb

**Affiliations:** 1 Genetics and Biotechnology Department, Strategic Center for Diabetes Research, College of medicine, King Saud University, Riyadh, Kingdom of Saudi Arabia; 2 Genetics Department, King Faisal Specialist Hospital and Research Center, Riyadh, Kingdom of Saudi Arabia; 3 Saudi Human Genome Project, King Abdulaziz City for Science and Technology (KACST), Riyadh, Kingdom of Saudi Arabia; 4 Integrated Gulf Biosystems, Riyadh, Kingdom of Saudi Arabia

**Keywords:** *Proteus Mirabilis*, Multi-drug resistance, Silver nanoparticles, Genome analysis, Pathogenomics, Biofilm formation, Swarming mobility, Resistome, Glutathione S-transferase, Copper/silver efflux system

## Abstract

**Background:**

*P. mirabilis* is a common uropathogenic bacterium that can cause major complications in patients with long-standing indwelling catheters or patients with urinary tract anomalies. In addition, *P. mirabilis* is a common cause of chronic osteomyelitis in Diabetic foot ulcer (DFU) patients. We isolated *P. mirabilis SCDR1* from a Diabetic ulcer patient. We examined *P. mirabilis SCDR1* levels of resistance against Nanosilver colloids, the commercial Nanosilver and silver containing bandages and commonly used antibiotics. We utilized next generation sequencing techniques (NGS), bioinformatics, phylogenetic analysis and pathogenomics in the characterization of the infectious pathogen.

**Results:**

*P. mirabilis SCDR1* was the first Nanosilver resistant isolate collected from a diabetic patient polyclonal infection. *P. mirabilis SCDR1* showed high levels of resistance against Nanosilver colloids, Nanosilver chitosan composite and the commercially available Nanosilver and silver bandages. The *P. mirabilis* -SCDR1 genome size is 3,815,621 bp. with G + C content of 38.44%. *P. mirabilis*-SCDR1 genome contains a total of 3533 genes, 3414 coding DNA sequence genes, 11, 10, 18 rRNAs (5S, 16S, and 23S), and 76 tRNAs. Our isolate contains all the required pathogenicity and virulence factors to establish a successful infection. *P. mirabilis* SCDR1 isolate is a potential virulent pathogen that despite its original isolation site, the wound, can establish kidney infection and its associated complications. *P. mirabilis SCDR1* contains several mechanisms for antibiotics and metals resistance, including, biofilm formation, swarming mobility, efflux systems, and enzymatic detoxification.

**Conclusion:**

*P. mirabilis SCDR1* is the first reported spontaneous Nanosilver resistant bacterial strain. *P. mirabilis SCDR1* possesses several mechanisms that may lead to the observed Nanosilver resistance.

**Electronic supplementary material:**

The online version of this article (10.1186/s13756-017-0277-x) contains supplementary material, which is available to authorized users.

## Background

The production and utilization of nanosilver is one of the primary and still growing applications in the field of nanotechnology. Nanosilver is used as the essential antimicrobial ingredient in both clinical and environmental technologies. Nanosilver is utilized in the formulation of dental resin amalgams, medical device coatings, water filter antimicrobial coating, antimicrobial agents in air sanitizers, textiles, pillows, respirators, socks, wet wipes, detergents, soaps, shampoos, toothpastes, washing machines, bone cement, wound dressings, hospital beds and furniture to control infection and support anti-biofilm activity [1–8]. Nanosilver is known to exert inhibitory and bactericidal effects against many Gram-positive, Gram-negative and fungal pathogens [9]. Latest studies suggest that the use of nanosilver-containing wound dressings prevents or reduces microbial growth in wounds, and may improve the healing process [10]. Moreover, antibacterial nanosilver-containing wound dressing gels may be important for patients that are at risk of non-healing of diabetic foot wounds and traumatic/surgical wounds [11]. Increased usage of nanosilver in both medical and environmental products has generated concerns about the development of bacterial resistance against the antimicrobial ingredient. Bacterial resistance against metallic silver has been documented with several bacterial strains such as *E. coli Enterobacter cloacae*, *Klebsiella pneumoniae* and *Salmonella typhimurium* [12, 13]. However, information about bacterial resistance against Nanosilver is scarce. Only Gunawan et al., (2013) reported the occurrence of induced adaptation, of non-targeted environmental *Bacillus* species, to antimicrobial Nanosilver [14]. In this study, we report on a spontaneous nanosilver-resistant *Proteus mirabilis* isolate (“SCDR1”). *Proteus mirabilis* is a motile gram-negative bacterium that is characterized by its swarming behavior [15, 16]. *P. mirabilis* is a common uropathogen that can cause major complications. In addition, *P. mirabilis* can cause respiratory and wound infections, bacteremia, and other infections [16–21]. In fact, *P. mirabilis* is a common cause of chronic osteomyelitis in Diabetic foot ulcer (DFU) patients along with *Bacteroides fragilis, E coli,* and *Klebsiella pneumoniae* [22]*.* Generally, *P. mirabilis* is responsible for 90% of genus *Proteus* infections, and can be considered as a community-acquired infection [23]. As a pathogen *P. mirabilis* acquires many virulence determinants that enable it to establish successful infections [24–26]*.* A lot of information concerning antibiotic resistance is available for *P. mirabilis* [27–35]. *P. mirabilis* is intrinsically resistant to tetracyclines and polymyxins. Moreover, multidrug-resistant (MDR) *P. mirabilis* strain resistance to β-lactams, aminoglycosides, fluoroquinolones, phenicols, streptothricin, tetracycline, and trimethoprim-sulfamethoxazole has been reported [36]. However, limited information about heavy metals, including silver, is available. In this study, we present the first report and genome sequence of the nanosilver resistant bacterium *P. mirabilis* strain SCDR1, isolated from diabetic foot ulcer (DFU) patient.

## Methods

### Bacterial isolate

*Proteus mirabilis* strain SCDR1 was isolated from a diabetic ulcer patient in the diabetic foot unit at the University Diabetes Center, King Saud University. *P. mirabilis SCDR1* was the first nanosilver resistant isolate to be collected from a diabetic patient’s polyclonal infection. A Proper wound swab was obtained from the patient and was sent for further microbiological study and culture. Wounds needing debridement were debrided before swabbing the surface of the wound. The specimen was inoculated onto blood agar (BA; Oxoid, Basingstoke, UK) and MacConkey agar (Oxoid) and incubated at 37 °C for 24–48 h. The Vitek 2 system and its advanced expert system were used for microbial identification, antibiotic sensitivity testing, and the interpretation of results. ID GN cards were used to identify the bacterial isolate, and AST-N204 was used for the antimicrobial susceptibility testing of gram-negative rods. Manual disk diffusion and MIC method for AgNPs and antibiotic sensitivity testing were performed when required. Results were categorized according to EUCAST 2.0 VITEK 2 MIC breakpoints.

### Preparation of colloidal and composite Nanosilver and commercial products for antimicrobial activity testing

Colloidal silver nanoparticles were prepared and characterized, and their concentration was determined as described by Saeb et al., 2014 [9]. Nanosilver chitosan composite preparations were made by chemical reduction method, as described by Latif et al., 2015 [37]. Moreover, the following commercially silver and nanosilver containing wound dressing bandages were used for antimicrobial activity testing: Silvercel non-adherent antimicrobial alginate Dressing (Acelity L.P. Inc., San Antonio, Texas, USA), Sorbsan Silver dressing made of Calcium alginate with silver (Aspen Medical Europe Ltd., Leicestershire, UK), ColActive® Plus Ag (Covalon Technologies Ltd., Mississauga, Ontario, Canada), exsalt®SD7 wound dressing (Exciton Technologies, Edmonton, Alberta, Canada), Puracol Plus AG+ Collagen Dressings with Silver (Medline, Mundelein, Illinois, USA) and ACTISORB™ silver antimicrobial wound dressing 220 (Acelity L.P. Inc., San Antonio, Texas, USA).

### Antimicrobial susceptibility test

Antimicrobial activities were performed against the following strains: *Pseudomonas aeruginosa* ATCC 27853, *Staphylococcus aureus* ATCC 29213, *Proteus mirabilis* ATCC 29906, *Klebsiella pneumoniae* ATCC 700603, *E. coli* ATCC 25922 and *Enterobacter cloacae* ATCC 29212.

### Disk diffusion antimicrobial susceptibility testing

Disk diffusion antimicrobial susceptibility testing was performed as described by Matuschek et al. [38]. Briefly, Mueller–Hinton (MH) agar plates were inoculated with agar with an inoculum corresponding to a McFarland 0.5 turbidity with a sterile cotton swab to prepare bacterial lawns of the abovementioned bacterial test strains. Sterile discs were loaded with different concentrations (50–200 ppm) of colloidal silver nanoparticles solutions and the Nanosilver chitosan composite (composite concentration ranged from 0.1% and 0.01 M to 3.2% and 0.16 M from chitosan and Silver nitrate respectively) and then placed on Mueller–Hinton (MH) agar plates with bacterial lawns. Within 15 min of application of antimicrobial disks, the plates were inverted and incubated at 37^∘^C for 16 h. All experiments were done in aseptic conditions in a laminar air flow cabinet. After incubation, inhibition zones were read at the point where no apparent growth was detected. The inhibition zone diameters were measured to the nearest millimeter. Similarly, 5 mm desks from the commercially available bandages were prepared in aseptic conditions and tested for antimicrobial activity, as previously described.

### Minimum bactericidal (MBC) and minimal inhibitory concertation (MIC) test

MBC and MIC testing were performed as described by Holla et al., [39]. Briefly, a dilution with 1 × 10^5^ CFU/ml (equivalent to 0.5 McFarland) was used as an inoculum for MIC testing. Different volumes that contained a range of silver Nanoparticles (50–700 ppm) were delivered to 7.5 ml of Muller-Hinton (MH) broth, each inoculated with 0.2 ml of the bacterial suspensions. Within 15 min of application of silver nanoparticles, the tubes were incubated at 37^∘^C for 16 h in a shaker incubator at 200 rpm. We included a positive control (tubes containing inoculum and nutrient media without silver nanoparticles) and a negative control (tubes containing silver nanoparticles and nutrient media without inoculum).

### Biofilm formation

In order to test the ability of *P. mirabilis* SCDR1 isolate to form biofilm, a culture was prepared by inoculation on Columbia agar, supplemented with 5% blood and incubated at 37 C° for 24 h. The culture was then used to prepare 0.5 McFarland standard bacterial suspension. Wells of sterile 96- well flat- bottomed plastic microplates were filled with 250 μL of the Brain-heart infusion broth. Negative control wells contained the broth only. Twenty μL of bacterial suspension were then added to each well. The plate was incubated at 37 C° for 24 h. Following the incubation, the content of each well was aspirated and washed three times with 300 μL of sterile distilled water. The remaining attached bacteria were fixed with 200 μL of methanol per well, and after 15 min the plates were emptied and left to dry air. After this, the plates were stained for 5 min with 160 μL per well of crystal violet used for gram stain. Excess stain was rinsed off by placing the microplates under running tap water. After the plates were air dried, the dye which was bound to the adherent cells was re-solubilized with 160 μL of 33% (*v*/v) glacial acetic acid per well. The optical density (OD) was measured at 570 nm [40].

### Molecular genomics analysis

#### DNA purification, sequencing, bioinformatics and phylogenetic analysis

DNA isolation, purification, genome sequencing, bioinformatics and phylogenetic analysis were performed as described by Saeb et al., 2017 [41]. In addition, we used Mauve [42] and CoCoNUT [43] to generate the whole genome pairwise and multiple alignments of the draft *P. mirabilis* strain SCDR1 genome against selected reference genomes. Furthermore, we performed whole genome phylogeny based proteomic comparison among *P. mirabilis* SCDR1 isolate and other related *Proteus mirabilis* strains using Proteome Comparison service which is a protein sequence-based comparison using bi-directional BLASTP available at (https://www.patricbrc.org/app/SeqComparison) [44].

#### Gene annotation and Pathogenomics analysis

*P. mirabilis* SCDR1 genome contigs were annotated using the Prokaryotic Genomes Automatic Annotation Pipeline (**PGAAP**) available at NCBI (http://www.ncbi.nlm.nih.gov/). In addition, contigs were further annotated using the bacterial bioinformatics database and analysis resource (**PATRIC**) gene annotation service (https://www.patricbrc.org/app/Annotation) [44]. The **PathogenFinder 1.1** pathogenicity prediction program available at (https://cge.cbs.dtu.dk/services/PathogenFinder/) was used to examine the nature of *P. mirabilis* SCDR1 as a human pathogen [45]. Virulence gene sequences and functions, corresponding to different major bacterial virulence factors of *Proteus mirabilis* were collected from GenBank and validated using virulence factors of the pathogenic bacteria database available at (http://www.mgc.ac.cn/VFs/) [46], the Victors virulence factors search program available at (http://www.phidias.us/victors/) and the PATRIC_VF tool available at https://www.patricbrc.org/= [44].

#### Resistome analysis

*P. mirabilis* SCDR1 genome contigs were investigated manually for the presence of antibiotic resistance loci using the **PGAAP** and **PATRIC** gene annotation services. Antibiotic resistance loci were further investigated using specialized search tools and services, namely **Antibiotic Resistance Gene Search** available at (https://www.patricbrc.org/=) [44], **Genome Feature Finder** (antibiotic resistance) available at (https://www.patricbrc.org/=) [44], **ARDB** (Antibiotic Resistance Genes Database) available at (https://ardb.cbcb.umd.edu/) [47],

**CARD** (The Comprehensive Antibiotic Resistance Database) available at (https://card.mcmaster.ca/) [48, 49], **Specialty Gene Search** available at (https://www.patricbrc.org/=) and **ResFinder 2.1** available at (https://cge.cbs.dtu.dk//services/ResFinder/) [50].

The heavy metal resistance gene search for *P. mirabilis* SCDR1 contigs were investigated using **PGAAP** and **PATRIC** gene annotation services, **PATRIC Feature Finder** searches tool and **BacMet** (antibacterial biocide and metal resistance genes database) available at (http://bacmet.biomedicine.gu.se/) [44, 51].

## Results

### Initial identification and antimicrobial susceptibility test

The Vitek 2 system showed that our isolate belongs to the *Proteus mirabilis* species. Antibiotic sensitivity testing using Vitek 2 AST-N204 card showed that our isolate *P. mirabilis* SCDR1 is resistant to ampicillin, nitrofurantoin, and Trimethoprim/ Sulfamethoxazole. In addition, *P. mirabilis* SCDR1 was resistant to ethidium bromide, tetracycline, tigecycline, colistin, polymyxin B, rifamycin, doxycycline, vancomycin, fusidic acid, bacitracin, metronidazole, clarithromycin, erythromycin, oxacillin, clindamycin, trimethoprim, novobiocin, and minocycline. *P. mirabilis* SCDR1 was intermediate resistant against nalidixic acid, Imipenem, and Cefuroxime. Conversely, it was sensitive to chloramphenicol, amoxicillin/ clavulanic Acid, piperacillin/tazobactam, cefotaxime, ceftazidime, cefepime, cefaclor, cephalothin, ertapenem, meropenem, amikacin, gentamicin, ciprofloxacin, norfloxacin, tobramycin, streptomycin, and fosfomycin.

*P. mirabilis* SCDR1 isolate showed high resistance against colloidal and composite Nanosilver and metallic silver compared with other tested Gram positive and negative bacterial species. For instance, Table 1, shows the resistance of *P. mirabilis SCDR1* against colloidal Nanosilver assessed by the disk diffusion method, in comparison with *S. aureus* ATCC 29213, *P. aeruginosa* ATCC 27853, *E. coli* ATCC 25922 and *E. cloacae* ATCC 29212. Generally, *P. mirabilis SCDR1* showed high resistance (0.0 cm), while *K. pneumoniae* showed the highest sensitivity (1.5–1.9 cm) against all tested silver nanoparticle concentrations (50–200 ppm). *S. aureus* also showed high sensitivity (1.4–1.6 cm) against all tested silver nanoparticle concentrations. None of the tested bacterial isolates except for *P. mirabilis SCDR1* showed any resistance against silver-nanoparticles, even against the lowest concentration (50 ppm). Furthermore, Table 2 shows the resistance of *P. mirabilis* SCDR1 against colloidal Nanosilver assessed by a minimal inhibitory concentration method, compared with other tested Gram positive and negative bacterial species. Once more, *P. mirabilis SCDR1* showed high resistance against the gradually increased concentrations of colloidal nanosilver. We observed *P. mirabilis SCDR1* bacterial growth to colloidal Nanosilver concentration up to 500 ppm. On the other hand, *K. pneumoniae* showed the highest sensitivity against silver nanoparticles, with no observed growth at only 100 ppm colloidal nanosilver concentration. In addition, both *E. coli* and *P. aeruginosa* showed high sensitivity against silver nanoparticles, with no observed growth at 150 ppm colloidal Nanosilver concentration. Conversely, *S. aureus* tolerated only 200 ppm colloidal Nanosilver concentration. Similarly, Table 3 shows the resistance of *P. mirabilis* SCDR1 against silver and Nanosilver composite assessed by disk diffusion method. Nanosilver chitosan composites, with a concentration ranging from between 0.1% and 0.01 M to 3.2% and 0.16 M from chitosan and Silver nitrate respectively, had a comparable killing effect on both Gram positive and negative bacterial, namely *S. aureus* and *P. aeruginosa.* Meanwhile, none of the tested Nanosilver chitosan composites had any killing effect on *P. mirabilis* SCDR1. Similarly, all the tested commercially available silver and Nanosilver containing wound dressing bandages showed the enhanced killing effect on both *S. aureus* and *P. aeruginosa.* However, all these wound dressing bandages failed to inhibit *P. mirabilis* SCDR1 growth. *P. mirabilis* SCDR1 was able to produce strong biofilm with OD of 0.296.

**Table 1:** Resistance of *P. mirabilis* SCDR1 against colloidal Nano-Silver assessed by desk diffusion method

S. No.	Sample ID	Zone Of Inhibition (cm) *S. aureus*	Zone Of Inhibition (cm) *E. cloacae*	Zone Of Inhibition (cm) *P. aeruginosa*	Zone Of Inhibition (cm) *E. coli*	Zone Of Inhibition (cm) *K. pneumoniae*	Zone Of Inhibition (cm) *P. mirabilis* SCDR1
1	200 ppm	1.6 cm	1.5 cm	1.4 cm	1.1 cm	1.9 cm	0.0 cm
2	150 ppm	1.5 cm	1.2 cm	1.3 cm	1.0 cm	1.7 cm	0.0 cm
3	100 ppm	1.5 cm	1.2 cm	1.3 cm	1.0 cm	1.6 cm	0.0 cm
4	50 ppm	1.4 cm	1.1 cm	1.1 cm	0.9 cm	1.5 cm	0.0 cm

**Table 2:** Resistance of *P. mirabilis* SCDR1 against colloidal Nanosilver assessed by minimal inhibitory concentration method

AgNPs (concentration in ppm)			Bacterial species/strain				
	*S. aureus* ATCC 29213	*P. aeruginosa* ATCC 27853	*E. cloacae* ATCC 29212	*E. coli* ATCC 25922	*K. pneumoniae* ATCC 700603	*P. mirabilis* SCDR1	*P. mirabilis* ATCC 29906
50	Growth	Growth	Growth	Growth	Growth	Growth	Growth
100	Growth	Growth	Growth	Growth	No Growth	Growth	Growth
150	Growth	No Growth	Growth	No Growth	No Growth	Growth	Growth
200	Growth	No Growth	Growth	No Growth	No Growth	Growth	Growth
250	No Growth	No Growth	No Growth	No Growth	No Growth	Growth	Growth
300	No Growth	No Growth	No Growth	No Growth	No Growth	Growth	Growth
350	No Growth	No Growth	No Growth	No Growth	No Growth	Growth	Growth
400	No Growth	No Growth	No Growth	No Growth	No Growth	Growth	Growth
450	No Growth	No Growth	No Growth	No Growth	No Growth	Growth	Growth
500	No Growth	No Growth	No Growth	No Growth	No Growth	Growth	No Growth
550	No Growth	No Growth	No Growth	No Growth	No Growth	No Growth	No Growth
600	No Growth	No Growth	No Growth	No Growth	No Growth	No Growth	No Growth
650	No Growth	No Growth	No Growth	No Growth	No Growth	No Growth	No Growth
700	No Growth	No Growth	No Growth	No Growth	No Growth	No Growth	No Growth

*S. aureus*: 250 ppm/7.5

*P. aeruginosa*: 150 ppm/7.5

*E. cloacae*: 250 ppm/7.5

*P. mirabilis* SCDR1: 550 ppm/7.5

*P. mirabilis* ATCC: 500 ppm/7.5

**Table 3:** Resistance of *P. mirabilis* SCDR1 against silver and Nanosilver composite assessed by desk diffusion method

Sample ID	Zone Of Inhibition (cm)	Zone Of Inhibition (cm)	Zone Of Inhibition (cm)
	*S. aureus*	*P. aeruginosa*	*P. mirabilis* SCDR1
A	0.9 cm	0.8 cm	No. Inhibition
B	0.9 cm	0.9 cm	No. Inhibition
C	0.8 cm	0.9 cm	No. Inhibition
D	0.8 cm	0.9 cm	No. Inhibition
E	0.9 cm	0.9 cm	No. Inhibition
F	0.8 cm	0.8 cm	No. Inhibition
G	0.7 cm	0.7 cm	No. Inhibition
H	0.9 cm	0.9 cm	No. Inhibition
I	0.9 cm	1.0 cm	No. Inhibition
J	0.9 cm	1.0 cm	No. Inhibition
K	0.8 cm	0.6 cm	No. Inhibition
L	0.8 cm	0.8 cm	No. Inhibition
M	0.9 cm	0.8 cm	No. Inhibition
N	0.9 cm	0.9 cm	No. Inhibition
O	1.0 cm	0.9 cm	No. Inhibition
P	0.8 cm	0.8 cm	No. Inhibition
Q	0.9 cm	0.7 cm	No. Inhibition
R	0.9 cm	0.8 cm	No. Inhibition
S	0.8 cm	0.9 cm	No. Inhibition
T	1.0 cm	0.9 cm	No. Inhibition
U	0.8 cm	0.8 cm	No. Inhibition
V	0.9 cm	0.8 cm	No. Inhibition
W	0.9 cm	0.8 cm	No. Inhibition
X	1.0 cm	0.8 cm	No. Inhibition
Y	0.8 cm	0.8 cm	No. Inhibition
Z	0.7 cm	0.7 cm	No. Inhibition
A1	0.8 cm	0.7 cm	No. Inhibition
B2	0.9 cm	0.7 cm	No. Inhibition
C3	0.9 cm	0.8 cm	No. Inhibition
D4	0.6 cm	NA	No. Inhibition
Silvercel	1.3 cm	1.4 cm	No. Inhibition
Sorbsan silver	1.9 cm	2.0 cm	No. Inhibition
Colactive® Plus Ag	1.5 cm	2.0 cm	No. Inhibition
Exsalt™ SD7	1.5 cm	1.5 cm	No. Inhibition
Puracol ® Plus Ag	1.4 cm	2.0 cm	No. Inhibition
Actisorb® Silver 220	0.9 cm	1.2 cm	No. Inhibition

### General genome features

Data from our draft genome of *P. mirabilis* SCDR1 was deposited in the NCBI-GenBank and was assigned accession number LUFT00000000. The bacterial bioinformatics database and analysis resource (PATRIC) gene annotation analysis showed the presence 308 unique genes of the biosynthesis of secondary metabolites such as tetracycline, Streptomycin, Novobiocin, and Betalain. It is also noteworthy that Xenobiotics Biodegradation and Metabolism pathways also maintained a high number of dedicated unique gene (245) (Additional files 1 and 2: Tables S1 and S2).

### Pathogen identification and phylogenetic analysis

**Fig. 1:**
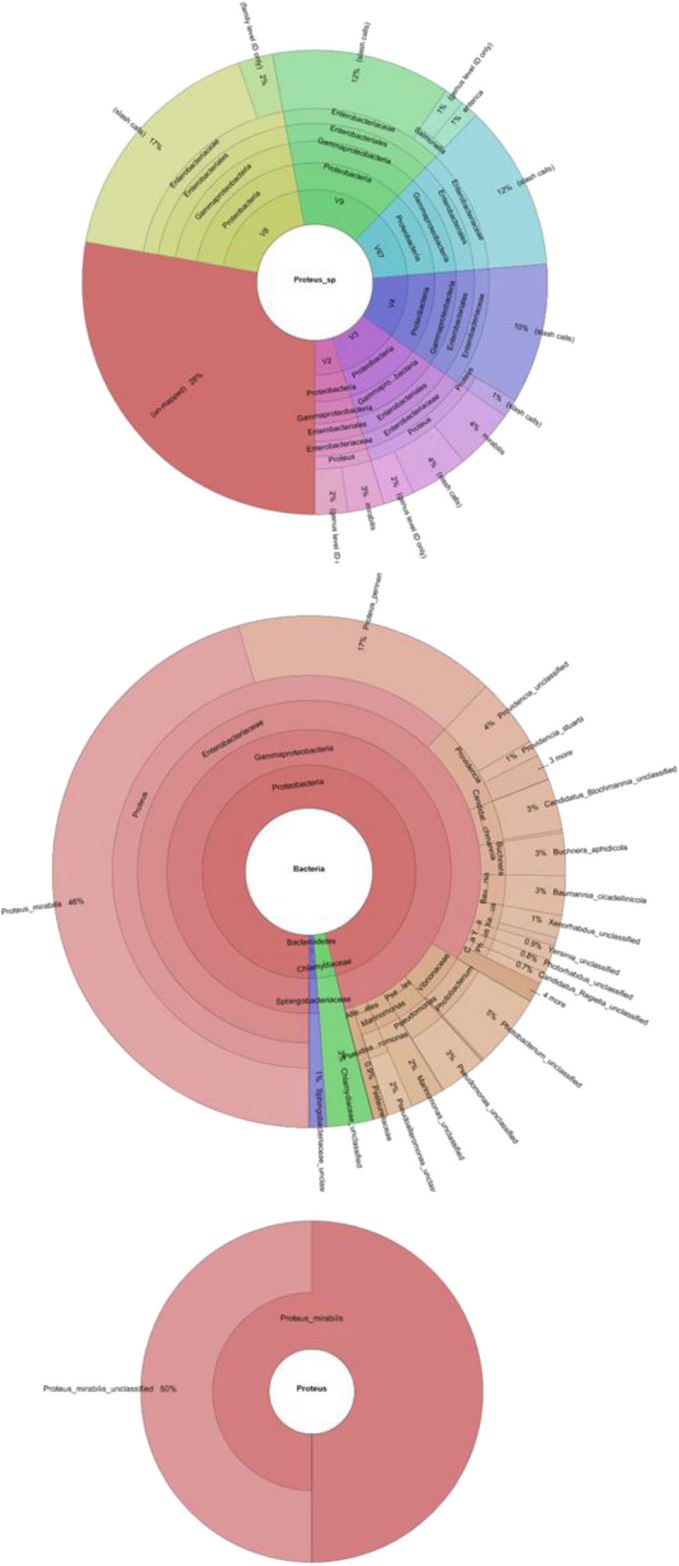
Metaphlan primary identification of the tested taxon

**Fig. 2:**
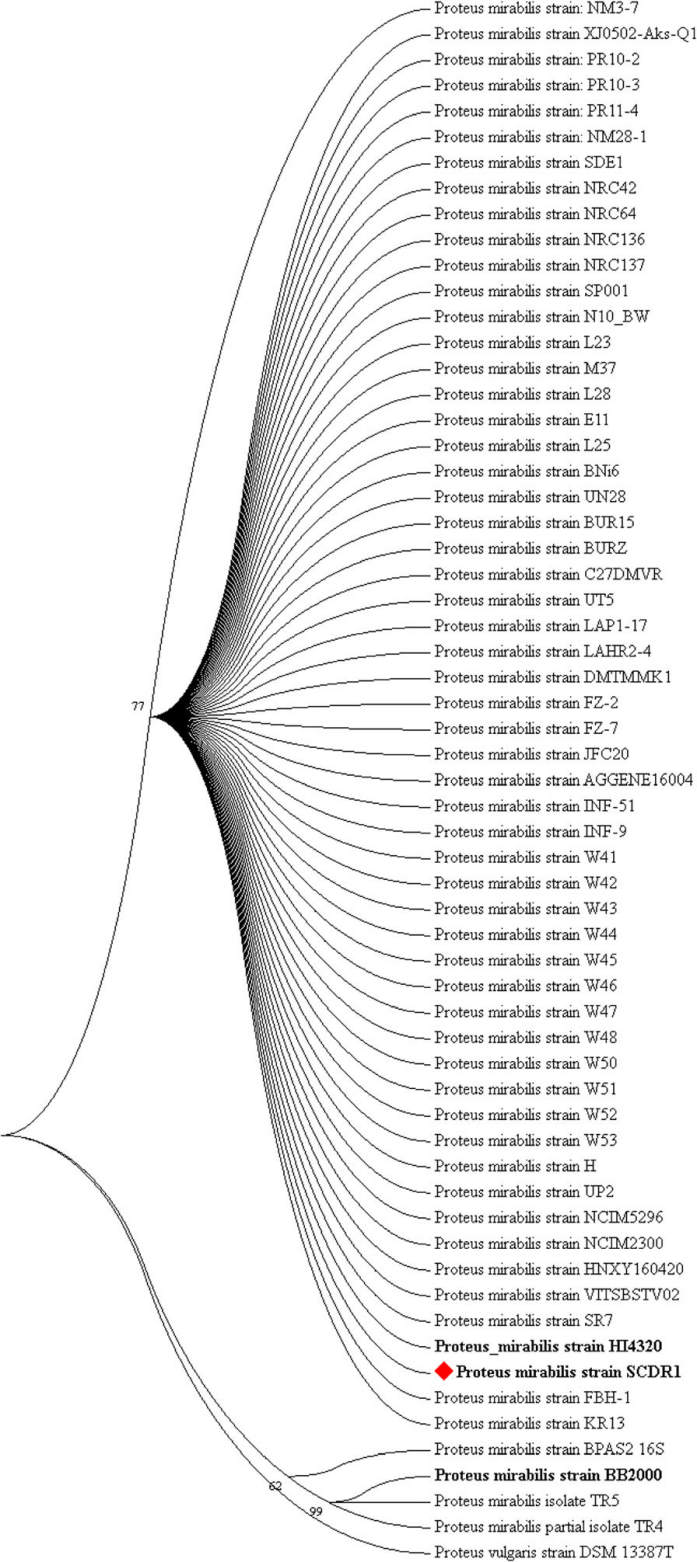
16S rDNA based Maximum likelihood phylogenetic tree of *Proteus mirabilis* spices including Pm-SCDR1 isolate

**Fig. 3:**
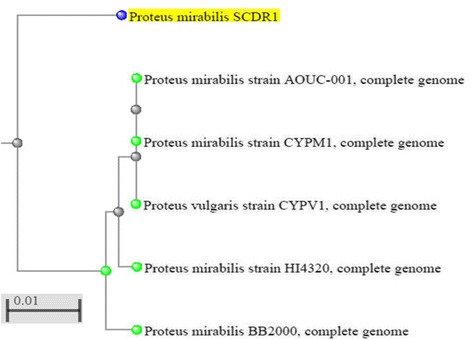
Whole genome Neighbor joining phylogenetic tree of *Proteus mirabilis* spices including Pm-SCDR1 isolate

**Fig. 4:**
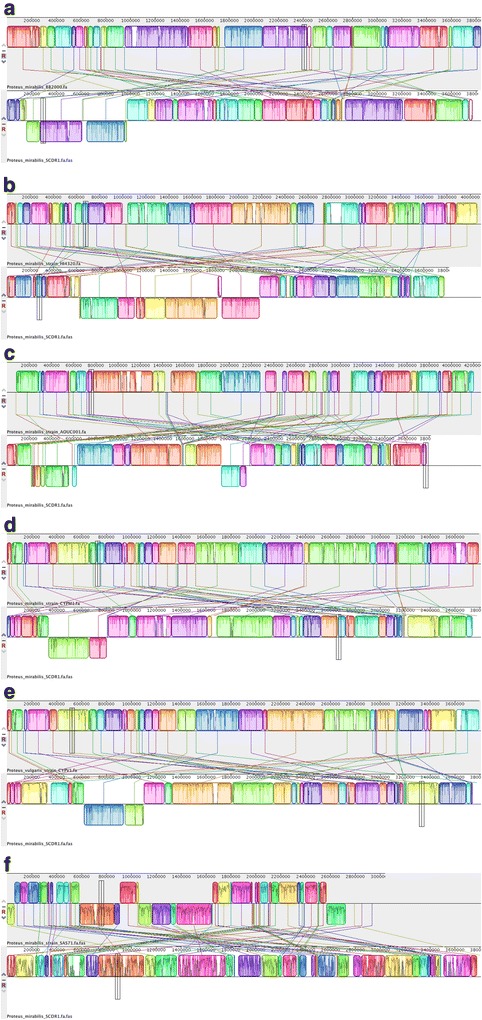
Pair-wise Whole Genome Alignment of *P. mirabilis* strain SCDR1 against reference genomes. **a** *P. mirabilis BB200* and *P. mirabilis* SCDR1 Mauve whole genome alignment, **b** *P. mirabilis HI4320* and *P. mirabilis* SCDR1, **c** *P. mirabilis* AOUC001 and *P. mirabilis* SCDR1, **d** *P. mirabilis* CYPM1 and *P. mirabilis* SCDR1, **e** *P. vulgaris* CYPV1 and *P. mirabilis* SCDR1, **f** *P. mirabilis* SAS71 and *P. mirabilis* SCDR1 Mauve whole genome alignment

**Fig. 5:**
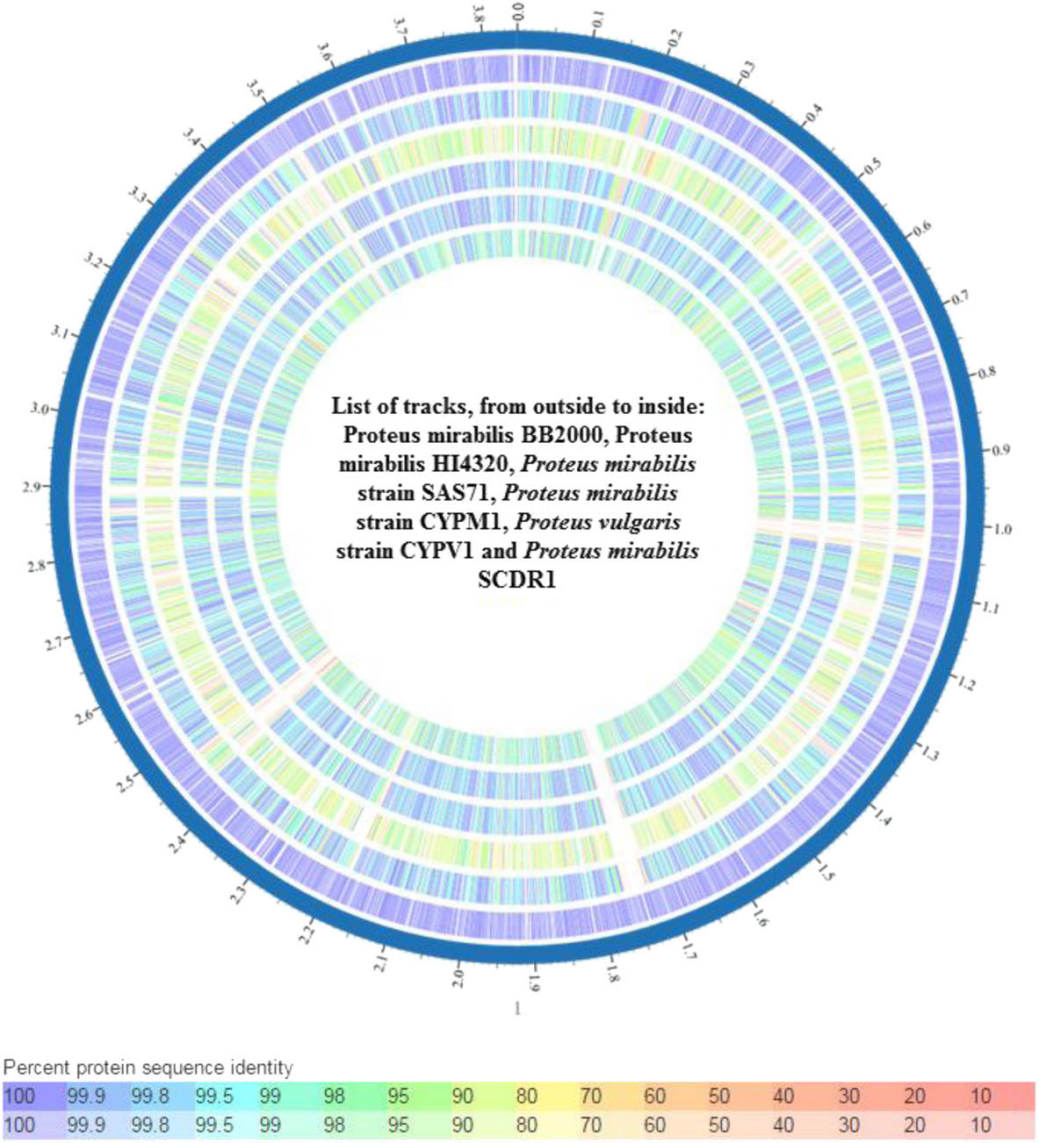
Whole genome phylogeny based proteomic comparison among *Proteus mirabilis* strains

As previously stated, biochemical identification of the isolate confirmed the identity of our isolate as belonging to the *Proteus mirabilis* species. Moreover, Primary analysis of Metaphlan showed that *Proteus mirabilis* is the most dominant species in the sample (Fig. 1). The appearance of other bacterial species in the Metaphlan diagram is explained by the genomic homology similarity of other bacteria to *Proteus mirabilis. P. mirabilis* SCDR1 genome showed high similarly, 92.07%, to the genome of *P. mirabilis* strain BB2000 followed by *P. mirabilis* strain C05028 (90.99%) and *P. mirabilis* strain PR03 (89.73%) (Table 4). A similar scenario was observed when constructing the phylogenetic relationship between our isolate and other *Proteus mirabilis* available in the NCBI- GenBank. 16Sr DNA-based maximum likelihood phylogenetic tree (Fig. 2) showed that our isolate is located within a large clade that contains the majority of *Proteus mirabilis* strains and isolates. In addition, *P. mirabilis* SCDR1 was shown to be closely related to the reference strain *P. mirabilis* HI4320 compared with *P. mirabilis* BB2000, which is located in another clade of four Proteus *mirabilis* taxa. On the contrary, the whole genome Neighbor-joining phylogenetic tree of *Proteus mirabilis* spices including *P. mirabilis* SCDR1 isolate (Fig. 3), showed that our isolate was more closely related to *P. mirabilis* BB2000 compared with the reference strain/genome *P. mirabilis* HI4320. However, Fig. 4 showed that *P. mirabilis* SCDR1 exhibited obvious genetic divergence from other *Proteus mirabilis* species. Similar results were observed when performing pairwise pair-wise whole genome alignment of *P. mirabilis* strain SCDR1 against reference genomes (Fig. 4). This was also confirmed with the clear divergence among *P. mirabilis* SCDR1 *Proteus mirabilis* species at the proteomic level (Fig. 5).

**Table 4:** Comparison of *Proteus mirabilis* SCDR1 to complete and draft reference genomes of *Proteus mirabilis*

NCBI ID	Reference	Ref Size	Gaps sum length	Gaps > = 100 bp	Bases sum length	Bases >500 bp	% Reference
Completed Genomes							
NC_010554.1	*Proteus mirabilis* HI4320	4,063,606	555,251	549,285	3,508,355	3,472,919	86.33
NC_010555.1	*Proteus mirabilis* plasmid pHI4320	36,289	36,289	36,289	0	0	0
NC_022000.1	Proteus mirabilis BB2000	3,846,754	304,708	298,947	3,542,046	3,510,682	92.07
Draft Genomes							
NZ_ACLE00000000	*Proteus mirabilis* ATCC_29,906	4,027,100	565,180	560,679	3,461,920	3,432,786	85.96
NZ_ANBT00000000	*Proteus mirabilis* C05028	3,817,619	343,688	338,218	3,473,931	3,445,432	90.99
NZ_AORN00000000	*Proteus mirabilis* PR03	3,847,612	394,926	390,203	3,452,686	3,430,536	89.73
NZ_AMGU00000000	*Proteus mirabilis* WGLW4	3,960,485	474,704	469,864	3,485,781	3,458,264	88.01
NZ_AMGT00000000	*Proteus mirabilis* WGLW6	4,101,891	606,773	601,555	3,495,118	3,461,467	85.20

### Bacterial pathogenic and virulence factors

Pathogenomics analysis using PathogenFinder 1.1 showed that our input organism was predicted as a human pathogen, and the probability of being a human pathogen was 0.857. *P. mirabilis* SCDR1 comparative proteome analysis showed 35 matched hits from pathogenic families and only one hit from non-pathogenic families (Additional file 3: Table S3). In addition, genome analysis showed that *P. mirabilis* SCDR1 isolate contains numerous virulence factor genes and/or operons that marks it out to be a virulent pathogenic bacterium. These virulence factors include swarming behavior, mobility (flagellae), adherence, toxin and hemolysin production, Urease, Quorum sensing, iron acquisition systems, proteins that function in immune evasion, cell invasion and biofilm formation, stress tolerance factors, and chemotaxis related factors (Additional file 4: Table S4).

### Proteus Mirabilis SCDR1 Resistome

#### Antibiotic resistance

**Fig. 6:**
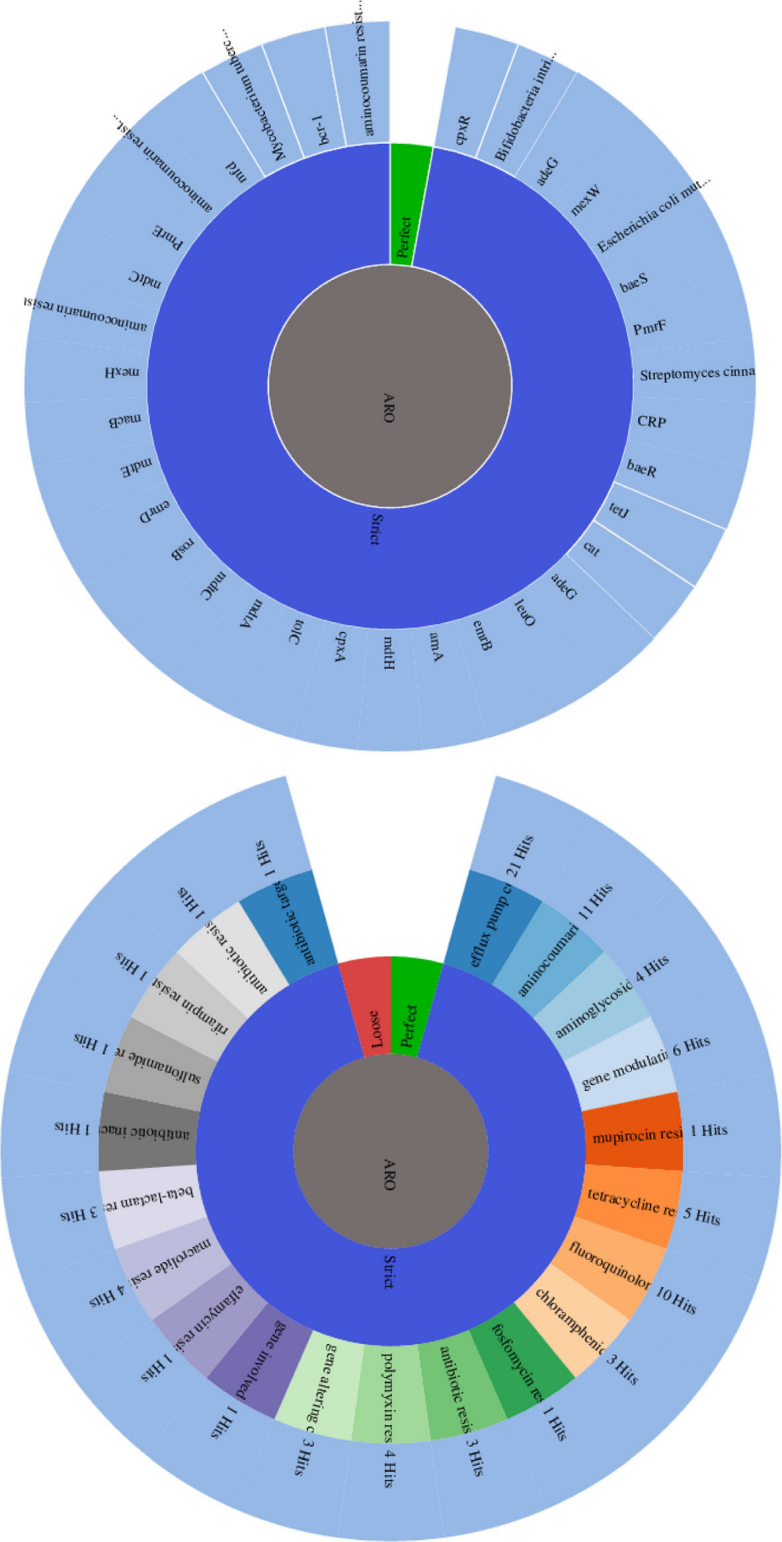
Antibiotic Resistance strict gene and function analysis for Proteus mirabilis SCDR1

**Fig. 7:**
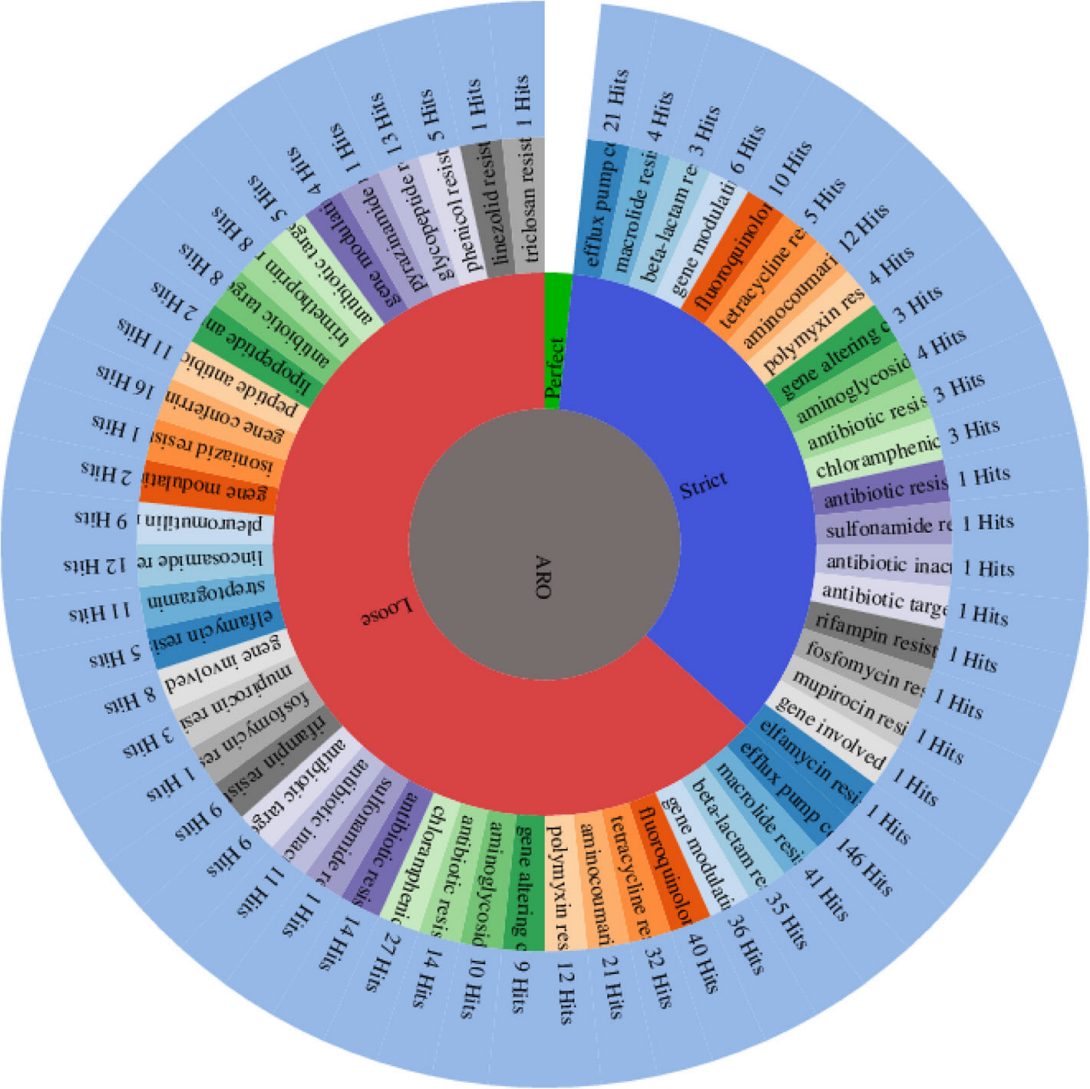
Antibiotic Resistance lose functional categories analysis for Proteus mirabilis SCDR1

Antibiotic resistance identification Perfect and Strict analysis using Resistance Gene Identifier (RGI) showed that P. mirabilis SCDR1 isolate contains 34 antibiotic resistance genes that serve in 21 antibiotic resistance functional categories (Additional file 5: Table S5 and Fig. 6). Meanwhile, using the less strict (Loose) antibiotic resistance identification criteria identified 3750 hits in P. mirabilis SCDR1 genome that represent potential AROs (Antibiotic Resistance Ontology) that fall into 59 antibiotic resistance functional categories (Fig. 7) of which 38 are considered to lose antibiotic resistance functional categories. Modified loose antibiotic resistance identification criteria, by removing all hits with objectionable e-values, lead to a number of 366 antibiotic resistance related hits (Additional file 6: Table S6 and Fig. 7). Manual genome annotation based mining resulted in the identification of 64 drug resistance related proteins in *P. mirabilis* SCDR1 genome (Additional file 7: Table S7).

#### Proteus Mirabilis comparative genomics based resistome analysis

**Fig. 8:**
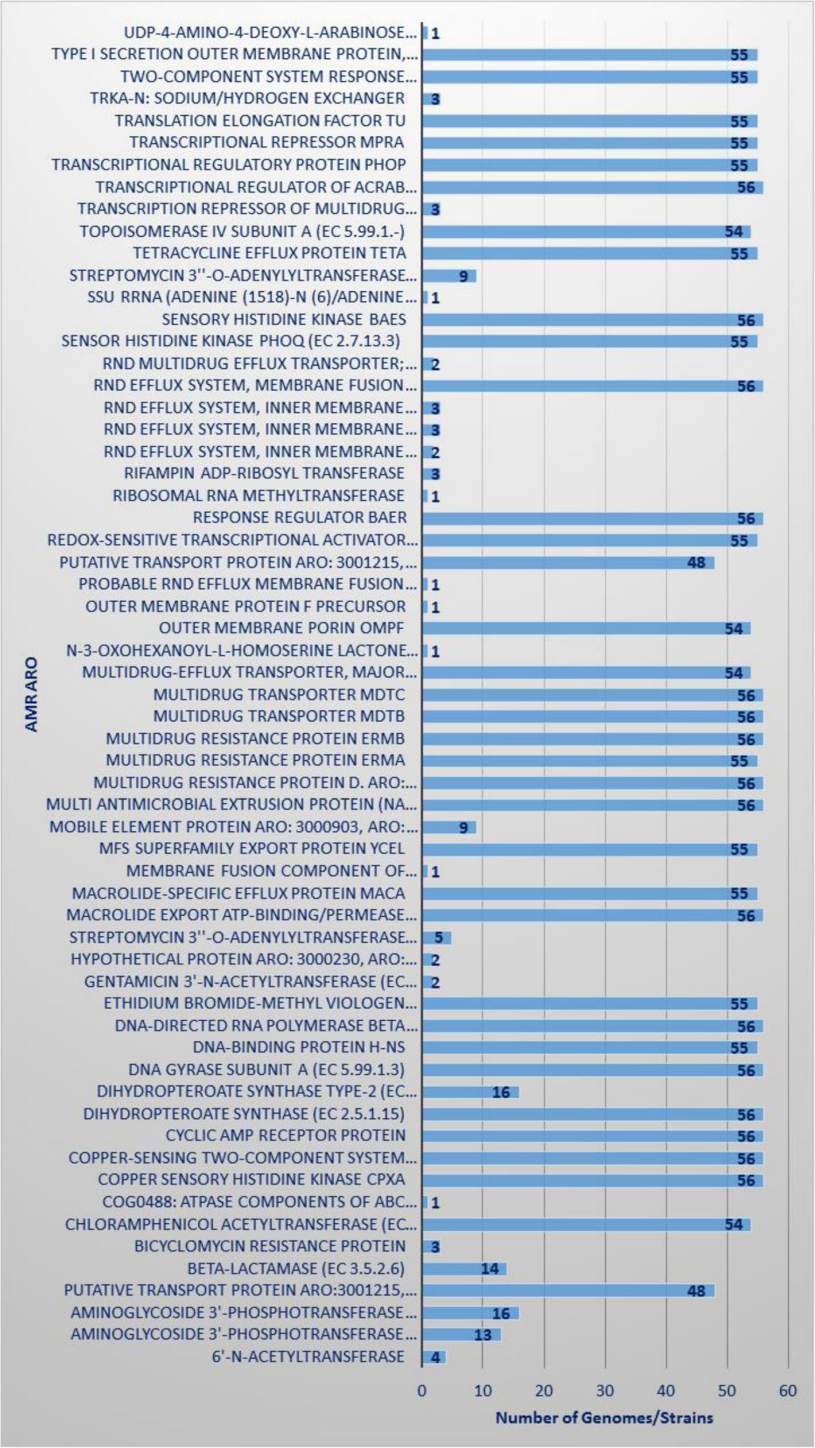
Species wide *Proteus mirabilis* antibiotic resistome constituents

We performed a species-wide antibiotic resistome constituent analysis of *P. mirabilis.* All available *P. mirabilis* genomes, including the *P. mirabilis* SCDR1 genome, were included in this analysis (Table 5). Results of our analysis (Table 6 and Fig. 8) showed that the number of the observed antimicrobial resistance based ontologies (AMRO) in *P. mirabilis* genomes is 61. Only 16 AMROs were observed amongst all the studied 56 *P. mirabilis* genomes. Meanwhile, 13 AMROs were observed amongst 55 *P. mirabilis* genomes. In addition, only four AMROs were observed amongst 54 *P. mirabilis* genomes and two AMROs were observed amongst 48 *P. mirabilis* genomes. This suggests that the core constituent of antibiotic resistome of *P. mirabilis* species is made up of 35 AMROs (Table 6). On the other hand, eight AMROs were detected only in one *P. mirabilis* genome. For example, the membrane fusion component of tripartite multidrug resistance system was only observed in our *P. mirabilis* SCDR1 genome.

**Table 5:** *Proteus mirabilis* genomes represented in the species wide comparative genomics antibiotic resistance analysis

Genome/Strain Name	Genome Status	GenBank Accessions
*P. mirabilis* ATCC 29906	WGS	ACLE00000000
*P. mirabilis* BB2000	Complete	CP004022.1
*P. mirabilis* C05028	WGS	ANBT00000000
*P. mirabilis* HI4320	Complete	AM942759,AM942760
*P. mirabilis* PR03	WGS	AORN00000000
*P. mirabilis* SCDR1	WGS	LUFT00000000
*P. mirabilis* WGLW4	WGS	AMGU00000000
*P. mirabilis* WGLW6	WGS	AMGT00000000
*P. mirabilis* strain 1114_PMIR	WGS	JWCS01000000
*P. mirabilis* strain 1134_PMIR	WGS	JWBY01000000
*P. mirabilis* strain 1150_PMIR	WGS	JWBG01000000
*P. mirabilis* strain 1166_PMIR	WGS	JWAP01000000
*P. mirabilis* strain 127_PMIR	WGS	JVWE01000000
*P. mirabilis* strain 1293_PMIR	WGS	JVVD01000000
*P. mirabilis strain* 1310_PMIR	WGS	JVUH01000000
*P. mirabilis* strain 1313_PMIR	WGS	JVUE01000000
*P. mirabilis* strain1326_PMIR	WGS	JVTO01000000
*P. mirabilis* strain1330_PMIR	WGS	JVTJ01000000
*P. mirabilis* strain 232_PMIR	WGS	JVPB01000000
*P. mirabilis* strain 25,933 GTA	WGS	LANL01000000
*P. mirabilis* strain 25_PMIR	WGS	JVOK01000000
*P. mirabilis* strain 292_PMIR	WGS	JVMQ01000000
*P. mirabilis* strain 360_PMIR	WGS	JVKD01000000
*P. mirabilis* strain 373_PMIR	WGS	JVJQ01000000
*P. mirabilis* strain 418_PMIR	WGS	JVHX01000000
*P. mirabilis* strain 429_PMIR	WGS	JVHK01000000
*P. mirabilis* strain 430_PMIR	WGS	JVHI01000000
*P. mirabilis* strain 47_PMIR	WGS	JVFU01000000
*P. mirabilis* strain 50,664,164	WGS	LNHT01000000
*P. mirabilis* strain 51_PMIR	WGS	JVEH01000000
*P. mirabilis* strain 646_PMIR	WGS	JUYT01000000
*P. mirabilis* strain 672_PMIR	WGS	JUXR01000000
*P. mirabilis* strain 68_PMIR	WGS	JUXK01000000
*P. mirabilis* strain AOUC-001	Complete	CP015347
*P. mirabilis* strain ATCC 7002	WGS	JOVJ00000000
*P. mirabilis* strain C02011	WGS	KV388086,KV388087,KV388088, KV388089,KV388090,KV388091, KV388092,LAGU00000000
*P. mirabilis* strain CYPM1	Complete	CP012674
*P. mirabilis* strain FDAARGOS 60	Complete	JTBW01000000
*P. mirabilis* strain FDAARGOS 67	Complete	JTBP01000000
*P. mirabilis* strain FDAARGOS 80	WGS	JTBB01000000
*P. mirabilis* strain FDAARGOS 81	Complete	JTBA01000000
*P. mirabilis* strain FDAARGOS 85	WGS	JTAW01000000
*P. mirabilis* strain GB08	WGS	LQNN00000000
*P. mirabilis* strain GB11	WGS	LQNO00000000
*P. mirabilis* strain GED7834	WGS	KQ960957,KQ960958,KQ960959,KQ960960, KQ960961,KQ960962,KQ960963,KQ960964, KQ960965,KQ960966,KQ960967,KQ960968, KQ960969,KQ960970,KQ960971,KQ960972, KQ960973,KQ960974,KQ960975,KQ960976, KQ960977,KQ960978,KQ960979,KQ960980, KQ960981,KQ960982,KQ960983,KQ960984, KQ960985,KQ960986,KQ960987,KQ960988, KQ960989,KQ960990,KQ960991,KQ960992, KQ960993,KQ960994,KQ960995,KQ960996, KQ960997,KQ960998,KQ960999,KQ961000, KQ961001,KQ961002,KQ961003,KQ961004 ,KQ961005,KQ961006,KQ961007,KQ961008, KQ961009,KQ961010,KQ961011,KQ961012, KQ961013,KQ961014,KQ961015,KQ961016, KQ961017,KQ961018
*P. mirabilis* strain M16	WGS	LQQZ00000000
*P. mirabilis* strain NIVEDI3-PG74	WGS	LWDB00000000
*P. mirabilis* strain NO-051/03	WGS	LGAY01000000
*P. mirabilis* strain PM593	WGS	JSUP01000000
*P. mirabilis* strain PM655	WGS	JSUO01000000
*P. mirabilis* strain PM_125	WGS	LWUL00000000
*P. mirabilis* strain PM_178	WGS	LWUM00000000
*P. mirabilis* strain Pm-Oxa48	WGS	JSCB01000000
*P. mirabilis* strain Pr2921	WGS	LGTA00000000
*P. mirabilis* strain SAS71	WGS	LDIU01000000
*P. mirabilis* strain Wood	WGS	LTBK00000000

**Table 6:** Species wide *Proteus mirabilis* antibiotic resistome constituents

Antimicrobial Resistance based ontology (AMRO)	Number of Genomes shared AMRO
6′-N-acetyltransferase	4
Aminoglycoside 3′-phosphotransferase @ Streptomycin 3′-kinase StrA	13
Aminoglycoside 3′-phosphotransferase	16
Putative transport protein ARO:3,001,215, ARO:1,000,001	48
Beta-lactamase	14
Bicyclomycin resistance protein	3
Chloramphenicol acetyltransferase	54
COG0488: ATPase components of ABC transporters with duplicated ATPase domains	1
Copper sensory histidine kinase CpxA	56
Copper-sensing two-component system response regulator CpxR	56
Cyclic AMP receptor protein	56
Dihydropteroate synthase	56
Dihydropteroate synthase type-2 @ Sulfonamide resistance protein	16
DNA gyrase subunit A	56
DNA-binding protein H-NS	55
DNA-directed RNA polymerase beta subunit	56
Ethidium bromide-methyl viologen resistance protein EmrE	55
Gentamicin 3′-N-acetyltransferase	2
Hypothetical protein ARO: 3,000,230, ARO: 1,000,001	2
Streptomycin 3″-O-adenylyltransferase @ Spectinomycin 9-O-adenylyltransferase	5
Macrolide export ATP-binding/permease protein MacB	56
Macrolide-specific efflux protein MacA	55
Membrane fusion component of tripartite multidrug resistance system	1
MFS superfamily export protein YceL	55
Mobile element protein ARO: 3,000,903, ARO: 1,000,001	9
Multi antimicrobial extrusion protein (Na (+)/drug antiporter), MATE family of MDR efflux pumps	56
Multidrug resistance protein D. ARO: 3,000,309, ARO: 1,000,001	56
Multidrug resistance protein ErmA	55
Multidrug resistance protein ErmB	56
Multidrug transporter MdtB	56
Multidrug transporter MdtC	56
Multidrug-efflux transporter, major facilitator superfamily (MFS)	54
N-3-oxohexanoyl-L-homoserine lactone quorum-sensing transcriptional activator	1
Outer membrane porin OmpF	54
Outer membrane protein F precursor	1
Probable RND efflux membrane fusion protein	1
Putative transport protein ARO: 3,001,215, ARO: 1,000,001	48
Redox-sensitive transcriptional activator SoxR	55
Response regulator BaeR	56
Ribosomal RNA methyltransferase	1
Rifampin ADP-ribosyl transferase	3
RND efflux system, inner membrane transporter ARO: 3,000,216, ARO: 1,000,001	2
RND efflux system, inner membrane transporter: Aminoglycoside, Glycylcycline, Beta_lactam, Macrolide, Acriflavin	3
RND efflux system, inner membrane transporter Aminoglycoside, Glycylcycline, Beta_lactam, Macrolide, Acriflavin ARO: 3,000,216, ARO: 1,000,001	3
RND efflux system, membrane fusion protein (acrA, ARO: 1,000,001, ARO: 3,000,207) OR (mdtA, ARO: 1,000,001, ARO: 3,000,792)	56
RND multidrug efflux transporter; Acriflavin resistance protein	2
Sensor histidine kinase PhoQ	55
Sensory histidine kinase BaeS	56
SSU rRNA (adenine (1518)-N (6)/adenine (1519)-N (6))-dimethyltransferase	1
Streptomycin 3″-O-adenylyltransferase @ Spectinomycin 9-O-adenylyltransferase (spectinomycin, streptomycin) (ARO: 1,000,001, ARO: 3,000,232) (tobramycin,gentamicin, dibekacin, sisomicin, kanamycin)	9
Tetracycline efflux protein TetA	55
Topoisomerase IV subunit A	54
Transcription repressor of multidrug efflux pump acrAB operon, TetR (AcrR) family	3
Transcriptional regulator of acrAB operon, AcrR	56
Transcriptional regulatory protein PhoP	55
Transcriptional repressor MprA	55
Translation elongation factor Tu	55
TrkA-N: Sodium/hydrogen exchanger	3
Two-component system response regulator OmpR	55
Type I secretion outer membrane protein, TolC precursor	55
UDP-4-amino-4-deoxy-L-arabinose formyltransferase/ UDP-glucuronic acid oxidase (UDP-4-keto-hexauronic acid decarboxylating)	1

#### Consensus *P. mirabilis*-SCDR1 antibiotic Resistome

Table 7 displays the consensus *P. mirabilis*-SCDR1 antibiotic resistome. Genomics analysis of *P. mirabilis*-SCDR1 63 contigs showed that our isolates contained genetic determinants for tetracycline resistance (tetAJ), fluoroquinolones resistance (gyrA, parC and parE), sulfonamide resistance (folP), daptomycin and rifamycin resistance (rpoB), elfamycin antibiotics resistance (tufB), Chloramphenicol (cpxR, cpxA and cat), ethidium bromide-methyl viologen resistance protein (emrE) and polymyxin resistance (phoP). In addition, several multidrug resistance efflux systems and complexes such as MdtABC-TolC, MacAB-TolC, AcrAB-TolC, EmrAB-TolC, AcrEF-TolC and MATE.

**Table 7:** Consensus *P. mirabilis*-SCDR1 antibiotic Resistome

Source	Source Organism	Gene	Product	Function	Query Coverage	Identity	E-value
ARDB	*P. mirabilis* ATCC 29906	tetAJ	Tetracycline efflux protein TetA	Major facilitator superfamily transporter, tetracycline efflux pump.	97	95	0
CARD	*P. mirabilis* BB2000	tetAJ	Tetracycline efflux protein TetA	Major facilitator superfamily transporter, tetracycline efflux pump.	97	94	0
ARDB	*P. mirabilis* HI4320	tetAJ	Tetracycline efflux protein TetA	Major facilitator superfamily transporter, tetracycline efflux pump.	80	99	2e-74
CARD	*P. mirabilis* BB2000	gyrA	DNA gyrase subunit A (EC 5.99.1.3)	Point mutation of Escherichia coli gyrA resulted in the lowered affinity between fluoroquinolones and gyrA. Thus, conferring resistance	98	99	0
CARD	*P. mirabilis* BB2000	baeR	Response regulator BaeR	BaeR is a response regulator that promotes the expression of MdtABC and AcrD efflux complexes.	100	99	2e-171
CARD	*P. mirabilis* BB2000	baeS	Sensory histidine kinase BaeS	BaeS is a sensor kinase in the BaeSR regulatory system. While it phosphorylates BaeR to increase its activity.	100	99	0
CARD	*P. mirabilis* BB2000	mdtC	Multidrug transporter MdtC	MdtC is a transporter that forms a hetero-multimer complex with MdtB to form a multidrug transporter. MdtBC is part of the MdtABC-TolC efflux complex.	100	99	0
CARD	*P. mirabilis* BB2000	mdtB	Multidrug transporter MdtB	MdtB is a transporter that forms a heteromultimer complex with MdtC to form a multidrug transporter. MdtBC is part of the MdtABC-TolC efflux complex.	100	99	0
CARD	*P. mirabilis* BB2000	mdtA	RND efflux system, membrane fusion protein	MdtA is the membrane fusion protein of the multidrug efflux complex mdtABC.	100	98	0
CARD	*P. mirabilis* BB2000	folP	Dihydropteroate synthase (EC 2.5.1.15)	Point mutations in dihydropteroate synthase folP prevent sulfonamide antibiotics from inhibiting its role in folate synthesis, thus conferring sulfonamide resistance.	100	100	0
CARD	*P. mirabilis* BB2000	soxR	Redox-sensitive transcriptional activator SoxR	SoxR is a sensory protein that upregulates soxS expression in the presence of redox-cycling drugs. This stress response leads to the expression many multidrug efflux pumps.	100	100	0
CARD	*Shigella dysenteriae* Sd197	ompR	Two-component system response regulator OmpR	Transcriptional regulatory protein	99	87	0
CARD	*P. mirabilis* BB2000	emrR	Transcriptional repressor MprA	EmrR is a negative regulator for the EmrAB-TolC multidrug efflux pump in E. coli. Mutations lead to EmrAB-TolC overexpression.	100	100	0
CARD	*P. mirabilis* BB2000	emrA	Multidrug resistance protein ErmA	EmrA is a membrane fusion protein, providing an efflux pathway with EmrB and TolC between the inner and outer membranes of E. coli, a Gram-negative bacterium.	95	96	0
CARD	*P. mirabilis* BB2000	acrE	Membrane fusion component of tripartite multidrug resistance system	AcrEF-TolC is a tripartite multidrug efflux system similar to AcrAB-TolC and found in Gram-negative bacteria. AcrE is the membrane fusion protein, AcrF is the inner membrane transporter, and TolC is the outer membrane channel protein.	100	98	3e-44
CARD	*P. mirabilis* BB2000	emrB	Multidrug resistance protein ErmB	emrB is a translocase in the emrB -TolC efflux protein in E. coli. It recognizes substrates including carbonyl cyanide m-chlorophenylhydrazone (CCCP), nalidixic acid, and thioloactomycin.	100	99	0
CARD	*P. mirabilis* BB2000	rpoB	DNA-directed RNA polymerase beta subunit (EC 2.7.7.6)	Mutations in rpoB gene confers antibiotic resistance (Daptomycin and Rifamycin)	100	99	0
CARD	*P. mirabilis* BB2000	tufB	Translation elongation factor Tu	Sequence variants of elongation factor Tu confer resistance to elfamycin antibiotics.	100	100	1e-43
CARD	*P. mirabilis* BB2000	cpxA	Copper sensory histidine kinase CpxA	cpxA mutant confer resistant to amikacin	94	99	0
CARD	*P. mirabilis* BB2000	cpxR	Copper-sensing two-component system response regulator CpxR	CpxR is a regulator that promotes acrD expression when phosphorylated by a cascade involving CpxA, a sensor kinase. Cefepime and chloramphenicol	100	100	0
CARD	*P. mirabilis* BB2000	emrD	Multidrug resistance protein D	EmrD is a multidrug transporter from the Major Facilitator Superfamily (MFS) primarily found in Escherichia coli. EmrD couples efflux of amphipathic compounds with proton import across the plasma membrane.	100	99	0
CARD	*P. mirabilis* BB2000	macA	Macrolide-specific efflux protein MacA	MacA is a membrane fusion protein that forms an antibiotic efflux complex with MacB and TolC.	100	99	3e-177
CARD	*P. mirabilis* BB2000	macB	Macrolide export ATP-binding/permease protein MacB (EC 3.6.3.-)	MacB is an ATP-binding cassette (ABC) transporter that exports macrolides with 14- or 15- membered lactones. It forms an antibiotic efflux complex with MacA and TolC.	100	98	0
ARDB	*P. mirabilis* ATCC 29906	cat	Chloramphenicol acetyltransferase (EC 2.3.1.28)	Group A chloramphenicol acetyltransferase, which can inactivate chloramphenicol.	99	93	6e-150
CARD	*P. mirabilis* BB2000	cat	Chloramphenicol acetyltransferase (EC 2.3.1.28)	Group A chloramphenicol acetyltransferase, which can inactivate chloramphenicol.	99	93	4e-151
CARD	*P. mirabilis* BB2000	acrR	Transcription repressor of multidrug efflux pump acrAB operon, TetR (AcrR) family	AcrR is a repressor of the AcrAB-TolC multidrug efflux complex. AcrR mutations result in high level antibiotic resistance.	100	95	9e-25
CARD	*P. mirabilis* BB2000	acrR	Transcriptional regulator of acrAB operon, AcrR	AcrR is a repressor of the AcrAB-TolC multidrug efflux complex. AcrR mutations result in high level antibiotic resistance.	93	95	2e-114
CARD	*P. mirabilis* BB2000	acrA	RND efflux system, membrane fusion protein	Protein subunit of AcrA-AcrB-TolC multidrug efflux complex. AcrA represents the periplasmic portion of the transport protein.	100	99	0
CARD	*P. mirabilis* BB2000	mdtK	Multi antimicrobial extrusion protein (Na(+)/drug antiporter), MATE family of MDR efflux pumps	A multidrug and toxic compound extrusions (MATE) transporter conferring resistance to norfloxacin, doxorubicin and acriflavine.	98	99	3e-164
CARD	*Salmonella enterica subsp. enterica serovar Agona* str. SL483	hns	DNA-binding protein H-NS	H-NS is a histone-like protein involved in global gene regulation in Gram-negative bacteria. It is a repressor of the membrane fusion protein genes acrE, mdtE, and emrK as well as nearby genes of many RND-type multidrug exporters.	100	80	0
CARD	*P. mirabilis* BB2000	tufB	Translation elongation factor Tu	Sequence variants of elongation factor Tu confer resistance to elfamycin antibiotics.	100	99	0
CARD	*Shigella dysenteriae* Sd197	crp	Cyclic AMP receptor protein	CRP is a global regulator that represses MdtEF multidrug efflux pump expression.	100	98	0
CARD	*P. mirabilis* BB2000	emrE	Ethidium bromide-methyl viologen resistance protein EmrE	EmrE is a small multidrug transporter that functions as a homodimer and that couples the efflux of small polyaromatic cations from the cell with the import of protons down an electrochemical gradient. EmrE is found in E. coli and P. aeruginosa.	100	99	6e-73
CARD	*P. mirabilis* BB2000	mdtK	Multi antimicrobial extrusion protein (Na(+)/drug antiporter), MATE family of MDR efflux pumps	A multidrug and toxic compound extrusions (MATE) transporter conferring resistance to norfloxacin, doxorubicin and acriflavine.	100	100	2e-113
CARD	*P. mirabilis* BB2000	NIA	Putative transport protein	NIA	100	94	7e-59
CARD	*P. mirabilis* BB2000	NIA	Multidrug resistance protein	NIA	99	96	2e-112
CARD	*P. mirabilis* BB2000	parC	Topoisomerase I subunit A (EC 5.99.1.-)	ParC is a subunit of topoisomerase IV, which decatenates and relaxes DNA to allow access to genes for transcription or translation. Point mutations in ParC prevent fluoroquinolone antibiotics from inhibiting DNA synthesis, and confer low-level resistance. Higher-level resistance results from both gyrA and parC mutations.	99	99	0
CARD	*P. mirabilis* BB2000	parE	Topoisomerase IV subunit B (EC 5.99.1.-)	ParE is a subunit of topoisomerase IV, necessary for cell survival. Point mutations in ParE prevent fluoroquinolones from inhibiting DNA synthesis, thus conferring resistance.	100	99	0
CARD	*P. mirabilis* BB2000	tolC	Type I secretion outer membrane protein, TolC precursor	TolC is a protein subunit of many multidrug efflux complexes in Gram negative bacteria. It is an outer membrane efflux protein and is constitutively open. Regulation of efflux activity is often at its periplasmic entrance by other components of the efflux complex.	100	99	0
CARD	*P. mirabilis* BB2000	mdtH	MFS superfamily export protein YceL	Multidrug resistance protein MdtH	100	99	0
CARD	*P. mirabilis* BB2000	phoP	Transcriptional regulatory protein PhoP	A mutant phoP activates pmrHFIJKLM expression responsible for L-aminoarabinose synthesis and polymyxin resistance, by way of alteration of negative charge	100	99	5e-165
CARD	*P. mirabilis* BB2000	phoQ	Sensor histidine kinase PhoQ (EC 2.7.13.3)	Mutations in Pseudomonas aeruginosa PhoQ of the two-component PhoPQ regulatory system. Presence of mutation confers resistance to colistin	90	99	0
CARD	*P. mirabilis* BB2000	phoQ	Sensor histidine kinase PhoQ (EC 2.7.13.3)	Mutations in Pseudomonas aeruginosa PhoQ of the two-component PhoPQ regulatory system. Presence of mutation confers resistance to colistin	98	98	1e-45

Evidence: BLASTP, NIA: No information available, ARDB: Antibiotic Resistance Genes Database, CARD: Comprehensive Antibiotic Resistance Database

• MdtC: In the absence of MdtB, MdtC can form a homomultimer complex that results in a functioning efflux complex with a narrower drug specificity

• MdtABC-TolC https://card.mcmaster.ca/ontology/37167

• Elongation factor Tu is required for peptide elongation in bacterial protein synthesis

• cpxA http://www.uniprot.org/citations/2185221

• cpxR Srinivasan VB, et al. 2012. PLoS One 7(4): E33777. Role of the two component signal transduction system CpxAR in conferring cefepime and chloramphenicol resistance in Klebsiella pneumoniae NTUH-K2044. (PMID 22496764)

• MacAB-TolC: MacAB-TolC is an ABC efflux pump complex expressed in E. coli and Salmonella enterica. It confers resistance to macrolides, including erythromycin

#### Heavy metal resistance

Table 8 presents *P. mirabilis* SCDR1 heavy metal resistance/binding factors. Numerous genetic determinants for metal resistance were observed in the *P. mirabilis* SCDR1 genome. Several Copper resistance genes/proteins were detected, namely, copA, copB, copC, copD, cueO, cueR, cutC, cutF and CuRO_2_CopA_like1. In addition, gene determinants of Copper/silver efflux system were observed, namely, oprB, oprM and cusC_1. Moreover, several heavy metal resistance proteins and efflux systems were observed, such as magnesium/cobalt efflux protein CorC, metal resistance proteins (AGS59089.1, AGS59090.1 and AGS59091.1), nickel-cobalt-cadmium resistance protein NccB, arsenical pump membrane protein (ArsB permease), Lead, cadmium, zinc and mercury transporting ATPase, outer membrane component of tripartite multidrug resistance system (CusC) and complete *P. mirabilis* tellurite resistance loci (terB, terA, terC, terD, terE, terZ). Furthermore, enzymes involved in heavy metal resistance were also observed such as glutathione S-transferase (gst1, gst, Delta and Uncharacterized), arsenite S-adenosylmethyltransferase (Methyltransferase type 11) and alkylmercury lyase (MerB).

**Table 8:** *P. mirabilis* SCDR1 Heavy Metal Resistance/Binding factors

Annotation	Reference Genome	Accession Number	Gene	Protein ID	AA Length	Corresponding Protein
PATRIC	*P. mirabilis* ATCC 29906	NZ_GG668580	corC	ZP_03842837.1	293	Magnesium/cobalt efflux protein CorC.
RefSeq	*P. mirabilis* BB2000	CP004022	NA	AGS60530.1	305	cation efflux protein (Divalent metal cation (Fe/Co/Zn/Cd) transporter).
PATRIC	*P. mirabilis* ATCC 29906	NZ_GG668576	cueR	ZP_03840921.1	133	MerR-family transcriptional regulator (copper efflux regulator).
RefSeq	*P. mirabilis* BB2000	CP004022	arsB	AGS60689.1	429	Arsenical pump membrane protein (ArsB_permease).
RefSeq	*P. mirabilis* BB2000	CP004022	NA	AGS59089.1 AGS59090.1 AGS59091.1	129 678 243	Metal resistance protein.
PATRIC	*P. mirabilis* ATCC 29906 *P. mirabilis* strain 25,933 GTA	NZ_GG668576 LANL01000027	ahpF NA	ZP_03839875.1 KKC60389.1	521 678	Protein-disulfide reductase.
PATRIC	*P. mirabilis* ATCC 29906	NZ_GG668576 NZ_GG668583	dsbB dsbA	ZP_03840198.1 ZP_03839563.1	174 207	Protein disulfide oxidoreductase.
PATRIC	*P. mirabilis* ATCC 29906 *P. mirabilis* BB2000	NZ_GG668576 NZ_GG668576 NZ_GG668578 CP004022	actP1 copA ppaA zntA	ZP_03840801.1 ZP_03840922.1 ZP_03842696.1 AGS58561.1	829 984 803 796	(zinc/cadmium/mercury/lead-transporting ATPase) (HMA).
PATRIC	*P. mirabilis* ATCC 29906	NZ_GG668578	gloB	ZP_03842342.1	251	hydroxyacylglutathione hydrolase.
RefSeq	*P. mirabilis* strain ATCC 7002	JOVJ01000008	grxA	KGA90223.1	87	Glutaredoxin, GrxA family.
PATRIC	*P. mirabilis* ATCC 29906 *P. mirabilis* strain 1134_PMIR	NZ_GG668576 NZ_GG668576	gst1 gst Delta Uncharacterized	ZP_03840532.1 ZP_03840063.1 PGF_02913068^a^ PGF_00008413^a^	204 203 195 110	Glutathione S-transferase (EC 2.5.1.18).
RefSeq	*P. mirabilis* BB2000	CP004022	cueO	AGS58840.1	526	Multicopper oxidase.
PATRIC	*P. mirabilis* ATCC 29906	NZ_GG668578	NA	ZP_03842149.1	243	FIG00003370: Multicopper polyphenol oxidase.
PATRIC	*P. mirabilis* strain ATCC 7002	JOVJ01000009	yobA	ZP_03839688.1	130	Copper resistance protein (Copper-binding protein CopC (methionine-rich)) [Inorganic ion transport and metabolism].
PATRIC	*P. mirabilis* ATCC 29906	NZ_GG668576	copD	ZP_03839689.1	279	Copper resistance protein.
PATRIC	*P. mirabilis* strain SAS71	LDIU01000481	NA	PGF_00419563	114	Copper resistance protein D.
BRC1	*P. mirabilis* HI4320	NC_010554	NA	NA	300	Putative copper resistance protein, secreted.
PATRIC RefSeq	*P. mirabilis* ATCC 29906	NZ_GG668576	copC	ZP_03839688.1	130	Copper resistance protein CopC.
PATRIC	*E. coli* 7–233-03_S4_C2	JORW01000046	copB	KEN13242.1	296	Copper resistance protein B.
PATRIC	*P. mirabilis* ATCC 29906	NZ_GG668576	cutC	ZP_03839779.1	250	Copper homeostasis protein CutC (Cytoplasmic copper homeostasis protein CutC).
RefSeq	*P. mirabilis* BB2000	CP004022	cop A	AGS60771.1	904	Copper exporting ATPase.
PATRIC	*P. mirabilis* ATCC 29906	NZ_GG668576	cop A	ZP_03840922.1	949	Lead, cadmium, zinc and mercury transporting ATPase (EC 3.6.3.3) (EC 3.6.3.5); Copper-translocating P-type ATPase (EC 3.6.3.4).
RefSeq	*P. mirabilis* strain ATCC 7002	JOVJ01000009	kdpB	KGA89427.1	685	Copper exporting ATPase (potassium-transporting ATPase subunit B).
RefSeq	*P. mirabilis*	WP_012368272.1, WP_020946123.1	copA- CopZ- HMA	WP_012368272 WP_020946123	984	Copper exporting ATPase (Heavy-metal-associated domain (HMA)).
RefSeq	*P. mirabilis* strain ATCC 7002	JOVJ01000005	cueR	KGA91278.1	135	Copper -responsive transcriptional regulator (HTH_MerR-SF Superfamily).
PATRIC	*P. mirabilis* BB2000 *P. mirabilis* strain 1310_PMIR	CP004022 JVUH01000152 JVUH01001396	cutF	ZP_03841587.1 PGF_00241126^a^ PGF_00241126^a^	225 154 78	Copper homeostasis protein CutF precursor/Lipoprotein NlpE involeved in surface adhesion.
PATRIC RefSeq	*P. mirabilis* BB2000	CP004022	terB terA terC terD terE terZ	AGS60978.1 AGS60979.1 AGS60977.1 AGS60976.1 AGS60975.1 AGS60980.1	151 382 341 192 191 194	*P. mirabilis* tellurite resistance loci.
PATRIC RefSeq	*Mycobacterium sp.*	YP_001705575.1 CP002992	ctpC	AEN01737.1	718	Probable cation-transporting ATPase G (ATPase-IB2_Cd).
PATRIC	*P. mirabilis* ATCC 29906	NZ_GG668579	yntB	ZP_03841770.1	325	Nickel transport system permease protein nikB2 (TC 3.A.1.5.3).
PATRIC	*P. mirabilis* ATCC 29906	NZ_GG668579	yntA	ZP_03841771.1	527	Nickel ABC transporter, periplasmic nickel-binding protein nikA2 (TC 3.A.1.5.3).
PATRIC	*P. mirabilis* ATCC 29906	NZ_GG668583	NA	ZP_03839446.1	289	Nickel transport system permease protein NikC (TC 3.A.1.5.3).
PATRIC	*P. mirabilis* ATCC 29906	NZ_GG668583	NA	ZP_03839447.1	269	Nickel transport ATP-binding protein NikD (TC 3.A.1.5.3).
PATRIC	*P. mirabilis* ATCC 29906	NZ_GG668579	yntD	ZP_03841768.1	267	Nickel transport ATP-binding protein nikD2 (TC 3.A.1.5.3).
PATRIC	*P. mirabilis* ATCC 29906	NZ_GG668579	yntE	ZP_03841767.1	203	Nickel transport ATP-binding protein nikE2 (TC 3.A.1.5.3).
PATRIC	*P. mirabilis* ATCC 29906	NZ_GG668579	yntC	ZP_03841769.1	270	Nickel transport system permease protein nikC2 (TC 3.A.1.5.3).
PATRIC	*P. mirabilis* BB2000	CP004022	hybF	AGS58541.1	113	[NiFe] hydrogenase nickel incorporation protein HypA.
PATRIC	*P. mirabilis* ATCC 29906	NZ_GG668578	hybB	ZP_03842517.1	282	[NiFe] hydrogenase nickel incorporation-associated protein HypB.
RefSeq	*C. crescentus* OR37	APMP01000019	NA	ENZ81282.1	723	Copper/silver/heavy metal-translocating P-type ATPase, Cd/Co/Hg/Pb/Zn-transporting.
RefSeq	*Armatimonadetes* bacterium OLB18 *C. gilvus*	JZQX01000123 WP_013884717.1	arsM	KXK16912.1	283	Arsenite S-adenosylmethyltransferase (Methyltransferase type 11).
RefSeq	*R. palustris* TIE-1	NC_011004	NA	YP_001990857.1	973	Heavy metal translocating P-type ATPase (ATPase-IB1_Cu).
RefSeq	*M. ulcerans* str. Harvey	EUA92940.1,	CuRO_2_CopA_like1	EUA92940.1	552	Multicopper oxidase family protein.
RefSeq	*B. mallei* NCTC 10229	NC_008835	oprB	YP_001024205.1	553	Copper/silver efflux system outer membrane protein CusC (outer membrane efflux protein OprB).
RefSeq	*B. pseudomallei* 576	NZ_ACCE01000001	oprM	ZP_03450560.1	558	Copper/silver efflux system outer membrane protein CusC (outer membrane efflux protein OprM).
PATRIC RefSeq	*Achromobacter sp.* strain 2789STDY5608636 *B. pseudomallei* 1710b	CYTV01000008 ABA52627.1	cusC_1	ABA52627	515	Copper/silver efflux system outer membrane protein CusC (RND efflux system outer membrane lipoprotein).
RefSeq	*Achromobacter sp.* strain 2789STDY5608623	CYSZ01000001	NA	CUI29018.1	98	Outer membrane component of tripartite multidrug resistance system (CusC).
RefSeq	R. opacus	WP_012687282.1, BAH48260.1	merB	WP_012687282	334	Alkylmercury lyase (MerB).
PATRIC RefSeq	*B. ubonensis* strain MSMB2185WGS	Q44585.1 LPIU01000068	NA	Q44585 PGF_01102114^a^	379 377	Nickel-cobalt-cadmium resistance protein NccB.
PATRIC	*P. mirabilis* BB2000	CP004022	zntA	AGS58561.1	798	Lead, cadmium, zinc and mercury transporting ATPase (EC 3.6.3.3) (EC 3.6.3.5); Copper-translocating P-type ATPase (EC 3.6.3.4)
PATRIC	*P. mirabilis* BB2000	CP004022	copA	AGS60771.1	949	Lead, cadmium, zinc and mercury transporting ATPase (EC 3.6.3.3) (EC 3.6.3.5); Copper-translocating P-type ATPase (EC 3.6.3.4).
PATRIC	*P. mirabilis* BB2000	CP004022	copA	AGS60770.1	54	Lead, cadmium, zinc and mercury transporting ATPase (EC 3.6.3.3) (EC 3.6.3.5); Copper-translocating P-type ATPase (EC 3.6.3.4).

*NA* Not availbe

^a^PATRIC cross-genus families (PGfams)

## Discussion

*Proteus mirabilis* isolate was observed as mixed culture along with *S. aureus* isolate while testing our produced silver Nanoparticles against several pathogenic *S. aureus* isolates [9]. Whereas other tested Gram positive and negative bacteria showed great sensitivity against silver Nanoparticles, *P. mirabilis*, SCDR1 isolate exhibited extreme resistance. *P. mirabilis* SCDR1 isolate resistant against at least one antibiotic belonging to ansamycins, glycopeptides, fucidanes, cyclic peptides, nitroimidazoles, macrolides, lincosamides, folate pathway inhibitors and aminocoumarin antimicrobial categories. Moreover, our isolate exhibited intrinsic resistance against tetracyclines and polymyxins specific to *P. mirabilis* species [36, 52, 53]. However, fortunately, our isolate was sensitive to several operational antimicrobial categories such as penicillins with b-lactamase inhibitors, extended-spectrum cephalosporins, carbapenems, aminoglycosides, fluoroquinolones and phosphonic acids. In addition, our *P. mirabilis* SCDR1 isolate showed high resistance against colloidal and composite Nanosilver and metallic silver when compared to other tested Gram positive and negative bacterial species, both qualitatively and quantitatively. To the best of our knowledge, this is the first reported case of bacterial spontaneous resistance to colloidal and composite nanosilver. However, Gunawan et al., (2013) reported the occurrence of induced adaptation, of non-targeted environmental *Bacillus* species to antimicrobial Nanosilver [14]. In addition, it was found that bacteria can straightforwardly develop resistance to AgNPs, and this occurs by relatively simple genomic changes [54]. They both showed that a *Bacillus sp.* environmental isolate and an *E.coli* isolate were able to adapt to Nanosilver cytotoxicity upon continued exposure. Nonetheless, as previously stated, *P. mirabilis* SCDR1 exhibited instantaneous resistance against nanosilver without the need for any prolonged exposure. *P. mirabilis SCDR1* demonstrated resistance against colloidal nanosilver assessed either by disk diffusion or by minimal inhibitory concentration methods. While all used concentrations of colloidal Nanosilver have shown strong effects on all tested microorganisms (Table 1), there was no effect on the bacterial growth of *P. mirabilis SCDR1* even at the highest used concentration (200 ppm). Similarly, *P. mirabilis SCDR1* was able to resist ten fold (500 ppm) higher than *K. pneumoniae* (50 ppm)*,* five fold higher than *P. aeruginosa* and *E. coli* (100 ppm) and two and a half fold (200 ppm) higher than *S. aureus* and *E. cloacae* (Table 2). Moreover, while both laboratory prepared and commercially available silver and Nanosilver composite showed a clear effect against both *S. aureus* and *P. aeruginosa,* the most common pathogens of diabetic foot ulcer, not effect was observed against *P. mirabilis SCDR1* (Table 3). Although chitosan nanosilver composites have documented combined effect against both Gram positive and negative pathogens [37] no effect was observed against *P. mirabilis SCDR1.* Silver is a highly toxic element for microbes. The Nanosilver exhibits high surface to volume ratio, which shows increased antimicrobial power in comparison to the same bulk silver material [55]. It is suggested that the antimicrobial mechanism of silver ions involves the disruption of phospholipids of cytoplasmic, and the disruption of DNA replication, impairing the function of ribosomes to transcribe messenger RNA and/or inactivation of cytochrome b by binding with sulfhydryl group [56]. *P. mirabilis SCDR1* genome analysis showed that our isolate contains a large number of genes (245) responsible for xenobiotics biodegradation and metabolism (Additional file 2: Table S2). Although *P. mirabilis SCDR1* does not contain the chitinase genes responsible for Chitin and chitosan degradation, it contained Chitin binding protein (cbp, 203 amino acid protein). This may justify the ability of *P. mirabilis* SCDR1 to resist the antimicrobial effect of chitosan. Chitin-binding protein even without any catalytic domain can facilitate the degradation of β-chitin by means of disrupting the crystalline chitin polymer structure [57, 58]. Microbial ability to produce proteins with high specific affinity to a certain crystalline chitin structure could be pivotal for the capability of bacteria to differentiate and react to specific crystalline chitin structures [59]. In addition, these chitin-binding domains may affect chitin degradation by facilitating adhesion of cells to the chitinous materials [57]. Thus, although we did not detect chitinase genes in *P. mirabilis SCDR1, the* presence of Chitin-binding protein suggests that *P. mirabilis SCDR1* has some mechanisms of protection against chitin and the chitosan antimicrobial effect. In addition, the presence of genes encoding for the members Chitosanase family GH3 of N, N′-diacetylchitobiose-specific 6-phospho-beta-glucosidase (EC 3.2.1.86), Beta N-acetyl-glucosaminidase (nagZ, beta-hexosaminidase) (EC 3.2.1.52), and Glucan endo-1, 4-beta-glucosidase (EC 3.2.1.-) in *P. mirabilis* SCDR1 suggests that it can hydrolyze chitosan to glucosamine [60–62]. This justifies the lack of antimicrobial effect of chitosan against *P. mirabilis SCDR1.* Likewise, *P. mirabilis* SCDR1 showed resistance against all the tested commercially available silver and Nanosilver containing wound dressing bandages. These silver containing commercially available bandages (wound dressing material) use different manufacturing technology and constituents. For example, Silvercel wound dressing contains high G calcium alginate in addition to 28% Silver-coated fibers (dressing contains 111 mg silver/100 cm^2^). The silver-coated fibers encompass elemental silver, which is converted to silver oxide upon contact with oxygen. Silver oxide dissolves in fluid and releases ionic silver (Ag^+^) that has antimicrobial action [63]. Clinical studies showed that Silvercel wound dressing is effective against many common wound pathogens, including methicillin-resistant Staphylococcus aureus (MRSA), methicillin -resistant Staphylococcus epidermidis (MRSE) and vancomycin-resistant Enterococcus (VRE). In addition, these studies showed that Silvercel wound dressing prevented and disrupted the formation of bacterial biofilms [64, 65]. However, this was not the case with our *P. mirabilis* SCDR1 isolate. Similarly, Sorbsan Silver wound dressing which is made of the fiber of the calcium salt of the alginic acid that contains 1.5% silver [66–68] did not show any antimicrobial effect against *P. mirabilis* SCDR1 isolate. Likewise, Colactive® Plus Ag, which is a silver impregnated collagen-based /alginate foam sheet wound dressing, did not show any antimicrobial effect against *P. mirabilis* SCDR1 isolate. In addition, Exsalt ®SD7 is a silver wound dressing that uses silver oxysalts technology. Silver oxysalts offer greater oxidation states of silver* (Ag^2+^, Ag^3+^) capable of interacting with microbial DNA, proteins and lipids, as well as providing potent oxidizing action through the increased power of Ag2^+,3+^ for advanced biocidal activity. Exsalt ®SD7 showed high antimicrobial activity against tested Gram-negative and positive bacteria and fungi tested [69]. *P. mirabilis* SCDR1 isolate showed high resistance against Exsalt ®SD7. In addition, *P. mirabilis* SCDR1 isolate showed high resistance against Puracol ® Plus Ag, which is made of 100% Collagens in addition to antimicrobial silver. Furthermore, Actisorb® Silver 220, which is a sterile primary dressing encompassing an activated charcoal cloth, impregnated with silver within a spun bonded perforated nylon sleeve [70] was not active against *P. mirabilis* SCDR1 isolate.

Pathogenomics analysis showed that *P. mirabilis* SCDR1 isolate is a potential virulent pathogen (Additional files 3 and 4: Tables 3 and 4). *P. mirabilis* SCDR1 shows that it possesses the characteristic bull’s eye pattern of swarming behavior. Presenting swarmer cells form is associated with the increase in expression of virulence genes [71]. Swarming is important to *P. mirabilis* uropathogenesis. It has been shown that swarming bacteria that move in multicellular groups exhibit adaptive resistance to multiple antibiotics [72]. Swarming behavior promotes the survival of bacteria in harsh environments or in unfavorable conditions. Moreover, migrating swarm cells display an increased resistance to many of antimicrobial agents. Therefore antimicrobial resistance could be a general feature of bacterial multicellular social behavior [73]. For example, the swarm cells of *P. aeruginosa* were able to migrate very close to the disc containing arsenite, indicating resistance to this heavy metal [73]. It has been suggested that high densities promote bacterial survival, the ability to move, as well as the speed of movement, confers an added advantage, making swarming an effective strategy for prevailing against antimicrobials including heavy metals [72, 73]. Furthermore, altruism or self-sacrifice is a suggested phenomenon associated with swarming, which involves risk of wiping out some individuals upon movement of bacteria to a different location, allowing the remaining individuals to continue their quest [72, 74]. Another suggested phenomenon associated with swarming is selfish behavior, in which the survival may be highest on top cells that are furthest from the antimicrobial agent while the lower cells in the swarm die because of the proximity to antimicrobial agents [72, 75]. Thus, selfish cells within the swarm sense where the best location is to avoid the toxic effect of the antimicrobial agent. Swarming behavior may indeed be one main reason for the observed nanosilver resistance of *P. mirabilis* SCDR1. Thus, maintaining high cell density, through the observed quorum sensing ability (Additional file 4: Table S4) and the circulation within the multilayered colony to minimize exposure to the heavy metal in addition to the death of individuals that are directly exposed, could be the key to the observed nanosilver resistance.

*P. mirabilis* SCDR1 isolate exhibited the ability of biofilm formation and also our pathogenomics analysis showed that it contains the genes responsible for this, such as glpC gene coding for anaerobic glycerol-3-phosphate dehydrogenase subunit C (EC 1.1.5.3), pmrI gene coding for UDP-glucuronic acid decarboxylase and baaS gene coding for biofilm formation regulatory protein BssS. We believe that the ability of *P. mirabilis* SCDR1 to form biofilm may also assist in the observed Nanosilver resistance. Biofilm formation can reduce the metal toxic effect by trapping it outside the cells. It was found that in the relative bacteria *Proteus vulgaris* XC 2, the biofilm cells showed considerably greater resistance to Chloromycetin compared to planktonic cells (free-floating counterparts) [76]. Moreover, it is suggested that the ability of biofilm formation may play a pivotal role in Polymyxin B antibiotic resistance in *P. mirabilis* [77]. Furthermore, it was found that biofilm formation is very important for heavy metal resistance in *Pseudomonas sp.* and that a biofilm lacking mutant was less tolerant to heavy metals [78]. Furthermore, it was found that both Extracellular Polysaccharides and Biofilm Formation is a resistance mechanism against toxic metals in *Sinorhizobium meliloti*, the nitrogen-fixing bacterium [79]. In addition, several reports claimed that the minimum inhibitory concentration (MIC) of some antibiotics for biofilms can be 1000-fold higher than that for planktonic bacteria [80].

It is well known that there are several mechanisms for metal resistance. These include physicochemical interactions, efflux, intracellular sequestration and extracellular precipitation by the excreted polymeric compounds [79]. Indeed, additional to swarming activity, Polysaccharides and biofilm formation (Additional file 4: Table S4), *P. mirabilis* SCDR1 contains several genes and proteins that also facilitate metal resistance including silver and Nanosilver (Table 8). Our results indicate the presence of endogenous silver and copper resistance mechanism in *P. mirabilis* SCDR1. We observed the presence of gene determinants of Copper/silver efflux system, oprB encoding for Copper/silver efflux system outer membrane protein CusC (outer membrane efflux protein OprB), oprM encoding for Copper/silver efflux system outer membrane protein CusC (outer membrane efflux protein OprM), cusC_1 encoding for Copper/silver efflux system outer membrane protein CusC (RND efflux system outer membrane lipoprotein), cpxA encoding for Copper sensory histidine kinase and outer membrane component of tripartite multidrug resistance system (CusC). In addition, we observed the presence of several Copper resistance genes/proteins were detected, namely, copA, copB, copC, copD, cueO, cueR, cutC, cutF and CuRO_2_CopA_like1. A similar endogenous silver and copper resistance mechanism has been described in *E. coli* and has been associated with the loss of porins from the outer membrane and up-regulation of the native Cus efflux mechanism, which is capable of transporting silver out of the cell [81, 82]. However, the genetic basis resistant phenotypes are still not fully known, and it is not known if they are obligatory or sufficient to exhibit resistance to silver [83]. Thus, we suggest a comprehensive study for this endogenous silver resistance mechanism within the *Proteus mirabilis* as well as *E. coli*.

Furthermore, we observed the presence of genes encoding to enzymes involved in heavy metal resistance such as Glutathione S-transferase (EC 2.5.1.18) (gst1, gst, Delta and Uncharacterized) in *P. mirabilis* SCDR1 genome. Thus, we propose a role of Glutathione S-transferases of *P. mirabilis* SCDR1 in the observed Nanosilver resistance. Glutathione S-transferases (GSTs) are a family of multifunctional proteins that play an important role in the detoxification of harmful physiological and xenobiotic compounds in organisms [84]. Moreover, it was found that a Glutathione S-transferase is involved in copper, cadmium, Lead and mercury resistance [85]. Furthermore, it was found that GST genes are differentially expressed in defense against oxidative stress caused by Cd and Nanosilver exposure [85].

Moreover, we observed the presence of a complete tellurite resistance operon (terB, terA, terC, terD, terE, terZ) which was suggested as contributing to virulence or fitness and protection from other forms of oxidative stress or agents causing membrane damage, such as silver and Nanosilver, in *P. mirabilis* [86]. Several other heavy metal resistance genes and proteins were observed in the *P. mirabilis* SCDR1 genome. These included arsM encoding for arsenite S-adenosylmethyltransferase (Methyltransferase type 11), which play an important role in prokaryotic resistance and detoxification mechanism to arsenite [87, 88] and merB encoding for alkylmercury lyase that cleaves the carbon-mercury bond of organomercurials, such as phenylmercuric acetate [89]. Moreover, numerous heavy metal resistance proteins were observed, such as magnesium/cobalt efflux protein CorC, metal resistance proteins, nickel-cobalt-cadmium resistance protein NccB, arsenical pump membrane protein (ArsB permease), Lead, cadmium, zinc and mercury transporting ATPase (Table 8).

In order to gain information about antimicrobial resistome constituents in *P. mirabilis* species, we performed comparative genomics analysis amongst all available 56 *P. mirabilis* genomes, including the *P. mirabilis* SCDR1 genome. As stated before, all *P. mirabilis* genomes shared 16 AMROs (Table 6). For example, all genomes contained the AMRO of copper sensory histidine kinase CpxA in cpxA mutant confer resistant to amikacin, copper-sensing two-component system response regulator CpxR, which is a regulator that promotes acrD expression when phosphorylated by a cascade involving CpxA, a sensor kinase and linked to cefepime and chloramphenicol resistance in *Klebsiella pneumoniae* [90]. However, different *P. mirabilis* genomes varied in the remaining 45 studied AMRO (Table 6). For example, genomics analysis of *P. mirabilis*-SCDR1 showed that our isolates contained genetic determinants for fluoroquinolones resistance (gyrA, parC and parE) [91, 92], Daptomycin and Rifamycin resistance (rpoB) [93], Chloramphenicol (cpxR, cpxA and cat) [90, 94], Ethidium bromide-methyl viologen resistance protein (emrE) [95] and Polymyxin and colistin resistance (phoP) [96]. In addition, several multidrug resistance efflux systems and complexes were observed. These include MdtABC-TolC, which is a multidrug efflux system in Gram-negative bacteria, including *E. coli* and *Salmonella* that confer resistance against β-lactams, novobiocin and deoxycholate. It is noteworthy that MdtABC-TolC and AcrD plays a role in metal resistance (copper and zinc), along with their BaeSR regulatory system [97] which was also was found in our *P. mirabilis* SCDR1 genome [Table 7], and thus may also play an additional role in silver resistance. MdtABC-TolC contains MdtA, which is a membrane fusion protein, TolC, which is the outer membrane channel and MdtBC that forms a drug transporter. In the absence of MdtB, the MdtAC-TolC has narrower drug specificity, leading to the loss of novobiocin resistance [98]. The MdtABC and AcrD systems may be related to bacterial metal homeostasis by transporting metals directly. This is to some extent similar to the copper and silver resistance mechanism by cation efflux of the CusABC system belonging to the RND protein superfamily [97, 99].

As stated before, this is the first report for spontaneous resistance against nanosilver. However, Gunawan et al., (2013) reported the natural ability of *Bacillus sp.* to adapt to nanosilver cytotoxicity under prolonged cellular oxidative stress stimulation through the nanoparticles incubation [14]. They found that the induced effects of adaptation continued even after discontinuation of nanosilver exposure. They also suggested that this characteristic ability of the ubiquitously-occurring *Bacillus sp.* may pose adversative consequences to the extensive use of nanosilver. Moreover, Graves et al., (2015) showed that after 225 generations of the treatment with nanosilver, the treated *E. coli* populations demonstrated greater fitness compared with control strains in the presence of silver nanoparticles [54]. We could also have suggested that the observed *P. mirabilis* SCDR1 nanosilver resistance might be an adaptive effect of the use of silver containing bandages in the Diabetic foot ulcer clinic. However, the reference strain *P. mirabilis* ATCC 29906 (isolated from urogenital tract of Homo sapiens) also showed resistance against colloidal nanosilver. In addition, it seems that nanosilver resistance is a widespread character in *P. mirabilis* species, since all other tested 50 isolates showed resistance against colloidal nanosilver [unpublished data]. Comparative metal resistance in *P. mirabilis* genomes contains some of the genetics determinants that can aid in nanosilver/silver resistance [unpublished data]. However, this conclusion needs to be further tested in a larger number of *P. mirabilis* isolated from different infection sites and geographical locations.

Increasing antimicrobial nanosilver usage could prompt a silver resistance problem in Gram-negative pathogens, particularly since silver resistance is already known to exist in several such species [81, 100]. Both exogenous (horizontally acquired Sil system) endogenous (mutational Cus system) resistance to silver has been reported in Gram-negative bacteria [13, 81]. Li et al. [81] selected five *Escherichia coli* mutants that present a ≥ 64-fold decreases in silver susceptibility compared with their original strain. All the mutants exhibited loss of expression of outer membrane porins (OmpF or OmpF/C), which seemingly resulted in the reduction of outer membrane permeability. These findings implied that reduced silver susceptibility is a result of restricting silver entrance into the bacterial cell. Moreover, they found that these mutants express active efflux that pumps silver outside of the cell. It was found that the *cus* CFBA operon is the responsible of silver efflux pump. Similarly, in our case, we observed the presence of resistance operon with high similarity to the *cus* operon, which is a chromosomally encoded system because of the lack of any plasmid in *P. mirabilis* SCDR1. However, both endogenous and exogenous silver resistance systems, in Gram-negative bacteria, remain incompletely understood [83].

The occurrence of induced nanosilver resistance (in vitro) in *Bacillus sp.* and *E. coli* [14, 54], spontaneous resistance (in our case) and the frequent uses and misuses of nanosilver-containing medical products should suggest adopting an enhanced surveillance systems for nanosilver-resistant isolates in medical setups. In addition, there should be greater control over utilizing nanosilver-containing products in order to maintain nanosilver as a valuable alternative approach in the fight against multidrug resistant pathogens.

## Conclusion

In the present study, we introduced the *P. mirabilis SCDR1* isolate that was collected from a diabetic ulcer patient. *P. mirabilis SCDR1* showed high levels of resistance against nanosilver colloids, nanosilver chitosan composite and the commercially available nanosilver and silver bandages. Our isolate contains all the required pathogenicity and virulence factors to establish a successful infection. *P. mirabilis SCDR1* contains several physical and biochemical mechanisms for antibiotics and silver/nanosilver resistance, which are biofilm formation, swarming mobility, efflux systems, and enzymatic detoxification.

## Additional files

Additional file 1: Table S1. Distribution of unique gene counts amongst different metabolic pathways. (DOCX 11 kb)

Additional file 2: Table S2. Distribution of unique gene counts amongst pathways Classes and subclasses. (DOCX 16 kb)

Additional file 3: Table S3. *P. mirabilis* SCDR1 Pathogen Finder results. (DOCX 25 kb)

Additional file 4: Table S4. Major pathogenic virulence factors for *Proteus mirabilis* SCDR1. (DOCX 32 kb)

Additional file 5: Table S5. Strict Antibiotic resistance analysis of Proteus mirabilis SCDR1. (DOCX 17 kb)

Additional file 6: Table S6. Modified loose Antibiotic resistance analysis of Proteus mirabilis SCDR1. (DOCX 65 kb)

Additional file 7: Table S7. Drug Resistance related protiens and its corresponding genes or proteins GenBank access numbers. (DOCX 16 kb)

## Abbreviations

16S rRNA: 16S ribosomal RNA gene
AMRO: Antimicrobial Resistance based ontology
AROs: Antibiotic Resistance Ontology
BLASTn: Basic Local Alignment Search Tool nucleotide
bp: Base pair
DDT: 1, 1, 1-Trichloro-2, 2-bis (4-chlorophenyl) ethane
DFU: Diabeticfoot ulcer
GC content: guanine-cytosine content
KFSHRC: King Faisal Specialist Hospital and Research Center
Mb: Mega base pairs
MDR: multidrug-resistant
MIC: Minimum Inhibitory Concentration
MRSA: methicillin-resistant Staphylococcus aureus
MRSE: methicillin -resistant Staphylococcus epidermidis
NGS: Next generation sequencing techniques
PATRIC: Pathosystems recourse Integration center
PPM: part per million
RGI: Resistance Gene Identifier
RND: Resistance-Nodulation- Division
SCDR: Strategic center for Diabetes research
tRNAs: Transfer ribonucleic acid
VRE: Vancomycin-resistant Enterococcus
