# Supplementary material for: Genome sequencing and analysis of the first spontaneous Nanosilver resistant bacterium Proteus mirabilis strain SCDR1
Source: Antimicrob Resist Infect Control. 2017 Nov 23;6:119. doi: 10.1186/s13756-017-0277-x (PMC5701452; doi:10.1186/s13756-017-0277-x)
Supplement: Supplementary file 1 — Distribution of unique gene counts amongst different metabolic pathways. (DOCX 11 kb) [file 13756_2017_277_MOESM1_ESM.docx]

| **Pathway Class** | **Unique Gene Count** |
| --- | --- |
| Amino Acid Metabolism | 397 |
| Biosynthesis of Polyketides and Nonribosomal Peptides | 85 |
| Biosynthesis of Secondary Metabolites | 308 |
| Carbohydrate Metabolism | 477 |
| Energy Metabolism | 218 |
| Glycan Biosynthesis and Metabolism | 137 |
| Lipid Metabolism | 185 |
| Metabolism of Other Amino Acids | 91 |
| Metabolism of Cofactors and Vitamins | 227 |
| Nucleotide Metabolism | 176 |
| Signal Transduction | 5 |
| Translation | 34 |
| Xenobiotics Biodegradation and Metabolism | 245 |

Table S1: Distribution of unique gene counts amongst different metabolic pathways.
