# Supplementary material for: Genome sequencing and analysis of the first spontaneous Nanosilver resistant bacterium Proteus mirabilis strain SCDR1
Source: Antimicrob Resist Infect Control. 2017 Nov 23;6:119. doi: 10.1186/s13756-017-0277-x (PMC5701452; doi:10.1186/s13756-017-0277-x)
Supplement: Supplementary file 2 — Distribution of unique gene counts amongst pathways Classes and subclasses. (DOCX 16 kb) [file 13756_2017_277_MOESM2_ESM.docx]

Table S2: Distribution of unique gene counts amongst pathways Classes and subclasses.

| **Pathway Class/Name** | **Unique Gene Count** |
| --- | --- |
| **Energy Metabolism** | |
| Oxidative phosphorylation | 30.0 |
| Photosynthesis | 9.0 |
| Sulfur metabolism | 17.0 |
| Carbon fixation in photosynthetic organisms | 23.0 |
| Nitrogen metabolism | 52.0 |
| Reductive carboxylate cycle (CO2 fixation) | 33.0 |
| Methane metabolism | 54.0 |
| **Biosynthesis of secondary Metabolites** | |
| Tetracycline biosynthesis | 24.0 |
| Zeatin biosynthesis | 10.0 |
| Streptomycin biosynthesis | 7.0 |
| Puromycin biosynthesis | 4.0 |
| Stilbenoid, diarylheptanoid and gingerol biosynthesis | 26.0 |
| Flavone and flavonol biosynthesis | 8.0 |
| Isoflavonoid biosynthesis | 3.0 |
| Anthocyanin biosynthesis | 23.0 |
| Flavonoid biosynthesis | 15.0 |
| Phenylpropanoid biosynthesis | 11.0 |
| Novobiocin biosynthesis | 8.0 |
| Tropane, piperidine and pyridine alkaloid biosynthesis | 20.0 |
| Betalain biosynthesis | 31.0 |
| Isoquinoline alkaloid biosynthesis | 32.0 |
| Insect hormone biosynthesis | 12.0 |
| Terpenoid backbone biosynthesis | 16.0 |
| Limonene and pinene degradation | 19.0 |
| Diterpenoid biosynthesis | 14.0 |
| Brassinosteroid biosynthesis | 3.0 |
| Carotenoid biosynthesis | 22.0 |
| **Lipid Metabolism** | |
| Steroid biosynthesis | 1.0 |
| Fatty acid elongation in mitochondria | 4.0 |
| Fatty acid biosynthesis | 32.0 |
| Secondary bile acid biosynthesis | 2.0 |
| Primary bile acid biosynthesis | 4.0 |
| Biosynthesis of unsaturated fatty acids | 1.0 |
| C21-Steroid hormone metabolism | 6.0 |
| Sphingo Lipid Metabolism | 16.0 |
| Glycero Lipid Metabolism | 21.0 |
| Glycerophospho Lipid Metabolism | 40.0 |
| Ether Lipid Metabolism | 20.0 |
| Fatty acid metabolism | 18.0 |
| Linoleic acid metabolism | 7.0 |
| Arachidonic acid metabolism | 3.0 |
| alpha-Linolenic acid metabolism | 10.0 |
| **Amino Acid Metabolism** | |
| Lysine biosynthesis | 22.0 |
| Tryptophan metabolism | 19.0 |
| Alanine, aspartate and glutamate metabolism | 30.0 |
| Glycine, serine and threonine metabolism | 46.0 |
| Phenylalanine metabolism | 35.0 |
| Phenylalanine, tyrosine and tryptophan biosynthesis | 30.0 |
| Tyrosine metabolism | 31.0 |
| Histidine metabolism | 29.0 |
| Valine, leucine and isoleucine biosynthesis | 16.0 |
| Arginine and proline metabolism | 63.0 |
| Cysteine and methionine metabolism | 38.0 |
| Valine, leucine and isoleucine degradation | 11.0 |
| Lysine degradation | 27.0 |
| **Metabolism of Other Amino Acids** | |
| Glutathione metabolism | 35.0 |
| beta-Alanine metabolism | 8.0 |
| D-Alanine metabolism | 5.0 |
| D-Arginine and D-ornithine metabolism | 4.0 |
| D-Glutamine and D-glutamate metabolism | 4.0 |
| Seleno Amino Acid Metabolism | 20.0 |
| Cyano Amino Acid Metabolism | 7.0 |
| Taurine and hypotaurine metabolism | 5.0 |
| Phosphonate and phosphinate metabolism | 3 |
| **Glycan Biosynthesis and Metabolism** | |
| O-Glycan biosynthesis | 8.0 |
| High-mannose type N-glycan biosynthesis | 11.0 |
| Glycosaminoglycan degradation | 7.0 |
| Glycosphingolipid biosynthesis - ganglio series | 21.0 |
| Glycosphingolipid biosynthesis - globo series | 9.0 |
| Glycosphingolipid biosynthesis - lacto and neolacto series | 9.0 |
| Lipopolysaccharide biosynthesis | 32.0 |
| Peptidoglycan biosynthesis | 31.0 |
| Glycosylphosphatidylinositol(GPI)-anchor biosynthesis | 9.0 |
| **Metabolism of Cofactors and Vitamins** | |
| Folate biosynthesis | 22.0 |
| Thiamine metabolism | 17.0 |
| Ubiquinone and other terpenoid-quinone biosynthesis | 31.0 |
| One carbon pool by folate | 19.0 |
| Riboflavin metabolism | 13.0 |
| Vitamin B6 metabolism | 9.0 |
| Porphyrin and chlorophyll metabolism | 33.0 |
| Nicotinate and nicotinamide metabolism | 19.0 |
| Pantothenate and CoA biosynthesis | 25.0 |
| Lipoic acid metabolism | 2.0 |
| Retinol metabolism | 10.0 |
| Biotin metabolism | 27.0 |
| **Xenobiotics Biodegradation and Metabolism** | |
| Atrazine degradation | 5.0 |
| 1,4-Dichlorobenzene degradation | 27.0 |
| Naphthalene and anthracene degradation | 23.0 |
| Tetrachloroethene degradation | 16.0 |
| 1- and 2-Methylnaphthalene degradation | 19.0 |
| 2,4-Dichlorobenzoate degradation | 19.0 |
| Toluene and xylene degradation | 14.0 |
| Biphenyl degradation | 6.0 |
| Bisphenol A degradation | 13.0 |
| Benzoate degradation via hydroxylation | 33.0 |
| gamma-Hexachlorocyclohexane degradation | 4.0 |
| Caprolactam degradation | 3.0 |
| 1,1,1-Trichloro-2,2-bis(4-chlorophenyl)ethane (DDT) degradation | 5.0 |
| Drug metabolism - other enzymes | 11.0 |
| Trinitrotoluene degradation | 11.0 |
| Drug metabolism - cytochrome P450 | 7.0 |
| Metabolism of xenobiotics by cytochrome P450 | 7.0 |
| Ethylbenzene degradation | 13.0 |
| Styrene degradation | 4.0 |
| Geraniol degradation | 5.0 |
| **Signal Transduction** | |
| mTOR signaling pathway | 1.0 |
| Phosphatidylinositol signaling system | 4.0 |
| **Nucleotide Metabolism** | |
| Purine metabolism | 106.0 |
| Pyrimidine metabolism | 70.0 |
| **Translation** | |
| Aminoacyl-tRNA biosynthesis | 34.0 |
