# Supplementary material for: Genome sequencing and analysis of the first spontaneous Nanosilver resistant bacterium Proteus mirabilis strain SCDR1
Source: Antimicrob Resist Infect Control. 2017 Nov 23;6:119. doi: 10.1186/s13756-017-0277-x (PMC5701452; doi:10.1186/s13756-017-0277-x)
Supplement: Supplementary file 3 — P. mirabilis SCDR1 Pathogen Finder results. (DOCX 25 kb) [file 13756_2017_277_MOESM3_ESM.docx]

Table S3: *P. mirabilis* SCDR1 Pathogen Finder results.

| **Etiology** | **Reference Genome** | **Accession Number** | **Conserved Protein Domain(s) Family** | **Protein ID** | **AA Length** | **Corresponding Protein** |
| --- | --- | --- | --- | --- | --- | --- |
| Human Pathogenic | *P. mirabilis* HI4320 | NC_010554 | Peptidase M16 | WP_060557795.1 | 931 aa | peptidase M16 |
| Human Pathogenic | *P. mirabilis* ATCC 29906 | NZ_GG668580 | YtcJ_like metal dependent amidohydrolases | WP_060557038.1 | 586 aa | amidohydrolase family protein |
| Human Pathogenic | *P. mirabilis* HI4320 | NC_010554 | ABC transporter transmembrane region.  P-loop_NTPase. | WP_060557796.1 | 569 aa | hypothetical protein |
| Human Pathogenic | *P. mirabilis* HI4320 | NC_010554 | GadA: Glutamate or tyrosine decarboxylase or a related PLP-dependent protein. | WP_060557561.1 | 578 aa | cytochrome D ubiquinol oxidase subunit II |
| Human Pathogenic | *P. mirabilis* HI4320 | NC_010554 | OEP | CAR42692.1 | 490 aa | channel-forming component of a multidrug resistance efflux pump |
| Human Pathogenic | *P. mirabilis* HI4320 | NC_010554 | HATPase_c: Histidine kinase-like ATPases.  HisKA. | WP_060558524.1 | 467 aa | Swarming motility regulation sensor protein RssA |
| Human Pathogenic | *P. mirabilis* HI4320 | NC_010554 | Branch_AA_trans | WP_060558661.1 | 450 aa | branched-chain amino acid transporter |
| Human Pathogenic | *P. mirabilis* HI4320 | NC_010554 | MFS: The Major Facilitator Superfamily (MFS) is a large and diverse group of secondary transporters that includes uniporters, symporters, and antiporters | WP_017827553 | 445 aa | MFS transporter |
| Human Pathogenic | *P. mirabilis* HI4320 | NC_010554 | Baseplate_J: Baseplate J-like protein | WP_060558503 | 395 aa | hypothetical protein |
| Human Pathogenic | *P. mirabilis* HI4320 | NC_010554 | AAT_like: Aspartate aminotransferase family | WP_060559082 | 389 aa | aminotransferase |
| Human Pathogenic | *P. mirabilis* HI4320 | NC_010554 | DNA_processg_A | WP_060558450 | 385 aa | DNA protecting protein DprA |
| Human Pathogenic | *P. mirabilis* HI4320 | NC_010554 | PRK14992: tetrathionate reductase subunit C | WP_060558547 | 342 aa | tetrathionate reductase subunit C |
| Human Pathogenic | *P. mirabilis* HI4320 | NC_010554 | ATPgrasp_TupA | WP_049196662 | 297 aa | glycosyl transferase |
| Human Pathogenic | *P. mirabilis* HI4320 | NC_010554 | PRK09709 | WP_049216798 | 272 aa | exodeoxyribonuclease VIII |
| Human Pathogenic | *P. mirabilis* HI4320 | NC_010554 | Methyltransf_6: Demethylmenaquinone methyltransferase | WP_060557630 | 232 aa | dimethylmenaquinone methyltransferase |
| Human Pathogenic | *P. mirabilis* HI4320 | NC_010554 | SPOR | WP_060558032 | 212 aa | cell division protein DedD |
| Human Pathogenic | *P. mirabilis* HI4320 | NC_010554 | FabA: 3-hydroxymyristoyl/3-hydroxydecanoyl-(acyl carrier protein) dehydratase | WP_060556751 | 192 aa | beta-hydroxyacyl-ACP dehydratase |
| Human Pathogenic | *E. coli* | JPXM01000121.1 | P-loop_NTPase: P-loop containing Nucleoside Triphosphate Hydrolases | KGT24373.1 | 82 aa | Elongation factor Tu |
| Human Pathogenic | *P. mirabilis* ATCC 29906 | ACLE01000003.1 | CpxP_like: CpxP component of the bacterial Cpx-two-component system and related proteins | EEI50011 | 193 aa | putative periplasmic stress adaptor protein CpxP |
| Human Pathogenic | *P. mirabilis* HI4320 | NC_010554 | FimA: Pilin (type 1 fimbria component protein) | WP_004244332 | 183 aa | fimbrial subunit |
| Human Pathogenic | *P. mirabilis* HI4320 | NC_010554 | FimA: Pilin (type 1 fimbria component protein) | WP_004246312.1 | 173 aa | fimbrial protein |
| Human Pathogenic | *P. mirabilis* HI4320 | NC_010554 | Acetyltransf_9 | WP_049201900.1 | 169 aa | acetyltransferase |
| Human Pathogenic | *P. mirabilis* HI4320 | NC_010554 | alpha-crystallin-Hsps_p23-like: alpha-crystallin domain (ACD) found in alpha-crystallin-type small heat shock proteins, and a similar domain found in p23 (a cochaperone for Hsp90) and in other p23-like proteins. | WP_060559475.1 | 152 aa | heat-shock protein |
| Human Pathogenic | *P. mirabilis* HI4320 | NC_010554 | PRK06602: NADH:ubiquinone oxidoreductase subunit A | WP_004243698.1 | 150 aa | NADH-quinone oxidoreductase subunit A |
| Human Pathogenic | *P. mirabilis* HI4320 | NC_010554 | HicB_like antitoxin of bacterial toxin-antitoxin system | WP_004247923.1 | 143 aa | hypothetical protein (Antitoxin HicB like) |
| Human Pathogenic | *P. mirabilis* HI4320 | NC_010554 | DUF3749 (NAT_SF) | WP_060557743.1 | 138 aa | acetyltransferase |
| Human Pathogenic | *P. mirabilis* HI4320 | NC_010554 | Phage_antitermQ | WP_004247847.1 | 132 aa | antitermination protein Q |
| Human Pathogenic | *P. mirabilis* HI4320 | NC_010554 | NA | WP_004248702.1 | 132 aa | hypothetical protein |
| Human Pathogenic | *P. mirabilis* HI4320 | NC_010554 | DUF2628 | WP_004242555.1 | 131 aa | membrane protein |
| Human Pathogenic | *P. mirabilis* HI4320 | NC_010554 | PRK11476: DNA-binding transcriptional activator CaiF | WP_060556315.1 | 130 aa | transcriptional regulator |
| Human Pathogenic | *P. mirabilis* HI4320 | NC_010554 | DAGK_IM | WP_049202973.1 | 123 aa | diacylglycerol kinase |
| Human Pathogenic | *P. mirabilis* HI4320 | NC_010554 | AzlD | WP_046334606.1 | 109 aa | branched-chain amino acid transport |
| Human Pathogenic | *P. mirabilis* HI4320 | NC_010554 | CutA1 | WP_060558754.1 | 102 aa | cation tolerance protein CutA |
| Not Pathogenic | *E. tasmaniensis* strain ET1/99 | CU468135.1 | ihfA | CAO96881.1 | 99aa | integration host factor subunit alpha |
| Human Pathogenic | *P. mirabilis* HI4320 | NC_010554 | PRK10497 | WP_004243098.1 | 85 aa | peptide permease |
| Human Pathogenic | *P. mirabilis* HI4320 | NC_010554 | NA | WP_004249586.1 | 69 aa | hypothetical protein |

**Annotation:** Ref Seq and PathogenFinder-1.1

**NA**: Not available

CLASS: Gammaproteobacteria
