# Supplementary material for: Genome sequencing and analysis of the first spontaneous Nanosilver resistant bacterium Proteus mirabilis strain SCDR1
Source: Antimicrob Resist Infect Control. 2017 Nov 23;6:119. doi: 10.1186/s13756-017-0277-x (PMC5701452; doi:10.1186/s13756-017-0277-x)
Supplement: Supplementary file 4 — Major pathogenic virulence factors for Proteus mirabilis SCDR1. (DOCX 32 kb) [file 13756_2017_277_MOESM4_ESM.docx]

| Table S4. Major pathogenic virulence factors for *Proteus mirabilis* SCDR1. | |
| --- | --- |
| **Swarming behavior, mobility and adherence** | |
| **GenBank Reference sequence/Accession Number** | **Corresponding Protein** |
| WP_020945900.1 | Swarming motility regulation two-component system, response regulator |
| WP_020945901.1 | Swarming motility regulation two-component system, sensor kinase |
| WP_004245210.1 | Transcriptional activator NhaR |
| WP_020945892.1 | Colanic acid capsular biosynthesis activation protein |
| AF071215.1 | *Proteus mirabilis* regulator of swarming behavior precursor (rsbA) and RcsB (rcsB) genes, complete cds; and RcsC (rcsC) gene, partial cds |
| AJ250100.1 | *Proteus mirabilis* mutT gene for putative 8-oxo-dGTPase and hpmBA gene promoter region (swarming coupled hemolysin operon) |
| AJ000084.1 | *Proteus mirabilis* ccm and pat genes and partial ygbA gene |
| AY298901.1 | *Proteus mirabilis* SpeB gene, complete cds |
| Z32686.2 | *Proteus mirabilis* fimbrial operon, strain HI4320 |
| Z35428.1 | *Proteus mirabilis* (HI4320) PMF fimbrial operon DNA |
| Z78535.1 | *Proteus mirabilis* atf gene cluster (ambient-temperature fimbriae) |
| Z18753.1 | *Proteus mirabilis* mrpA gene encoding MR/P major fimbrial subunit polypeptide |
| WP_004248675.1 | Fimbrial outer membrane usher protein |
| WP_023843636.1 | Fimbrial operon regulator |
| WP_023843576.1 | Fimbrial subunit |
| WP_023843515.1  WP_020945751.1  WP_020946328.1  WP_012368284.1  AGS58926.1  KKC60818.1 | Fimbrial adhesin |
| WP_020945364.1 | Adhesin secretion/activation protein |
| WP_020945365.1  WP_020946124.1 | Adhesin (haemagglutination) |
| WP_023843514.1 | Fimbrial protein |
| WP_020946325.1 | Type 1 fimbrial chaperone protein |
| WP_004245878.1 | Major subunit of type 1 fimbria |
| WP_004250164.1 | Fimbrial chaperone |
| WP_020946144.1 | Fimbrial outer membrane usher protein |
| WP_020945958.1 | Minor fimbrial subunit |
| WP_004243875.1 | Major fimbrial subunit |
| WP_004247325.1 | Major mannose-Resistant/Proteus-like fimbrial protein |
| WP_004247323.1 | Fimbriae recombinase |
| WP_020946460.1 | Fimbrial assembly protein |
| WP_004245452.1 | Lipoprotein involved with copper homeostasis and adhesion |
| WP_023843522.1 | Type IV prepilin-like leader peptidase |
| AF221596.1 | *Proteus mirabilis* FlaD (flaD) gene, partial cds; FlaA (flaA), FlaB (flaB), FliA (fliA), and FliZ (fliZ) genes, complete cds; and PutA (putA) gene, partial cds |
| U82214 | *Proteus mirabilis* flagella rod protein FlgD (flgD) gene, partial cds, flagella rod proteins FlgC (flgC) and FlgB (flgB), flagella assembly protein FlgA (flgA), anti-sigma factor FlgM (flgM), facilitator of flagella filament assembly FlgN (flgN) and FloA (floA) genes, complete cds |
| U66822.1 | *Proteus mirabilis* YrfE (yrfE) gene, partial cds; and UmoB (umoB) gene, complete cds |
| L36848.1 | *Proteus mirabilis* (clone LDII 73) flhB gene, 3' end, flhA gene, complete cds |
| U96964.1 | *Proteus mirabilis* flagella master operon flhDC: flagella class I protein FlhD (flhD) and flagella class I protein FlhC (flhC) genes, complete cds |
| WP_004243547.1 | Flagellar biosynthesis protein FlhB |
| WP_020946458.1 | Flagellar operon control protein (membrane protein) |
| WP_004246526.1 | Upregulator of flagellar operon (exported protein) |
| WP_020945522.1 | Upregulator of flagellar master operon |
| WP_004246526.1 | Upregulator of flagellar operon |
| WP_004246291.1 | Protein Hfq (host factor-I protein) |
| **Toxins and hemolysin** | |
| **GenBank Reference sequence/Accession Number** | **Corresponding Protein** |
| WP_020946383.1 | hemagglutinin |
| WP_020946384.1 | hemagglutinin |
| WP_020946416.1 | hemagglutinin |
| WP_023843509.1 | hemolysin expression-modulating protein |
| WP_012367444.1 | cell killing protein |
| WP_012368231.1 | hemolysin activator protein |
| WP_020946017.1 | hemolysin activator protein (two-partner secretion system accessory protein) |
| WP_020946018.1 | hemolysin |
| WP_023843509.1 | hemolysin expression-modulating protein |
| WP_012368231.1 | hemolysin activator protein |
| WP_020945364.1 | Hemolysin secretion/activation protein ShlB/FhaC/HecB |
| WP_004246291.1 | protein Hfq (host factor-I protein) |
| M30186.1 | *Proteus mirabilis* hemolysin (HpmA) and HpmA activator protein (HpmB) genes, complete cds |
| AJ250100.1 | *Proteus mirabilis* mutT gene for putative 8-oxo-dGTPase and hpmBA gene promoter region (swarming coupled hemolysin operon)* |
| CAR44626.1 | Auto-transporter (serine protease) (agglutinin pta) |
| AF064762.1 | *Proteus mirabilis* metalloprotease operon, complete sequence |
| HM217133.1 | *Proteus mirabilis* strain Pm7 metalloprotease zapA (zapA) gene, complete cds |
| U25950.1 | *Proteus mirabilis* metalloprotease gene, complete cds |
| **Urease** | |
| **GenBank Reference sequence/Accession Number** | **Corresponding Protein** |
| M31834.1 | *Proteus mirabilis* urease operon: ureA-ureF genes, complete cds |
| WP_004248932.1 | Urease operon transcriptional activator |
| WP_023843496.1 | Urease accessory protein |
| WP_004245265.1 | Urease gamma subunit |
| WP_004245263.1 | Urease beta subunit |
| WP_020945159.1 | Urease subunit alpha |
| WP_004245261.1 | Urease accessory protein UreE |
| WP_004245259.1 | Urease accessory protein UreG |
| WP_012368846.1 | AraC family/Urease operon transcriptional regulator/activator |
| WP_012368848.1 | Urease accessory protein UreF* |
| **Quorum sensing** | |
| **GenBank Reference sequence/Accession Number** | **Corresponding Protein** |
| AY044337.1 | Proteus mirabilis gamma-glutamylcysteine synthetase-like protein (gshI) gene, partial cds; autoinducer 2 synthetase-like protein LuxS (luxS) gene, complete cds; and unknown genes |
| **Iron acquisition (Heme uptake)** | |
| **GenBank Reference sequence/Accession Number** | **Corresponding Protein** |
| U22969.1 | *Proteus mirabilis* uroporphyrinogen III methylase (hemX) gene, partial cds, uroporphyrinogen III synthase (hemD), porphobilinogen deaminase (hemC), and adenylate cyclase (cya) genes, complete cds |
| WP_020945727.1  WP_020945728.1 | Hemin receptor |
| WP_004248182.1 | Hemin transport protein |
| AGS59947.1 | Hemin-binding periplasmic protein |
| WP_004248185.1 | Hemin transport system permease protein HmuU |
| WP_004243181.1 | Hemin importer ATP-binding subunit |
| WP_020945168.1 | TonB-dependent receptor |
| WP_012367583.1 | TonB-dependent receptor |
| WP_020945833.1 | TonB-dependent receptor |
| **Iron acquisition (Ferrous uptake)** | |
| WP_020945545.1  WP_004242585.1 | Iron ABC transporter membrane protein |
| WP_004251467.1 | Iron ABC transporter, ATP-binding protein |
| WP_004242594.1 | Iron ABC transporter, periplasmic substrate-binding protein |
| WP_023843630.1 | Ferrous iron transport protein A |
| WP_020946418.1 | Ferrous iron transport protein B |
| **Iron acquisition (Ferric uptake** **)** | |
| WP_012368029.1 | TonB-dependent receptor |
| WP_004247279.1 | Ferric ABC transporter, iron-binding protein |
| WP_020945166.1 | Putative exported protease |
| WP_004248284.1 | Exported protease |
| WP_020945169.1 | FecR-like transcriptional regulator |
| WP_004244382.1 | Ferric uptake regulator |
| WP_004248652.1  AGS60778.1 | Ferric enterobactin transport protein FepE |
| **Iron acquisition (Siderophores biosynthesis, ABC transport system and Ferri-siderophore transporters)** | |
| AGS58865.1 | Ferric ABC transporter, permease protein |
| WP_020945252.1, WP_004244479.1 | ABC transporter permease |
| WP_004245037.1 | ABC transporter, ATP-binding protein |
| WP_012368450.1 | MFS family transporter |
| WP_020945254.1 | Citrate lyase beta chain |
| WP_012367547.1  WP_020946433.1  WP_012367547.1 | Lysine/Ornithine decarboxylase |
| AGS58902.1,  WP_020945639.1  WP_004242906.1 | Pyridoxal-phosphate dependent enzyme |
| WP_012367924.1 | MFS family transporter |
| WP_012368605.1 | MFS family transporter |
| WP_020945255.1 | Siderophore biosynthesis protein |
| WP_020945256.1 | TonB-dependent siderophore receptor |
| WP_020945258.1 | Octopine/opine/tauropine dehydrogenase |
| WP_020945722.1 | Thioesterase |
| WP_012368139.1 | 4'-phosphopantetheinyl transferase (holo-ACP synthase) |
| WP_004243887.1 | 4'-phosphopantetheinyl transferase (holo-ACP synthase) |
| WP_020946292.1 | Iron compound ABC transporter, permease |
| WP_020946291.1 | Iron compound ABC transporter, substrate-binding protein |
| WP_020946293.1 | Iron compound ABC transporter, ATP-binding protein |
| WP_020945311.1 | TonB-dependent ferric siderephore receptor |
| WP_004246968.1  ALE20884.1  WP_004246967.1 | Bacterioferritin-associated ferredoxin |
| **Immune evasion, cell invasion and biofilm formation** | |
| **GenBank Reference sequence/Accession Number** | **Corresponding Protein** |
| WP_012367537.1 | Metalloprotease (ZAP E) |
| WP_004247335.1 | Serralysin (Metalloprotease) |
| WP_020945278.1 | Metalloprotease |
| WP_020945279.1 | Metalloprotease |
| WP_020945280.1 | Metalloprotease |
| WP_012367535.1 | Metalloprotease (ZAP E) |
| WP_012367538.1 | Metalloprotease |
| WP_012367537.1 | Metalloprotease |
| GQ457568.1 | Proteus mirabilis strain PmrIp UDP-glucuronic acid decarboxylase gene, complete cds |
| WP_020945551.1 | Bifunctional UDP-glucuronic acid decarboxylase/UDP-4-amino-4-deoxy-L-arabinose formyltransferase |
| CAR47032.1 | anaerobic glycerol-3-phosphate dehydrogenase subunit C (EC 1.1.5.3) |
| WP_004248355.1 | Biofilm formation regulatory protein BssS |
| WP_004243437.1*** | Polyphosphate kinase |
| **Stress tolerance** | |
| **GenBank Reference sequence/Accession Number** | **Corresponding Protein** |
| WP_004243889.1 | DNA repair protein RecO |
| WP_020945960.1 | DNA repair protein RecN |
| WP_004248572.1 | DNA mismatch repair protein MutH |
| WP_023843609.1 | DNA mismatch repair protein MutS |
| WP_004249295.1 | DNA repair protein RadA |
| WP_004249942.1 | DNA repair protein |
| WP_020946614.1 | DNA mismatch repair protein MutL |
| WP_012367603.1 | DNA recombination protein RecT |
| WP_004243604.1 | HdeD family acid resistance membrane protein |
| WP_004243437.1 | Polyphosphate kinase |
| WP_004246291.1 | Protein Hfq (host factor-I protein) |
| D50830.1 | Proteus mirabilis rpoH gene for sigma-32 homolog, complete cds |
| AJ400964.1 | Proteus mirabilis ORF1 DNA, katA gene and menE gene, wild type |
| WP_004242597.1 | Stress protection protein MarC (Intracellular trafficking and secretion) |
| WP_004243899.1 | RNA polymerase sigma factor RpoE |
| WP_004244532.1 | Glutathione S-transferase |
| WP_017628093.1 | copper-zinc superoxide dismutase |
| D50830.1 | Proteus mirabilis rpoH gene for sigma-32 homolog, complete cds |
| WP_004242516.1  WP_004243909.1 | Heat-shock protein |
| WP_004246651.1 | Heat shock protein 15 |
| WP_020945178.1 | Chaperone protein DnaK (heat shock protein 70) |
| AGS60781.1 | Heat shock protein 90 |
| AGS58848.1 | Heat shock protein HtpX |
| **Additional pathogenic virulence factors** | |
| **GenBank Reference sequence/Accession Number** | **Corresponding Protein** |
| WP_004243899.1 | RNA polymerase sigma factor RpoE |
| WP_020946298.1, WP_020946303.1  WP_004245838.1, AGS61148.1  WP_020946308.1, AGS61150.1  WP_004245842.1, WP_020946312.1  AGS61155.1 | Type III secretion system protein |
| AGS61156.1, AGS61157.1 | Type III secretion system protein (oxygen-regulated invasion protein) |
| WP_004245838.1 | Invas_SpaK |
| WP_020946308.1 | Type III secretion system regulator InvE |
| WP_020946308.1 | HrpJ |
| AGS61150.1 | type_III_yscC |
| AGS61150.1 | Secretin N |
| AGS61150.1 | Secretin |
| WP_020946311.1 | MxiH |
| WP_020946312.1 | PRK15325 (PrgJ) |
| AGS61155.1 | YscJ_FliF |
| AGS61156.1 | OrgA_MxiK |
| WP_020946300.1 | EscU/YscU/HrcU family type III secretion system export apparatus switch protein |
| WP_020946301.1 | EscT/YscT/HrcT family type III secretion system export apparatus protein |
| WP_004245832.1 | EscS/YscS/HrcS family type III secretion system export apparatus |
| WP_020946307.1 | EscV/YscV/HrcV family type III secretion system export apparatus protein |
| WP_020946309.1 | EscC/YscC/HrcC family type III secretion system outer membrane ring protein |
| WP_020946311.1 | EscF/YscF/HrpA family type III secretion system needle major subunit |
| WP_020946313.1 | EscJ/YscJ/HrcJ family type III secretion inner membrane ring protein |
| AGS58943.1 | type I secretion outer membrane protein |
| AGS58943.1 | Type_I_sec_TolC |
| AGS58943.1 | OEP |
| WP_020945276.1 | HlyD family type I secretion periplasmic adaptor subunit |
| WP_020945276.1 | Type_I_hlyD |
| WP_020945276.1 | Biotin_lipoyl_2 |
| WP_020945276.1 | HlyD_3 |
| WP_020945277.1 | type I secretion ATP-binding protein |
| WP_020945277.1 | ArpD |
| WP_020945277.1 | ABC_membrane |
| WP_020945277.1 | P-loop_NTPase |
| WP_020945291.1 | HlyD family type I secretion periplasmic adaptor subunit |
| WP_020945291.1 | Type_I_hlyD |
| WP_020945291.1 | Biotin_lipoyl_2 |
| WP_020945291.1 | HlyD_3 |
| AGS58984.1 | Type II/IV secretion system protein |
| AGS58984.1 | P-loop_NTPase |
| WP_020945888.1 | Chemotaxis protein CheA (HPT, CheY-binding, H-kinase_dim, HATPase_c, CheA_reg) |
| AF064762.1 | Metalloprotease operon |
| HM217133.1 | Metalloprotease zapA |
| U25950.1 | Metalloprotease gene |
| HM146786.1, HM146785.1 | Lipopolysaccharide biosynthesis genes  HldD (hldD) gene, partial cds; and WaaF (waaF), WaaC (waaC), WamD (wamD), Mig-14 (mig-14), WabN (wabN), WabH (wabH), WabG (wabG), WaaQ (waaQ), WaaA (waaA), WaaE (waaE), CoaD (coaD), Fpg (fpg), WaaL (waaL), WalM (walM), WalN (walN), WalO (walO), WalR (walR), RpmG (rpmG), RpmB (rpmB), and RadC (radC) genes, complete cds. |
| AY075039.1 | The inner-core lipopolysaccharide biosynthetic waaE gene: 3-deoxy-manno-octulosonic acid transferase (waaA) gene, partial cds; glucosyl-transferase (waaE) and phosphopantetheine denyltransferase (coaD) genes, complete cds; and Fpg (fpg) gene, partial cds. |
| KGA90873.1 | Chorismate synthase |
| AGS61469.1 | NADP-specific glutamate dehydrogenase |
| AGS61340.1 | Glutamine synthetase type I |
| AGS61311.1 | Dipeptide-binding ABC transporter, periplasmic substrate-binding component |
| AGS59997.1 | Oligopeptide ABC transporter, periplasmic oligopeptide-binding protein OppA |
| **Phage associated proteins, integrons and transposons elements.** | |
| **GenBank Reference sequence/**  **Accession Number** | **Corresponding Protein** |
| WP_020945485.1, WP_020945486.1  WP_004250586.1, WP_004250588.1  WP_020945487.1, WP_020945488.1  WP_020945489.1, WP_020945490.1  WP_020945491.1, WP_020945492.1  WP_020945493.1, WP_020945494.1  WP_012368084.1, WP_004247851.1  WP_012368092.1, WP_020945912.1 | Phage proteins |
| WP_004242548.1, WP_020945914.1  WP_004247847.1, WP_020946095.1 | Phage antitermination proteins |
| WP_020945706.1 | Peripheral inner membrane phage-shock protein |
| WP_020945708.1 | Phage shock protein B |
| WP_004243102.1 | Phage shock protein PspA |
| WP_004248148.1 | Phage shock protein operon transcriptional activator |
| WP_012367604.1 | Exodeoxyribonuclease VIII (phage-related exonuclease) (Cas system-associated protein Cas4) |
| WP_012368747.1, WP_020946640.1 | Prophage integrases |
| WP_023843592.1, WP_020946090.1, WP_023843547.1 | Phage protein (endopeptidase/lysis protein) |
| WP_004248367.1 | Phage holin family (Lysis protein S) |
| WP_004248370.1, WP_012367805.1 | Phage repressor protein |
| WP_004245719.1 | Membrane protein FxsA (suppressor of F exclusion of phage T7) |
| WP_004246253.1 | Phage-related DNA-binding protein |
| WP_004247455.1 | Phage recombination protein |
| WP_012367808.1 | Phage DNA adenine-methylase |
| WP_020945122.1, WP_020945123.1  WP_020945198.1, WP_020945199.1  WP_020945207.1, WP_020945231.1  WP_020945232.1, WP_020945527.1  WP_004249129.1, WP_020946378.1  WP_012367730.1, WP_012368790.1  WP_012368720.1, WP_012368599.1  WP_012368857.1, WP_012367824.1  WP_012368602.1, WP_012368697.1  WP_012367824.1 | Transposases |
| WP_012367825.1, WP_012368611.1  WP_012368789.1, WP_012368598.1  WP_012368698.1, | DNA cytosine methyltransferase (Transposon related) |
| WP_012367770.1, WP_004251263.1  WP_012367803.1, | Integrase |
| **Chemotaxis** | |
| **GenBank Reference sequence/**  **Accession Number** | **Corresponding Protein** |
| KXB98839.1 | Putative methyl-accepting chemotaxis protein I |
| AGS60240.1 | Chemotaxis regulator CheZ |
| AGS60241.1 | Chemotaxis response regulator (REC) |
| WP_004243552.1 | Chemotaxis response regulator protein-glutamate methylesterase (REC) |
| WP_004243555.1 | Chemotaxis protein-glutamate O-methyltransferase (CheR_N, CheR, AdoMet_MTases) |
| AGS60244.1, AGS60245.1, WP_020946214.1  WP_012368515.1  WP_020946365.1 | Methyl-accepting chemotaxis protein  (Tar_Tsr_LBD, HAMP, MCP_signal, CheW) |
| WP_004243558.1 | Chemotaxis protein CheW, CheA |
| WP_020945888.1 | Chemotaxis protein CheA  (HPT, CheY-binding, H-kinase_dim, HATPase_c, CheA_reg) |
| WP_004243565.1 | Flagellar motor protein MotB  (MotB, MotB_plug, OmpA_C-like) |
