# Supplementary material for: Genome sequencing and analysis of the first spontaneous Nanosilver resistant bacterium Proteus mirabilis strain SCDR1
Source: Antimicrob Resist Infect Control. 2017 Nov 23;6:119. doi: 10.1186/s13756-017-0277-x (PMC5701452; doi:10.1186/s13756-017-0277-x)
Supplement: Supplementary file 5 — Strict Antibiotic resistance analysis of Proteus mirabilis SCDR1. (DOCX 17 kb) [file 13756_2017_277_MOESM5_ESM.docx]

**Table S5: Strict Antibiotic resistance analysis of Proteus mirabilis SCDR1**

| **Best Hit e-value** | **Best Hit ARO** | **Best Identities** | **ARO name** | **SNP** | **Best Hit ARO category** | **AR0 category** |
| --- | --- | --- | --- | --- | --- | --- |
| 1.19E-135 | cpxR | 86 | cpxR | n/a | efflux pump conferring antibiotic resistance; aminocoumarin resistance gene; aminoglycoside resistance gene; gene modulating antibiotic efflux | aminocoumarin resistance gene; aminoglycoside resistance gene; efflux pump conferring antibiotic resistance; gene modulating antibiotic efflux |
| 5.26E-60 | Bifidobacteria intrinsic ileS conferring resistance to mupirocin | 25 | Bifidobacteria intrinsic ileS conferring resistance to mupirocin | n/a | mupirocin resistance gene | mupirocin resistance gene |
| 0 | adeG | 61 | mtrD, adeB, ceoB, mdsB, smeE, mexY, smeB, amrB, mexQ, acrB, adeG, acrF, mdtF, acrD, mexF, mexD, mexB, cmeB, adeJ | n/a | efflux pump conferring antibiotic resistance; tetracycline resistance gene; fluoroquinolone resistance gene | aminocoumarin resistance gene; aminoglycoside resistance gene; antibiotic resistance gene cluster, cassette, or operon; beta-lactam resistance gene; chloramphenicol resistance gene; efflux pump conferring antibiotic resistance; erythromycin resistance efflux pump; fluoroquinolone resistance gene; lincosamide resistance gene; macrolide resistance gene; polymyxin resistance gene; rifampin resistance gene; tetracycline resistance gene; trimethoprim resistance gene |
| 0 | mexW | 77 | mdsB, mexW, mexI | n/a | efflux pump conferring antibiotic resistance; chloramphenicol resistance gene; tetracycline resistance gene; fluoroquinolone resistance gene | beta-lactam resistance gene; chloramphenicol resistance gene; efflux pump conferring antibiotic resistance; fluoroquinolone resistance gene; tetracycline resistance gene |
| 0 | Escherichia coli mutant GlpT conferring resistance to fosfomycin | 87 | Escherichia coli mutant GlpT conferring resistance to fosfomycin | E448K | fosfomycin resistance gene; antibiotic resistant gene variant or mutant | antibiotic resistant gene variant or mutant; fosfomycin resistance gene |
| 6.84E-161 | baeS | 53 | baeS | n/a | efflux pump conferring antibiotic resistance; aminocoumarin resistance gene; aminoglycoside resistance gene; gene modulating antibiotic efflux | aminocoumarin resistance gene; aminoglycoside resistance gene; efflux pump conferring antibiotic resistance; gene modulating antibiotic efflux |
| 0 | PmrF | 72 | PmrF | n/a | polymyxin resistance gene; gene altering cell wall charge conferring antibiotic resistance | gene altering cell wall charge conferring antibiotic resistance; polymyxin resistance gene |
| 3.14E-131 | Streptomyces cinnamoneus EF-Tu mutants conferring resistance to elfamycin | 71 | Streptomyces cinnamoneus EF-Tu mutants conferring resistance to elfamycin | n/a | gene involved in self-resistance to antibiotic; antibiotic resistant gene variant or mutant; elfamycin resistance gene | antibiotic resistant gene variant or mutant; elfamycin resistance gene; gene involved in self-resistance to antibiotic |
| 1.33E-154 | CRP | 98 | CRP | n/a | efflux pump conferring antibiotic resistance; macrolide resistance gene; beta-lactam resistance gene; gene modulating antibiotic efflux; fluoroquinolone resistance gene | beta-lactam resistance gene; efflux pump conferring antibiotic resistance; fluoroquinolone resistance gene; gene modulating antibiotic efflux; macrolide resistance gene |
| 2.45E-107 | baeR | 66 | smeR, baeR | n/a | efflux pump conferring antibiotic resistance; aminocoumarin resistance gene; aminoglycoside resistance gene; gene modulating antibiotic efflux | aminocoumarin resistance gene; aminoglycoside resistance gene; beta-lactam resistance gene; efflux pump conferring antibiotic resistance; fluoroquinolone resistance gene; gene modulating antibiotic efflux |
| 0 | tetJ | 94 | tetJ | n/a | efflux pump conferring antibiotic resistance; tetracycline resistance gene | efflux pump conferring antibiotic resistance; tetracycline resistance gene |
| 1.35E-147 | cat | 91 | catI, cat | n/a | chloramphenicol resistance gene; antibiotic inactivation enzyme | antibiotic inactivation enzyme; chloramphenicol resistance gene |
| 0 | adeG | 76 | mtrD, adeB, ceoB, mdsB, smeE, mexY, smeB, amrB, mexQ, acrB, adeG, acrF, mdtF, acrD, mexF, mexD, mexB, cmeB, adeJ | n/a | efflux pump conferring antibiotic resistance; tetracycline resistance gene; fluoroquinolone resistance gene | aminocoumarin resistance gene; aminoglycoside resistance gene; antibiotic resistance gene cluster, cassette, or operon; beta-lactam resistance gene; chloramphenicol resistance gene; efflux pump conferring antibiotic resistance; erythromycin resistance efflux pump; fluoroquinolone resistance gene; lincosamide resistance gene; macrolide resistance gene; polymyxin resistance gene; rifampin resistance gene; tetracycline resistance gene; trimethoprim resistance gene |
| 3.39E-128 | leuO | 58 | leuO | n/a | sulfonamide resistance gene; gene modulating antibiotic efflux | gene modulating antibiotic efflux; sulfonamide resistance gene |
| 0 | emrB | 72 | emrY, emrB | n/a | efflux pump conferring antibiotic resistance; fluoroquinolone resistance gene | efflux pump conferring antibiotic resistance; fluoroquinolone resistance gene; tetracycline resistance gene |
| 0 | arnA | 68 | arnA | n/a | polymyxin resistance gene; gene altering cell wall charge conferring antibiotic resistance | gene altering cell wall charge conferring antibiotic resistance; polymyxin resistance gene |
| 0 | mdtH | 73 | mdtH | n/a | efflux pump conferring antibiotic resistance | efflux pump conferring antibiotic resistance |
| 0 | cpxA | 79 | cpxA | n/a | efflux pump conferring antibiotic resistance; aminocoumarin resistance gene; aminoglycoside resistance gene; gene modulating antibiotic efflux | aminocoumarin resistance gene; aminoglycoside resistance gene; efflux pump conferring antibiotic resistance; gene modulating antibiotic efflux |
| 0 | tolC | 67 | tolC | n/a | chloramphenicol resistance gene; macrolide resistance gene; fluoroquinolone resistance gene; efflux pump conferring antibiotic resistance; aminocoumarin resistance gene; tetracycline resistance gene; rifampin resistance gene; beta-lactam resistance gene | aminocoumarin resistance gene; beta-lactam resistance gene; chloramphenicol resistance gene; efflux pump conferring antibiotic resistance; fluoroquinolone resistance gene; macrolide resistance gene; rifampin resistance gene; tetracycline resistance gene |
| 8.52E-155 | mdtA | 61 | mdtA | n/a | efflux pump conferring antibiotic resistance; aminocoumarin resistance gene | aminocoumarin resistance gene; efflux pump conferring antibiotic resistance |
| 0 | mdtC | 75 | mdtC, mdtB, mexN | n/a | efflux pump conferring antibiotic resistance; aminocoumarin resistance gene | aminocoumarin resistance gene; chloramphenicol resistance gene; efflux pump conferring antibiotic resistance |
| 0 | rosB | 66 | rosB | n/a | efflux pump conferring antibiotic resistance; polymyxin resistance gene | efflux pump conferring antibiotic resistance; polymyxin resistance gene |
| 2.39E-166 | emrD | 65 | emrD | n/a | efflux pump conferring antibiotic resistance | efflux pump conferring antibiotic resistance |
| 4.97E-134 | mdtE | 51 | smeA, mexC, mdtE, adeA | n/a | efflux pump conferring antibiotic resistance; antibiotic resistance gene cluster, cassette, or operon; beta-lactam resistance gene; macrolide resistance gene; fluoroquinolone resistance gene | aminoglycoside resistance gene; antibiotic resistance gene cluster, cassette, or operon; beta-lactam resistance gene; chloramphenicol resistance gene; efflux pump conferring antibiotic resistance; fluoroquinolone resistance gene; macrolide resistance gene; tetracycline resistance gene; trimethoprim resistance gene |
| 0 | macB | 49 | macB | n/a | efflux pump conferring antibiotic resistance; macrolide resistance gene | efflux pump conferring antibiotic resistance; macrolide resistance gene |
| 2.38E-123 | mexH | 54 | mexV, mexH | n/a | efflux pump conferring antibiotic resistance; fluoroquinolone resistance gene | chloramphenicol resistance gene; efflux pump conferring antibiotic resistance; fluoroquinolone resistance gene; tetracycline resistance gene |
| 4.03E-105 | aminocoumarin resistant cysB | 47 | aminocoumarin resistant cysB | n/a | aminocoumarin resistance gene | aminocoumarin resistance gene |
| 0 | mdtC | 72 | mdtC, mdtB, mexN | n/a | efflux pump conferring antibiotic resistance; aminocoumarin resistance gene | aminocoumarin resistance gene; chloramphenicol resistance gene; efflux pump conferring antibiotic resistance |
| 1.52E-166 | PmrE | 78 | PmrE | n/a | polymyxin resistance gene; gene altering cell wall charge conferring antibiotic resistance | gene altering cell wall charge conferring antibiotic resistance; polymyxin resistance gene |
| 0 | aminocoumarin resistant alaS | 77 | aminocoumarin resistant alaS | n/a | aminocoumarin resistance gene | aminocoumarin resistance gene |
| 0 | mfd | 76 | mfd | n/a | antibiotic target protection protein; fluoroquinolone resistance gene | antibiotic target protection protein; fluoroquinolone resistance gene |
| 0 | Mycobacterium tuberculosis gyrA conferring resistance to fluoroquinolones | 43 | Mycobacterium tuberculosis gyrA conferring resistance to fluoroquinolones | S95T | antibiotic resistant gene variant or mutant; fluoroquinolone resistance gene | antibiotic resistant gene variant or mutant; fluoroquinolone resistance gene |
| 4.31E-72 | bcr-1 | 38 | bcr-1 | n/a | efflux pump conferring antibiotic resistance | efflux pump conferring antibiotic resistance |
| 0 | aminocoumarin resistant cysB | 76 | aminocoumarin resistant cysB | n/a | aminocoumarin resistance gene | aminocoumarin resistance gene |

**ARO: Antibiotic Resistance Ontology**

**Model_type used is** protein homolog model or protein variant model
