# Supplementary material for: Genome sequencing and analysis of the first spontaneous Nanosilver resistant bacterium Proteus mirabilis strain SCDR1
Source: Antimicrob Resist Infect Control. 2017 Nov 23;6:119. doi: 10.1186/s13756-017-0277-x (PMC5701452; doi:10.1186/s13756-017-0277-x)
Supplement: Supplementary file 6 — Modified loose Antibiotic resistance analysis of Proteus mirabilis SCDR1. (DOCX 65 kb) [file 13756_2017_277_MOESM6_ESM.docx]

**Table S6: Modified loose Antibiotic resistance analysis of Proteus mirabilis SCDR1**

| **Best Hit e-value** | **Best Hit ARO** | **ARO name** | **Best Hit ARO category** |
| --- | --- | --- | --- |
| 1.57E-61 | salA | srmB, FomB, otrC, novA, lmrD, mel, lsaB, vgaALC, lsaC, lsaE, salA, vgaD, vgaE, vgaB, msrC, lsaA, msrA, oleB, oleC, IMP-29, MUS-1 beta-lactamase, lmrC, MUS-2 beta-lactamase, tlrC, carA, sav1866, msrE, bcrA, macB, vgaA | efflux pump conferring antibiotic resistance; lincosamide resistance gene; streptogramin resistance gene; pleuromutilin resistance gene |
| 6.99E-33 | ramA | tetB(P), marA, vanTN, adiY, CfxA5, CfxA4, CfxA3, CfxA2, tetD, gadX, adeJ, dfrA3, CfxA, gadW, ramA, robA | chloramphenicol resistance gene; gene modulating antibiotic efflux; gene modulating permeability to antibiotic; fluoroquinolone resistance gene; efflux pump conferring antibiotic resistance; tetracycline resistance gene; rifampin resistance gene; beta-lactam resistance gene |
| 7.63E-26 | sav1866 | srmB, FosC, otrC, novA, mel, lmrD, lsaA, lsaB, vgaALC, lsaC, salA, vgaD, msrE, vgaB, msrC, lsaE, oleB, oleC, vgaA, lmrC, tlrC, msrA, sav1866, vanA, bcrA, vgaE, macB, carA | efflux pump conferring antibiotic resistance |
| 8.57E-29 | cpxA | cpxA, adeS, PmrB, arlS, baeS, vanSD, vanSG, vanSF, vanSE, evgS, vanSC, vanSB, vanSA, vanSO, vanSN, vanSM, vanSL, smeS, vanSI | efflux pump conferring antibiotic resistance; aminocoumarin resistance gene; aminoglycoside resistance gene; gene modulating antibiotic efflux |
| 5.00E-22 | rosB | tetX, rosB, MUS-1 beta-lactamase | efflux pump conferring antibiotic resistance; polymyxin resistance gene |
| 3.25E-05 | rosB | PBP1b, rosB, tetG | efflux pump conferring antibiotic resistance; polymyxin resistance gene |
| 1.71E-05 | vanG | vanC, vanE, vanG, AAC(6')-IIb, vanL, vanN | glycopeptide resistance gene; antibiotic resistance gene cluster, cassette, or operon; gene conferring antibiotic resistance via molecular bypass |
| 1.76E-21 | vgaD | Bifidobacteria intrinsic ileS conferring resistance to mupirocin, srmB, otrC, novA, mel, vanTE, APH(9)-Ib, lsaA, vgaD, vgaALC, msrE, salA, lsaB, lsaC, vgaB, lsaE, msrC, oleB, oleC, vgaA, lmrC, tlrC, msrA, sav1866, bcrA, vgaE, macB, carA, mdtF | efflux pump conferring antibiotic resistance; streptogramin resistance gene; pleuromutilin resistance gene |
| 1.33E-154 | CRP | CRP | efflux pump conferring antibiotic resistance; macrolide resistance gene; beta-lactam resistance gene; gene modulating antibiotic efflux; fluoroquinolone resistance gene |
| 9.29E-11 | evgA | evgA, OXA-361, AAC(6')-IIc, gadE, sdiA | gene modulating antibiotic efflux; macrolide resistance gene; fluoroquinolone resistance gene; efflux pump conferring antibiotic resistance; tetracycline resistance gene; beta-lactam resistance gene |
| 1.15E-12 | aadK | dfrF, aadK, mexI, ANT(6)-Ia, ANT(6)-Ib, aad(6) | antibiotic inactivation enzyme; aminoglycoside resistance gene |
| 4.02E-15 | tetH | clbB, cmlA1, tet42, tet43, tet40, tet41, norB, norA, qacB, qacA, emrD, tetC, mdtH, mdfA, blt, emeA, fexA, tetG, tet39, pmrA efflux pump, tet33, tet31, tet30, tetY, tetZ, floR, otrB, cmlA6, tetH, cmlA4, cmlA5, mdtM, tetL, mdtD, tetA, tcr3, tetB, tetE, lmrB, facT, mdtL, bmr, pp-flo, cmlA, cmlB, tetK, qepA, tcmA, bcr-1, cmrA, cmr, tetJ | efflux pump conferring antibiotic resistance; tetracycline resistance gene |
| 1.36E-31 | macB | srmB, otrC, novA, mel, lsaA, lsaB, vgaALC, msrE, salA, vgaD, lsaC, vgaB, lsaE, msrC, oleB, oleC, lmrD, lmrC, vgaA, tlrC, sav1866, bcrA, vgaE, macB, carA | efflux pump conferring antibiotic resistance; macrolide resistance gene |
| 4.03E-29 | Mycobacterium tuberculosis ndh mutant conferring resistance to isoniazid | SHV-74, SHV-77, SHV-76, SHV-70, SHV-79, norA, SHV-144, SHV-142, SHV-40, OKP-B-18, OKP-B-17, OKP-B-13, OKP-B-11, OKP-B-10, SHV-173, SHV-172, SHV-178, OKP-A-12, OKP-A-13, OKP-A-10, OKP-A-11, OKP-A-16, OKP-A-14, OKP-B-20, SHV-56, SHV-53, SHV-52, SHV-160, SHV-2A, SHV-25, SHV-123, SHV-119, SHV-110, SHV-182, SHV-183, SHV-31, SHV-35, SHV-37, SHV-36, SHV-108, SHV-109, SHV-107, Mycobacterium tuberculosis ndh mutant conferring resistance to isoniazid, SHV-135, SHV-134, SHV-94, SHV-121, OXA-61, OKP-A-1, OKP-A-2, OKP-A-3, OKP-A-4, OKP-A-5, OKP-A-6, OKP-A-7, OKP-A-8, OKP-A-9, SHV-128, SHV-129, SHV-89, SHV-85, SHV-86, SHV-80, SHV-81, SHV-82, OKP-B-9, OKP-B-8, OKP-B-3, OKP-B-2, OKP-B-7, OKP-B-6, OKP-B-5, OKP-B-4, SHV-66, SHV-67, SHV-64, SHV-62, SHV-60, SHV-61, SHV-69, SHV-157, SHV-155, SHV-93, SHV-92, SHV-96, SHV-159, SHV-158, SHV-13, SHV-12, SHV-11, SHV-15 | antibiotic resistant gene variant or mutant; isoniazid resistance gene |
| 1.21E-30 | macB | srmB, otrC, novA, mel, lsaA, lsaB, vgaALC, lsaC, salA, vgaD, msrE, vgaB, lsaE, msrC, tetJ, oleB, oleC, lmrD, lmrC, vgaA, tlrC, msrA, sav1866, bcrA, vgaE, macB, carA | efflux pump conferring antibiotic resistance; macrolide resistance gene |
| 7.10E-106 | Mycobacterium tuberculosis murA | Chlamydia trachomatis murA, Mycobacterium tuberculosis murA, tetZ, cmlB1 | fosfomycin resistance gene |
| 3.36E-38 | tet34 | OXA-347, tet34, vanHF | gene conferring antibiotic resistance via molecular bypass |
| 3.49E-07 | PmrF | PmrF, CAU-1 beta-lactamase | polymyxin resistance gene; gene altering cell wall charge conferring antibiotic resistance |
| 1.34E-50 | robA | marA, ErmF, tetD, gadX, gadW, ramA, robA | chloramphenicol resistance gene; gene modulating antibiotic efflux; fluoroquinolone resistance gene; efflux pump conferring antibiotic resistance; tetracycline resistance gene; rifampin resistance gene; beta-lactam resistance gene |
| 3.58E-18 | macB | srmB, otrC, novA, mel, otrA, lsaA, tet36, vgaD, vgaALC, lsaC, AAC(6')-31, salA, lsaB, msrE, vgaB, lsaE, msrC, oleB, oleC, lmrD, lmrC, vgaA, tlrC, msrA, OXY-3-1, sav1866, bcrA, vgaE, macB, carA | efflux pump conferring antibiotic resistance; macrolide resistance gene |
| 5.91E-08 | ramA | marA, AAC(3)-VIa, adiY, lsaC, tetD, gadX, mexF, gadW, ramA, robA | chloramphenicol resistance gene; gene modulating antibiotic efflux; gene modulating permeability to antibiotic; fluoroquinolone resistance gene; efflux pump conferring antibiotic resistance; tetracycline resistance gene; rifampin resistance gene; beta-lactam resistance gene |
| 2.12E-05 | lmrC | OpmH, sav1866, lmrC, lsaC, lsaE, novA, lmrD, cmeC | efflux pump conferring antibiotic resistance; lincosamide resistance gene |
| 2.36E-18 | macB | srmB, otrC, novA, mel, lsaA, lsaB, vgaALC, lsaC, salA, vgaD, msrE, vgaB, lsaE, msrC, oleB, oleC, lmrD, lmrC, vgaA, tlrC, msrA, tmrB, sav1866, bcrA, vgaE, macB, carA | efflux pump conferring antibiotic resistance; macrolide resistance gene |
| 3.79E-10 | carA | srmB, FomB, otrC, novA, mel, lsaA, vanRI, vgaD, vgaALC, lsaC, salA, lsaB, msrE, vgaB, msrC, vgaA, mdtC, oleB, oleC, lmrD, lmrC, tlrC, msrA, sav1866, bcrA, vgaE, macB, carA | efflux pump conferring antibiotic resistance; macrolide resistance gene |
| 1.93E-24 | baeS | evgS, msrE, adeS, cphA5, PmrB, arlS, baeS, smeS, vanSG, vanSF, vanSE, vanSD, vanSC, vanSB, vanSA, vanSO, vanSN, vanSM, vanSL, cpxA, vanSI | efflux pump conferring antibiotic resistance; aminocoumarin resistance gene; aminoglycoside resistance gene; gene modulating antibiotic efflux |
| 1.10E-17 | tet34 | fyuA, AAC(3)-VIIIa, tetB(P), tet34 | gene conferring antibiotic resistance via molecular bypass |
| 5.81E-07 | adeN | nalD, lsaA, rmtF, tet41, mexL, adeB, mtrR, adeN | chloramphenicol resistance gene; gene modulating antibiotic efflux; lincosamide resistance gene; macrolide resistance gene; fluoroquinolone resistance gene; efflux pump conferring antibiotic resistance; aminocoumarin resistance gene; tetracycline resistance gene; rifampin resistance gene; beta-lactam resistance gene; trimethoprim resistance gene |
| 3.01E-16 | qacH | ykkD, ykkC, oprM, amrB, qacH, TriC, emrE, abeS | efflux pump conferring antibiotic resistance; fluoroquinolone resistance gene |
| 0 | tetJ | tetJ | efflux pump conferring antibiotic resistance; tetracycline resistance gene |
| 1.70E-28 | bcrA | srmB, otrC, novA, mel, lsaA, vgaD, vgaALC, msrE, salA, lsaB, lsaC, vgaB, lsaE, msrC, oleB, oleC, lmrD, lmrC, vgaA, tlrC, msrA, sav1866, bcrA, vgaE, macB, carA | efflux pump conferring antibiotic resistance; peptide antibiotic resistance gene |
| 1.50E-08 | PEDO-2 | LRA-12, LRA-17, LRA-19, OXA-232, IMP-30, IMP-32, IMP-34, IMP-38, IMP-25, IMP-24, VIM-29, VIM-28, VIM-25, VIM-24, VIM-27, VIM-26, VIM-20, VIM-23, IMP-21, THIN-B beta-lactamase, IMP-29, IMP-28, IMP-27, IMP-26, APH(2'')-Ie, CPS-1, IMP-22, SMB-1 beta-lactamase, IMP-20, VIM-18, VIM-19, VIM-10, VIM-11, VIM-12, VIM-13, VIM-14, VIM-15, VIM-16, VIM-17, DIM-1, OXA-181, IMP-51, BJP-1, SIM-1 beta-lactamase, OXA-54, AIM-1, IMP-48, IMP-41, IMP-40, IMP-43, IMP-42, IMP-44, IMP-47, LRA-9, LRA-8, LRA-3, LRA-2, SFB-1, FEZ-1 beta-lactamase, CAU-1 beta-lactamase, GOB-1 beta-lactamase, SPG-1, VIM-8, VIM-9, VIM-6, VIM-7, VIM-4, VIM-5, VIM-2, VIM-3, VIM-1, ESP-1, IMP-2, KHM-1 beta-lactamase, VIM-39, BcII, GIM-2, GIM-1, IMP-8, IMP-14, IMP-15, IMP-12, IMP-10, IMP-11, VIM-43, VIM-42, IMP-19, Rm3 beta-lactamase, MSI-1, APH(2'')-IVa, L1 beta-lactamase, PEDO-2, PEDO-1, IMP-4, IMP-5, IMP-6, IMP-7, IMP-1, VIM-38, IMP-3, VIM-36, VIM-37, VIM-34, VIM-35, VIM-32, VIM-33, VIM-30, VIM-31, vanHA | antibiotic inactivation enzyme; beta-lactam resistance gene |
| 6.38E-44 | Enterococcus faecium cls conferring resistance to daptomycin | Enterococcus faecium cls conferring resistance to daptomycin, FomA | antibiotic resistant gene variant or mutant; lipopeptide antibiotic resistance gene |
| 7.37E-11 | aminocoumarin resistant cysB | leuO, CARB-7, NmcR, APH(3')-VIIa, aminocoumarin resistant cysB | aminocoumarin resistance gene |
| 3.13E-26 | bcrA | srmB, otrC, novA, mel, salA, lsaB, vgaALC, msrE, lsaA, vgaD, lsaC, vgaB, lsaE, msrC, oleB, oleC, IND-10, lmrD, lmrC, vgaA, IND-1, tlrC, msrA, mecR1, IND-5, IND-7, sav1866, IND-6, IND-9, IND-8, bcrA, vgaE, macB, carA | efflux pump conferring antibiotic resistance; peptide antibiotic resistance gene |
| 3.87E-23 | lmrD | srmB, otrC, novA, BEL-3, BEL-2, BEL-1, mel, lsaA, lsaB, vgaALC, lsaC, salA, vgaD, msrE, vgaB, msrC, lsaE, oleB, oleC, lmrD, lmrC, vgaA, tlrC, msrA, sav1866, bcrA, vgaE, macB, carA | efflux pump conferring antibiotic resistance; lincosamide resistance gene |
| 4.77E-05 | leuO | leuO, mexT | sulfonamide resistance gene; gene modulating antibiotic efflux |
| 9.75E-07 | evgA | evgA, sav1866, adeR, mecB, vanRE, sdiA | gene modulating antibiotic efflux; macrolide resistance gene; fluoroquinolone resistance gene; efflux pump conferring antibiotic resistance; tetracycline resistance gene; beta-lactam resistance gene |
| 6.26E-06 | FosB | FosA2, FosK, FosA, FosB, FosX, FosA3, bleomycin resistance protein (BRP), mdtG, FosA4, FosA5, pmrA efflux pump, FosC2, FosB3, vanRN | antibiotic inactivation enzyme; fosfomycin resistance gene |
| 3.52E-06 | fyuA | Bifidobacteria intrinsic ileS conferring resistance to mupirocin, fyuA, Clostridium perfringens mprF | tetracycline resistance gene; aminoglycoside resistance gene |
| 0 | aminocoumarin resistant alaS | aminocoumarin resistant alaS | aminocoumarin resistance gene |
| 4.85E-09 | leuO | leuO, Mycobacterium tuberculosis murA, NmcR | sulfonamide resistance gene; gene modulating antibiotic efflux |
| 3.76063 | mphC | mphC | macrolide resistance gene; antibiotic inactivation enzyme |
| 5.12E-13 | PmrE | tetX, SFB-1, OXA-215, PmrE, OXA-214 | polymyxin resistance gene; gene altering cell wall charge conferring antibiotic resistance |
| 2.24E-08 | mexT | leuO, vgaALC, mexT, vgaA | efflux pump conferring antibiotic resistance; chloramphenicol resistance gene; trimethoprim resistance gene; gene modulating antibiotic efflux; fluoroquinolone resistance gene |
| 2.34E-22 | oleC | srmB, otrC, novA, NDM-9, NDM-8, NDM-5, NDM-4, NDM-7, NDM-6, NDM-1, NDM-2, lmrD, bacA, mel, vgaD, NDM-12, vgaALC, msrE, salA, lsaB, lsaC, vgaB, msrC, lsaA, lsaE, oleB, oleC, msrA, lmrC, vgaA, tlrC, sav1866, NDM-10, bcrA, vgaE, macB, mexJ, carA | efflux pump conferring antibiotic resistance |
| 2.40648 | oprM | FomB, mphG, IND-5, oprM | erythromycin resistance efflux pump; chloramphenicol resistance gene; trimethoprim resistance gene; macrolide resistance gene; aminoglycoside resistance gene; fluoroquinolone resistance gene; efflux pump conferring antibiotic resistance; aminocoumarin resistance gene; tetracycline resistance gene; polymyxin resistance gene; beta-lactam resistance gene |
| 2.20E-12 | Bifidobacteria intrinsic ileS conferring resistance to mupirocin | Bifidobacteria intrinsic ileS conferring resistance to mupirocin, FOX-10, FOX-5, FOX-7, FOX-1, macA, FOX-2 | mupirocin resistance gene |
| 1.98E-59 | salA | srmB, otrC, novA, mel, lmrD, lsaA, mecB, lsaB, vgaALC, lsaC, salA, vgaD, msrE, vgaB, msrC, lsaE, oleB, oleC, vgaA, lmrC, tlrC, msrA, sav1866, bcrA, vgaE, macB, carA | efflux pump conferring antibiotic resistance; lincosamide resistance gene; streptogramin resistance gene; pleuromutilin resistance gene |
| 3.55E-12 | mfd | mfd, mecB, sul1, amrB, vanRD, vanRG, mexJ, mexD | antibiotic target protection protein; fluoroquinolone resistance gene |
| 3.65E-13 | oleC | srmB, otrC, novA, mel, vgaALC, vgaE, salA, vgaD, msrE, vgaB, msrC, vgaA, oleB, oleC, lmrD, lmrC, tlrC, sav1866, Erm(36), bcrA, macB, carA, OXA-184 | efflux pump conferring antibiotic resistance |
| 2.77E-13 | robA | marA, adiY, tetD, gadX, gadW, ramA, robA | chloramphenicol resistance gene; gene modulating antibiotic efflux; fluoroquinolone resistance gene; efflux pump conferring antibiotic resistance; tetracycline resistance gene; rifampin resistance gene; beta-lactam resistance gene |
| 6.37E-05 | mtrR | acrS, mexZ, nalD, sul3, APH(9)-Ia, mexL, mtrR, adeN, vanHM | efflux pump conferring antibiotic resistance; gene modulating antibiotic efflux |
| 5.22E-21 | dfrE | FomA, VCC-1, dfrE, oprN | antibiotic target replacement protein; trimethoprim resistance gene |
| 3.54E-20 | Erm(34) | Erm(39), Erm(35), Erm(42), IMP-31, IMP-35, Erm(37), Erm(31), ErmU, Erm(33), ErmH, Erm(34), ErmN, ErmO, Erm(41), ErmB, ErmC, ErmA, ErmF, ErmG, ErmD, Erm(43), ErmX, ErmY, ErmE, ErmR, ErmS, ErmQ, ErmV, ErmW, ErmT, Erm(38), Erm(36), Erm(30) | antibiotic target modifying enzyme; lincosamide resistance gene; macrolide resistance gene; streptogramin resistance gene |
| 9.66E-05 | NmcR | leuO, mexT, NmcR, vanSB, LRA-18, cmrA | antibiotic inactivation enzyme; beta-lactam resistance gene; gene modulating beta-lactam resistance |
| 1.01E-08 | adeF | mexH, mdsA, emrK, emrA, adeI, mexX, smeD, smeA, mdtN, cmeA, mtrC, TriA, tetT, TriB, mdtA, mexM, mexJ, adeA, mdtE, mexE, mexC, mexA, ceoA, adeF | efflux pump conferring antibiotic resistance; tetracycline resistance gene; fluoroquinolone resistance gene |
| 2.36E-23 | bcrA | srmB, otrC, vgaALC, novA, CMY-8, CMY-11, CMY-10, TEM-150, msrC, CMY-19, mel, vgaD, CMY-1, msrE, salA, CMY-9, lsaC, vgaB, lsaE, lsaA, vgaA, MOX-2, MOX-3, MOX-1, oleB, oleC, MOX-4, lmrD, lmrC, tlrC, msrA, sav1866, lsaB, bcrA, vgaE, macB, carA | efflux pump conferring antibiotic resistance; peptide antibiotic resistance gene |
| 0 | mdtC | mdtC, mdtB, mexN | efflux pump conferring antibiotic resistance; aminocoumarin resistance gene |
| 3.50E-07 | mgrA | vanSF, vanSM, mgrA, mepR, aadA5 | efflux pump conferring antibiotic resistance; tetracycline resistance gene; gene modulating antibiotic efflux; fluoroquinolone resistance gene |
| 0 | arnA | arnA | polymyxin resistance gene; gene altering cell wall charge conferring antibiotic resistance |
| 1.04E-82 | sav1866 | srmB, mfd, otrC, novA, acrD, mel, salA, Erm(31), vgaD, vgaALC, vgaE, oprM, lsaA, lsaB, lsaC, vgaB, lsaE, TriC, msrC, oleB, PBP1b, adeB, lmrD, lmrC, vgaA, ErmU, tlrC, sav1866, bcrA, macB, carA, oleC | efflux pump conferring antibiotic resistance |
| 1.19E-135 | cpxR | cpxR | efflux pump conferring antibiotic resistance; aminocoumarin resistance gene; aminoglycoside resistance gene; gene modulating antibiotic efflux |
| 1.67E-35 | evgS | adeS, cpxA, arlS, baeS, Erm(43), vanSD, vanSG, vanSF, vanSE, evgS, vanSC, vanSB, vanSA, PmrB, vanSO, vanSN, vanSM, vanSL, smeS, vanSI | gene modulating antibiotic efflux; macrolide resistance gene; fluoroquinolone resistance gene; efflux pump conferring antibiotic resistance; tetracycline resistance gene; beta-lactam resistance gene |
| 1.49E-126 | mdtG | emrY, mefB, tet42, rosA, tet41, BEL-3, cmlB1, qacB, emrB, emrD, qacA, norA, mdtH, tet43, mdfA, blt, emeA, Bacillus subtilis mprF, fexA, qepA, tetG, tet39, pmrA efflux pump, tet33, tet31, cmrA, tetY, tetZ, floR, otrB, cmlA6, tetH, tetK, tetJ, mdtG, tetL, cmlA1, tetA, tcr3, tetB, tetE, lmrB, facT, bmr, cmlA, cmlB, cmlA4, cmlA5, tcmA, bcr-1, tet30 | efflux pump conferring antibiotic resistance |
| 4.28E-08 | PmrF | PmrF | polymyxin resistance gene; gene altering cell wall charge conferring antibiotic resistance |
| 7.24E-07 | fexA | mefE, mfd, mefA, mefC, emeA, cmlA1, tet42, rosA, tet41, norB, cmlB1, qacB, qacA, emrD, tetC, mdfA, blt, PBP1a, fexA, qepA, mdtM, tet39, pmrA efflux pump, tet33, tet31, cmrA, tetY, tetZ, floR, tet43, cmlA6, tetH, cmlA4, tetJ, tetL, mdtD, tetA, tcr3, tetB, facT, tetK, bmr, pp-flo, cmlA, cmlB, norA, cmlA5, tcmA, cmx, bcr-1, tet30, cmr | efflux pump conferring antibiotic resistance; phenicol resistance gene; chloramphenicol resistance gene |
| 1.39E-25 | tetB(P) | mexT, tetB(P), AAC(6')-Iae, tetQ, tetS, tet44, tetT, otrA, Streptomyces cinnamoneus EF-Tu mutants conferring resistance to elfamycin, tetM, tetW, tetO, tet36, tet32 | antibiotic target protection protein; tetracycline resistance gene |
| 0 | Mycobacterium tuberculosis gyrA conferring resistance to fluoroquinolones | Mycobacterium tuberculosis gyrA conferring resistance to fluoroquinolones | antibiotic resistant gene variant or mutant; fluoroquinolone resistance gene |
| 6.83E-38 | RlmA(II) | tlrB conferring tylosin resistance, RlmA(II), cat-TC, ErmC, ErmA, Erm(35), Erm(43), cat, ErmY, chrB, Erm(30), Erm(33), ErmF, myrA, ErmT, Erm(38) | antibiotic target modifying enzyme; gene involved in self resistance to antibiotic; macrolide resistance gene |
| 8.96E-25 | aminocoumarin resistant cysB | mdsC, IMP-30, IMP-31, IMP-14, IMP-33, IMP-34, IMP-35, IMP-10, IMP-19, IMP-24, aminocoumarin resistant cysB, tet33, mtrE, leuO, mexT, IMP-48, KHM-1 beta-lactamase, IMP-27, IMP-40, IMP-25, IMP-42, IMP-47, IMP-20, IMP-16, IMP-6, IMP-1, IMP-2, IMP-3, IMP-8, IMP-32, NmcR | aminocoumarin resistance gene |
| 2.48E-09 | macA | ceoA, mtrC, mexX, mexV, adeF, mexP, mdtA, mexM, macA, emrA, mexE, cmeA | efflux pump conferring antibiotic resistance; macrolide resistance gene |
| 7.05E-10 | AAC(2')-Ia | AAC(2')-Ia, AAC(2')-Ic, AAC(2')-Ib, AAC(6')-Ia, AAC(6')-Iaj, AAC(6')-Ih, AAC(6')-Ik, AAC(6')-Ij, AAC(6')-Iu, AAC(6')-It, AAC(6')-Iv, AAC(6')-Ir, AAC(3)-Id, AAC(6')-Ix, sat-1 | antibiotic inactivation enzyme; aminoglycoside resistance gene |
| 1.40E-15 | bcrA | srmB, otrC, novA, mel, lmrD, lsaA, vgaD, vgaALC, msrE, mexV, salA, lsaB, lsaC, vgaB, msrC, lsaE, oleB, oleC, vgaA, lmrC, tlrC, msrA, sav1866, bcrA, vgaE, macB, carA | efflux pump conferring antibiotic resistance; peptide antibiotic resistance gene |
| 6.16E-05 | AAC(6')-Ia | AAC(2')-Ia, AAC(2')-Ic, AAC(2')-Ib, AAC(6')-I30, sat-1, AAC(6')-Iaa, bacA, AAC(6')-Iaj, AAC(6')-33, opmD, ErmD, AAC(6')-Ig, AAC(6')-If, AAC(6')-Ia, AAC(6')-Iaf, AAC(6')-Ih, AAC(6')-Ik, AAC(6')-Ij, AAC(6')-Iu, AAC(6')-It, AAC(6')-Iw, AAC(6')-Iv, AAC(6')-Ip, AAC(6')-Is, AAC(6')-Ir, AAC(6')-Iy, AAC(6')-Ix | antibiotic inactivation enzyme; aminoglycoside resistance gene |
| 2.25E-08 | tet35 | vanTrL, tet35 | efflux pump conferring antibiotic resistance; tetracycline resistance gene |
| 1.07E-42 | macB | srmB, otrC, novA, mel, lsaA, CARB-10, lsaB, CARB-16, vgaALC, lsaC, salA, vgaD, msrE, vgaB, lsaE, msrC, oleB, CARB-8, CARB-5, lmrD, lmrC, vgaA, tlrC, msrA, sav1866, CARB-14, bcrA, vgaE, macB, cmx, carA, oleC | efflux pump conferring antibiotic resistance; macrolide resistance gene |
| 9.65E-05 | catB8 | catB7, catB6, vatH, catB3, catB2, vatD, vatA, catB9, cat, vatC, vatB, arnA, vatF, vatE, vanRG, catB8, catB10 | chloramphenicol resistance gene; antibiotic inactivation enzyme |
| 0 | cpxA | cpxA | efflux pump conferring antibiotic resistance; aminocoumarin resistance gene; aminoglycoside resistance gene; gene modulating antibiotic efflux |
| 3.11E-60 | mdtN | mdsA, emrK, emrA, QnrB38, QnrB32, QnrB33, QnrB30, QnrB31, QnrB36, QnrB37, QnrB34, QnrB35, mexP, mexM, mexJ, mexH, mexE, mexC, QnrB29, QnrB28, QnrB21, QnrB20, QnrB23, QnrB22, QnrB25, QnrB24, QnrB27, QnrB26, QnrB14, QnrB15, QnrB16, QnrB17, QnrB10, QnrB11, QnrB12, QnrB13, QnrB18, QnrB19, adeF, adeA, lmrD, adeI, acrA, smeA, QnrB74, QnrB72, QnrB73, QnrB70, QnrB71, QnrD1, QnrD2, cmeA, mtrC, mdtA, mdtE, mdtN, QnrB65, QnrB64, QnrB67, QnrB66, QnrB61, QnrB60, QnrB62, QnrB69, QnrB68, QnrB50, QnrB54, QnrB55, QnrB56, QnrB57, QnrB58, QnrB59, QnrB2, QnrB3, QnrB1, QnrB6, QnrB7, QnrB4, QnrB5, QnrB8, QnrB9, TriA, TriB, ceoA, QnrB49, QnrB48, QnrB47, QnrB46, QnrB45, QnrB44, QnrB43, QnrB42, QnrB41, QnrB40, macA | efflux pump conferring antibiotic resistance |
| 6.80E-47 | sul3 | sul1, sul2, sul3 | antibiotic target replacement protein; sulfonamide resistance gene |
| 1.18E-08 | CRP | CRP, pp-flo, mexY, ErmA, amrB, Erm(33) | efflux pump conferring antibiotic resistance; macrolide resistance gene; beta-lactam resistance gene; gene modulating antibiotic efflux; fluoroquinolone resistance gene |
| 5.46E-14 | tsnr | oprM, tsnr | antibiotic target modifying enzyme; peptide antibiotic resistance gene |
| 1.21E-06 | mexS | tetA, tetB, tet33, mexS, mecR1 | efflux pump conferring antibiotic resistance; chloramphenicol resistance gene; trimethoprim resistance gene; gene modulating antibiotic efflux; fluoroquinolone resistance gene |
| 8.57E-05 | iri | TEM-217, TEM-215, TEM-59, TEM-70, TEM-71, TEM-219, TEM-55, TEM-75, QnrB34, TEM-209, TEM-148, TEM-84, TEM-95, TEM-118, TEM-157, TEM-178, TEM-112, TEM-214, TEM-116, TEM-171, iri, vatD, TEM-86, tetX, TEM-48, TEM-206, FosA, TEM-188, ANT(4')-IIb, TEM-42, mdtC, TEM-127, TEM-108, gadX, TEM-120, TEM-122, TEM-128, TEM-164, TEM-163, TEM-162, TEM-117, tmrB, TEM-141, TEM-1, TEM-7, TEM-19 | rifampin resistance gene; antibiotic inactivation enzyme |
| 1.15E-06 | fyuA | rmtH, IMP-37, IMP-33, IMP-11, IMP-10, fyuA, IMP-41, IMP-40, IMP-42, IMP-44, IMP-21 | tetracycline resistance gene; aminoglycoside resistance gene |
| 0 | mexW | mdsB, mexW, mexI | efflux pump conferring antibiotic resistance; chloramphenicol resistance gene; tetracycline resistance gene; fluoroquinolone resistance gene |
| 0 | PmrF | PmrF | polymyxin resistance gene; gene altering cell wall charge conferring antibiotic resistance |
| 8.56E-23 | golS | golS, AAC(6')-Iq | efflux pump conferring antibiotic resistance; chloramphenicol resistance gene; beta-lactam resistance gene; gene modulating antibiotic efflux |
| 1.52E-166 | PmrE | PmrE | polymyxin resistance gene; gene altering cell wall charge conferring antibiotic resistance |
| 8.25E-07 | adeR | AAC(2')-Ia, evgA, mtrA, adeR, mexY, vanRM, arlR, amrB, Clostridium perfringens mprF, vanRB, evgS, vanRF, cpxR, kdpE, smeR, vanRO | efflux pump conferring antibiotic resistance; tetracycline resistance gene; gene modulating antibiotic efflux |
| 9.52E-24 | qacH | ykkD, abeS, ykkC, mel, Listeria monocytogenes mprF, qacH, tcr3, emrE | efflux pump conferring antibiotic resistance; fluoroquinolone resistance gene |
| 2.60E-09 | sat-3 | sat-1, AAC(6')-Ig, AAC(1), AAC(6')-Iad, AAC(6')-Ic, ceoB, AAC(6')-Iak, AAC(6')-Ih, AAC(6')-Ik, sat-3, tcr3, AAC(6')-It, dfrA8, AAC(6')-Iv, AAC(6')-Is, tolC, sat-4, AAC(3)-Ia, AAC(3)-Ic, AAC(6')-Iz | streptothricin resistance gene; antibiotic inactivation enzyme |
| 9.61E-05 | mgrA | emrR, mgrA | efflux pump conferring antibiotic resistance; tetracycline resistance gene; gene modulating antibiotic efflux; fluoroquinolone resistance gene |
| 5.91E-14 | novA | srmB, otrC, novA, NDM-9, NDM-8, NDM-4, NDM-7, NDM-6, NDM-1, NDM-3, NDM-2, lmrD, mel, lsaB, NDM-13, NDM-12, vgaALC, msrE, salA, vgaD, lsaC, vgaB, msrC, lsaA, lsaE, oleB, oleC, vgaA, lmrC, tlrC, msrA, sav1866, FosB, bcrA, vgaE, macB, carA | efflux pump conferring antibiotic resistance; aminocoumarin resistance gene |
| 6.15E-05 | fyuA | fyuA, PBP1a | tetracycline resistance gene; aminoglycoside resistance gene |
| 8.52E-155 | mdtA | mdtA | efflux pump conferring antibiotic resistance; aminocoumarin resistance gene |
| 3.11E-15 | mexT | leuO, mexT, NmcR, tet44, adeK | efflux pump conferring antibiotic resistance; chloramphenicol resistance gene; trimethoprim resistance gene; gene modulating antibiotic efflux; fluoroquinolone resistance gene |
| 6.23E-05 | acrS | acrS, mexL, nalD, nfxB, mtrR | chloramphenicol resistance gene; gene modulating antibiotic efflux; fluoroquinolone resistance gene; efflux pump conferring antibiotic resistance; tetracycline resistance gene; rifampin resistance gene; beta-lactam resistance gene |
| 6.32E-40 | macA | APH(3'')-Ib, tetO, mexH, mdsA, acrA, acrE, emrA, mtrC, smeD, smeA, mdtN, cmeA, mexX, mexY, mexV, TriA, amrA, mexP, TriB, mdtA, mexM, mexJ, adeA, mdtE, mexE, mexC, mexA, ceoA, adeF, macA | efflux pump conferring antibiotic resistance; macrolide resistance gene |
| 3.22E-14 | tlrC | OXA-47, otrC, srmB, novA, OXA-224, mel, msrC, OXA-320, OXA-33, OXA-31, lsaA, vgaD, vgaALC, lsaC, salA, lsaB, msrE, vgaB, lsaE, vgaA, OXA-1, oleB, oleC, lmrD, lmrC, tlrC, msrA, sav1866, Streptomyces rishiriensis parY mutant conferring resistance to aminocoumarin, bcrA, vgaE, macB, OXA-4, carA | efflux pump conferring antibiotic resistance; macrolide resistance gene |
| 4.97E-134 | mdtE | smeA, mexC, mdtE, adeA | efflux pump conferring antibiotic resistance; antibiotic resistance gene cluster, cassette, or operon; beta-lactam resistance gene; macrolide resistance gene; fluoroquinolone resistance gene |
| 2.49E-22 | evgA | evgA, baeR, QnrVC6, QnrVC1, evgS, QnrS5, vanRA, vanRE, vanRF, vanRI, vanRM, QnrS1, QnrS3, QnrS2, mtrA, QnrS4, QnrS7, QnrS6, QnrS9, QnrS8, arlR, cpxR, mdtG, kdpE, sdiA, smeR | gene modulating antibiotic efflux; macrolide resistance gene; fluoroquinolone resistance gene; efflux pump conferring antibiotic resistance; tetracycline resistance gene; beta-lactam resistance gene |
| 4.71E-35 | kdpE | evgA, baeR, evgS, NDM-7, tet32, vanRA, vanRB, vanRC, vanRD, vanRE, vanRF, vanRG, vanRI, vanRL, vanRM, vanRN, vanRO, mtrA, adeR, arlR, cpxR, tetO, kdpE, cmeC, smeR | aminoglycoside resistance gene |
| 1.48E-06 | vanSE | mfd, sav1866, adeS, PmrB, baeS, vanSC, OXA-61, vanSE, evgS, cpxA, vanSB, vanSA, vanSN, vanSL, smeS | glycopeptide resistance gene; antibiotic resistance gene cluster, cassette, or operon; gene conferring antibiotic resistance via molecular bypass |
| 2.00E-10 | SRT-1 | LRA-13, ACT-15, LRA-18, CMY-29, CMY-99, CMY-98, CMY-93, CMY-90, CMY-95, CMY-94, CMY-13, CMY-12, CMY-11, CMY-10, CMY-17, CMY-16, CMY-15, CMY-14, DHA-3, DHA-2, DHA-1, CMY-18, DHA-7, DHA-6, DHA-5, FOX-5, FOX-4, FOX-7, CMY-76, FOX-1, FOX-3, FOX-2, FOX-9, FOX-8, ACT-6, ACT-7, ACT-4, ACT-5, ACT-2, ACT-3, ACT-1, OCH-6, ACT-9, CMY-31, MIR-8, MIR-9, CMY-30, MIR-2, MIR-3, MIR-1, MIR-6, MIR-4, MIR-5, CMY-66, CMY-67, CMY-64, CMY-65, CMY-62, CMY-63, CMY-60, CMY-61, DHA-13, DHA-12, DHA-10, DHA-17, DHA-16, CMY-68, CMY-69, ACC-1, ACC-3, ACC-2, ACC-5, ACC-4, CMY-6, OCH-4, MIR-14, MIR-15, MIR-16, MIR-17, MIR-10, MIR-11, MIR-12, MIR-13, CMY-75, CMY-74, CMY-77, OCH-7, CMY-71, CMY-70, CMY-73, CMY-72, CMY-79, CMY-78, CMY-23, CMY-2, CMY-1, CMY-7, ACT-16, CMY-5, CMY-4, CMY-9, CMY-8, MOX-2, MOX-3, MOX-1, MOX-6, MOX-7, MOX-4, MOX-5, MOX-8, MOX-9, DHA-15, OCH-3, FOX-10, CMY-43, CMY-26, ACT-36, CMY-41, CMY-42, ACT-35, CMY-44, CMY-45, ACT-30, CMY-47, CMY-48, CMY-49, ADC-2, DHA-14, CMY-19, ACT-31, ACT-25, ACT-24, ACT-27, ACT-21, ACT-20, CMY-59, ACT-22, CMY-57, CMY-56, CMY-55, CMY-54, CMY-53, ACT-28, CMY-51, CMY-50, CMY-39, ACT-23, CMY-58, CMY-119, CMY-118, CMY-113, CMY-112, CMY-111, CMY-110, CMY-117, CMY-116, CMY-115, CMY-114, LAT-1, ACT-29, ACT-10, OCH-5, CMY-28, ACT-13, ACT-14, OCH-1, OCH-2, ACT-17, ACT-18, ACT-19, CMY-20, CMY-21, OCH-8, CMY-27, CMY-24, CMY-25, CepS beta-lactamase, SRT-2, SRT-1, CMY-108, DHA-18, CMY-100, CMY-101, CMY-102, CMY-103, CMY-104, CMY-105, CMY-40, ACT-37, DHA-19, ACT-12, DHA-22, CMY-38, DHA-20, DHA-21, AQU-1, ACT-32, CMY-33, CMY-32, CMY-35, CMY-34, CMY-37, CMY-46, CMY-22, CFE-1, PDC-10, CMY-84, CMY-85, CMY-86, CMY-87, CMY-80, CMY-81, CMY-82, CMY-83, PDC-5, PDC-4, PDC-7, PDC-6, PDC-1, PDC-3, PDC-2, PDC-9, PDC-8 | antibiotic inactivation enzyme; beta-lactam resistance gene |
| 7.33E-11 | macB | evgA, lmrD, macB | efflux pump conferring antibiotic resistance; macrolide resistance gene |
| 1.35E-69 | salA | srmB, otrC, novA, mel, lmrD, salA, vanXO, vgaD, vgaALC, msrE, lsaA, lsaB, lsaC, vgaB, msrC, lsaE, oleB, oleC, vgaA, lmrC, tlrC, msrA, sav1866, bcrA, vgaE, macB, carA | efflux pump conferring antibiotic resistance; lincosamide resistance gene; streptogramin resistance gene; pleuromutilin resistance gene |
| 3.39E-128 | leuO | leuO | sulfonamide resistance gene; gene modulating antibiotic efflux |
| 0 | aminocoumarin resistant cysB | aminocoumarin resistant cysB | aminocoumarin resistance gene |
| 1.87E-33 | macB | CepS beta-lactamase, srmB, otrC, novA, mel, lmrD, lsaA, lsaB, vgaALC, lsaC, salA, vgaD, msrE, vgaB, msrC, lsaE, adeG, oleB, oleC, mexF, vgaA, lmrC, tlrC, msrA, sav1866, ceoB, bcrA, vgaE, macB, carA | efflux pump conferring antibiotic resistance; macrolide resistance gene |
| 5.18E-13 | abeS | abeS, ykkD, qacH, emrE, norB | efflux pump conferring antibiotic resistance |
| 6.70E-09 | sat-4 | AAC(6')-Iaa, acrS, AAC(6')-If, AAC(6')-Ia, AAC(6')-Ic, AAC(3)-Ia, AAC(3)-Ib, AAC(6')-Iak, AAC(6')-Iaj, AAC(6')-Iw, AAC(1), sat-4, sat-1, AAC(6')-Iy, sat-3, AAC(6')-Iz | streptothricin resistance gene; antibiotic inactivation enzyme |
| 2.39E-166 | emrD | emrD | efflux pump conferring antibiotic resistance |
| 1.81E-27 | Staphylococcus aureus pgsA mutations conferring resistance to daptomycin | TEM-92, TEM-93, TEM-90, TEM-91, TEM-96, TEM-94, TEM-95, TEM-178, TEM-176, TEM-177, CfxA5, CfxA4, CfxA3, CfxA2, TEM-171, TEM-67, TEM-63, TEM-60, TEM-68, TEM-183, TEM-182, TEM-187, TEM-186, TEM-185, TEM-184, TEM-189, TEM-188, TEM-89, TEM-88, mexI, TEM-81, TEM-80, TEM-83, TEM-82, TEM-85, TEM-84, TEM-87, TEM-86, TEM-8, TEM-109, TEM-108, TEM-1, TEM-2, TEM-3, TEM-107, TEM-106, TEM-105, TEM-104, TEM-12, TEM-10, TEM-11, TEM-16, TEM-17, TEM-15, TEM-19, TEM-101, TEM-190, TEM-211, TEM-193, TEM-217, TEM-195, TEM-215, TEM-197, TEM-198, TEM-199, TEM-219, TEM-6, TEM-102, TEM-110, TEM-111, TEM-112, TEM-113, TEM-114, TEM-115, TEM-116, tet31, TEM-201, TEM-205, TEM-206, TEM-207, TEM-208, TEM-209, TEM-125, TEM-124, TEM-127, TEM-126, TEM-121, TEM-120, TEM-123, TEM-122, CfxA, TEM-129, TEM-128, CfxA6, TEM-34, TEM-30, TEM-33, TEM-213, SHV-186, TEM-59, TEM-57, TEM-54, TEM-55, TEM-52, TEM-53, TEM-132, TEM-133, TEM-130, TEM-131, TEM-136, TEM-137, TEM-134, TEM-135, TEM-194, TEM-138, TEM-139, TEM-216, TEM-214, Staphylococcus aureus pgsA mutations conferring resistance to daptomycin, TEM-29, TEM-28, TEM-22, TEM-21, TEM-20, TEM-26, TEM-24, TEM-49, TEM-48, TEM-45, TEM-47, TEM-40, TEM-43, TEM-42, vanXYN, TEM-147, TEM-146, TEM-145, TEM-144, TEM-143, TEM-142, TEM-141, TEM-149, TEM-148, TEM-78, TEM-79, TEM-70, TEM-71, TEM-72, TEM-73, TEM-76, TEM-77, TEM-154, TEM-155, TEM-156, TEM-157, TEM-150, TEM-151, TEM-152, TEM-153, TEM-158, TEM-159, TEM-160, TEM-163, TEM-162, TEM-164, TEM-167, TEM-166, TEM-169, TEM-168 | antibiotic resistant gene variant or mutant; lipopeptide antibiotic resistance gene |
| 2.42E-65 | carA | AAC(3)-VIIIa, otrC, srmB, novA, mel, lmrD, salA, AAC(3)-Xa, lsaB, vgaALC, lsaC, AAC(3)-VIIa, lsaA, vgaD, vgaE, vgaB, msrC, lsaE, AAC(3)-IIIb, IMP-48, oleB, oleC, adeB, vgaA, lmrC, tlrC, msrA, sav1866, Chlamydia trachomatis murA, msrE, bcrA, macB, APH(4)-Ia, carA | efflux pump conferring antibiotic resistance; macrolide resistance gene |
| 7.63E-06 | tsnr | tetY, aminocoumarin resistant alaS, tet40, tetG, tsnr | antibiotic target modifying enzyme; peptide antibiotic resistance gene |
| 5.34E-06 | acrS | acrS, nalD, mexQ, mexL, APH(2'')-IIIa, mtrR, adeN | chloramphenicol resistance gene; gene modulating antibiotic efflux; fluoroquinolone resistance gene; efflux pump conferring antibiotic resistance; tetracycline resistance gene; rifampin resistance gene; beta-lactam resistance gene |
| 1.13E-05 | macB | tet35, macB, vanHM | efflux pump conferring antibiotic resistance; macrolide resistance gene |
| 4.07E-05 | tet30 | emeA, tetK, cmx, qacB, qacA, cmrA, tet33, tet30 | efflux pump conferring antibiotic resistance; tetracycline resistance gene |
| 2.47E-06 | gadW | marA, PBP2b, adiY, gadX, gadW, tetD, robA | efflux pump conferring antibiotic resistance; macrolide resistance gene; beta-lactam resistance gene; gene modulating antibiotic efflux; fluoroquinolone resistance gene |
| 8.88E-41 | emrE | ykkD, abeS, ykkC, acrB, qacH, mexB, emrE | efflux pump conferring antibiotic resistance |
| 2.84E-23 | macB | otrC, TEM-92, TEM-93, TEM-90, TEM-91, TEM-96, TEM-94, TEM-95, msrC, TEM-177, TEM-171, TEM-63, TEM-68, TEM-182, TEM-187, TEM-186, TEM-185, TEM-184, TEM-189, TEM-188, TEM-53, TEM-89, TEM-88, TEM-81, TEM-80, TEM-83, TEM-82, TEM-85, TEM-84, TEM-87, TEM-86, TEM-8, TEM-109, TEM-108, TEM-1, TEM-101, TEM-3, TEM-107, TEM-106, TEM-105, TEM-104, TEM-12, TEM-10, TEM-11, TEM-16, TEM-17, TEM-15, TEM-19, TEM-2, TEM-190, TEM-191, TEM-211, TEM-217, TEM-216, TEM-215, TEM-197, TEM-198, TEM-199, TEM-219, TEM-6, TEM-102, TEM-7, TEM-118, TEM-110, TEM-111, TEM-112, TEM-113, TEM-114, TEM-115, TEM-116, TEM-117, mel, TEM-201, TEM-205, TEM-206, TEM-208, TEM-209, vgaD, vgaE, vgaB, vgaA, TEM-125, TEM-124, TEM-127, TEM-126, TEM-120, TEM-123, TEM-122, lmrD, TEM-129, TEM-128, TEM-34, carA, TEM-33, TEM-213, SHV-186, TEM-59, TEM-57, TEM-55, TEM-52, TEM-192, novA, TEM-132, TEM-133, TEM-130, TEM-131, TEM-136, TEM-137, TEM-134, TEM-135, TEM-138, TEM-139, TEM-195, lmrC, TEM-214, TEM-29, TEM-28, TEM-22, TEM-21, TEM-20, TEM-26, TEM-24, TEM-49, TEM-48, TEM-45, lsaA, TEM-47, msrE, TEM-40, TEM-43, msrA, oleB, oleC, tlrC, TEM-144, TEM-143, TEM-142, TEM-141, TEM-149, TEM-148, bcrA, TEM-78, srmB, TEM-70, TEM-71, TEM-72, TEM-75, TEM-76, oleR, TEM-154, TEM-155, TEM-156, TEM-157, TEM-150, TEM-151, TEM-152, TEM-153, TEM-158, TEM-159, sav1866, lsaB, vgaALC, lsaC, salA, lsaE, TEM-42, TEM-160, TEM-163, TEM-162, TEM-164, TEM-167, TEM-166, TEM-169, TEM-168, macB | efflux pump conferring antibiotic resistance; macrolide resistance gene |
| 1.79E-41 | macB | srmB, otrC, novA, mel, VIM-3, lsaA, lsaB, vgaALC, lsaC, salA, vgaD, msrE, vgaB, lsaE, msrC, oleB, oleC, lmrD, lmrC, vgaA, tlrC, msrA, sav1866, bcrA, vgaE, macB, carA, BcII | efflux pump conferring antibiotic resistance; macrolide resistance gene |
| 9.61E-28 | NmcR | cfrA, leuO, NmcR, aminocoumarin resistant cysB, mexI | antibiotic inactivation enzyme; beta-lactam resistance gene; gene modulating beta-lactam resistance |
| 2.67E-22 | salA | srmB, otrC, novA, NDM-9, NDM-6, NDM-1, NDM-3, NDM-2, msrC, lmrC, mel, vgaD, vgaALC, msrE, salA, lsaB, lsaC, vgaB, lsaE, lsaA, vgaA, oleB, oleC, APH(2'')-Ig, lmrD, kdpE, tlrC, msrA, sav1866, vanM, NDM-10, bcrA, vgaE, macB, carA, LRA-5 | efflux pump conferring antibiotic resistance; lincosamide resistance gene; streptogramin resistance gene; pleuromutilin resistance gene |
| 1.51E-08 | oleR | PDC-5, vanTG, PDC-7, PDC-6, oleR, PDC-3, PDC-2, PDC-9, PDC-8, desR, PDC-10, PDC-4, PDC-1 | gene involved in self resistance to antibiotic |
| 2.60E-14 | MCR-1 | cat-TC, MCR-1, PmrC, cat | polymyxin resistance gene; gene altering cell wall charge conferring antibiotic resistance |
| 7.90E-06 | tcr3 | mefA, tet42, tet43, tet41, acrB, norB, norA, tcr3, mdfA, blt, emeA, catB8, otrB, mdtM, tet39, pmrA efflux pump, tet33, tet31, tet30, tetY, tetZ, floR, rosA, cmlA6, tetH, cmlA4, cmlA5, mdtF, adeB, cmlA1, tetA, tetC, tetB, tetE, lmrB, facT, pp-flo, cmlA, cmlB, cmlB1, cat, tetJ, tcmA, bcr-1, tetL | efflux pump conferring antibiotic resistance; tetracycline resistance gene |
| 1.35E-147 | cat | catI, cat | chloramphenicol resistance gene; antibiotic inactivation enzyme |
| 1.28E-06 | mdtG | mefB, emeA, tet42, cmlv, bmr, norA, qepA, mdtG, tetL, blt, pmrA efflux pump, facT | efflux pump conferring antibiotic resistance |
| 3.29E-26 | Mycobacterium tuberculosis pncA mutations conferring resistance to pyrazinamide | MIR-15, MIR-16, MIR-17, MIR-10, MIR-11, MIR-12, MIR-13, Mycobacterium tuberculosis pncA mutations conferring resistance to pyrazinamide, MIR-8, MIR-9, MIR-2, MIR-3, MIR-1, MIR-6, MIR-4, MIR-5 | antibiotic resistant gene variant or mutant; pyrazinamide resistance gene |
| 0 | mdtC | mdtC, mdtB, mexN | efflux pump conferring antibiotic resistance; aminocoumarin resistance gene |
| 1.56E-21 | cmlA5 | mefA, mefB, tet42, otrB, tet41, cmlB1, emrD, tetC, mdfA, fexA, OXA-435, tetG, tet39, pmrA efflux pump, tet33, tet31, tet30, tetY, tetZ, floR, arr-8, cmlA6, tetH, cmlA4, cmlA5, mdtM, tetL, cmlA1, tetA, tcr3, tetB, tetE, lmrB, facT, mdtL, bmr, cml, pp-flo, cmlA, cmlB, Brucella suis mprF, tetK, tetJ, tcmA, cmx, arr-7, bcr-1, cmr | efflux pump conferring antibiotic resistance; chloramphenicol resistance gene |
| 0 | mfd | mfd | antibiotic target protection protein; fluoroquinolone resistance gene |
| 1.01E-46 | PBP1a | mfd, oprM, mecC, mecA, PBP1a, PBP1b, emrA | antibiotic target replacement protein; beta-lactam resistance gene |
| 4.76E-75 | tetJ | tetY, tetZ, tetE, tet42, tet41, bmr, tetH, tetJ, mdtG, tet31, tet39, tetA, tetB, tet33, tetG, tet30 | efflux pump conferring antibiotic resistance; tetracycline resistance gene |
| 0 | tolC | tolC | chloramphenicol resistance gene; macrolide resistance gene; fluoroquinolone resistance gene; efflux pump conferring antibiotic resistance; aminocoumarin resistance gene; tetracycline resistance gene; rifampin resistance gene; beta-lactam resistance gene |
| 5.89E-80 | emrR | emrR, mgrA, mexQ | efflux pump conferring antibiotic resistance; gene modulating antibiotic efflux; fluoroquinolone resistance gene |
| 9.17E-14 | vanHB | vanSB, BcII, TEM-216, IND-4, vanHB, vanHA, vanHF, DIM-1, vanHD, SLB-1, vanHM, vanHO, EBR-1 beta-lactamase | glycopeptide resistance gene; antibiotic resistance gene cluster, cassette, or operon; gene conferring antibiotic resistance via molecular bypass |
| 3.80E-25 | macB | srmB, otrC, novA, mel, lsaA, vgaD, vgaALC, lsaC, salA, lsaB, msrE, vgaB, lsaE, msrC, oleB, oleC, lmrD, lmrC, vgaA, tlrC, msrA, sav1866, bcrA, vgaE, macB, carA | efflux pump conferring antibiotic resistance; macrolide resistance gene |
| 4.50E-35 | PBP2x | Streptomyces rishiriensis parY mutant conferring resistance to aminocoumarin, PBP2b, mecC, mecB, mecA, OXA-165, PBP1a, PBP1b, ANT(6)-Ia, aad(6), PBP2x, QnrA1, OXA-184 | antibiotic target replacement protein; beta-lactam resistance gene |
| 2.93E-113 | Streptomyces rishiriensis parY mutant conferring resistance to aminocoumarin | LRA-13, aadA, oleR, Streptomyces rishiriensis parY mutant conferring resistance to aminocoumarin, aadA25, aadA8, aadA2, aadA3, adeH, vanN | gene involved in self resistance to antibiotic; aminocoumarin resistance gene; antibiotic resistant gene variant or mutant |
| 1.43E-17 | lmrC | srmB, OXA-46, otrC, novA, mel, lsaA, vgaD, vgaALC, lsaC, dfrG, salA, lsaB, msrE, vgaB, lsaE, msrC, oleB, oleC, lmrD, lmrC, vgaA, tlrC, msrA, sav1866, bcrA, vgaE, macB, carA | efflux pump conferring antibiotic resistance; lincosamide resistance gene |
| 6.03E-109 | sav1866 | srmB, FomB, otrC, novA, mel, vanTG, FosA, salA, vgaD, vgaALC, lsaC, lsaA, lsaB, msrE, vgaB, lsaE, msrC, oleB, oleC, lmrD, lmrC, vgaA, tlrC, sav1866, ceoB, bcrA, vgaE, macB, carA | efflux pump conferring antibiotic resistance |
| 6.71E-15 | macB | srmB, FomB, otrC, novA, mel, lsaA, vgaD, vgaALC, msrE, salA, lsaB, lsaC, vgaB, lsaE, msrC, oleB, oleC, SPM-1, lmrD, lmrC, vgaA, tlrC, msrA, sav1866, bcrA, vgaE, macB, carA, vanSI | efflux pump conferring antibiotic resistance; macrolide resistance gene |
| 1.43E-11 | fyuA | mel, mexD, fyuA | tetracycline resistance gene; aminoglycoside resistance gene |
| 1.32E-29 | bcr-1 | emrY, tet42, otrB, tet43, tet41, norB, cmlB1, qacB, qacA, emrD, tetC, vanTG, mdfA, blt, emeA, tetG, tet39, pmrA efflux pump, tet33, tet31, tet30, tetY, floR, rosA, cmlA6, tetH, cmlA4, cmlA5, mdtG, tetL, cmlA1, tetA, tcr3, tetB, tetE, lmrB, facT, mdtL, tetK, bmr, pp-flo, cmlA, cmlB, norA, vanHB, mdtM, qepA, tcmA, bcr-1, tetJ | efflux pump conferring antibiotic resistance |
| 1.05E-06 | fyuA | oprA, IMP-44, IMP-11, IMP-41, fyuA, IMP-22, IMP-21 | tetracycline resistance gene; aminoglycoside resistance gene |
| 1.42E-16 | macB | srmB, novA, mel, lsaA, vgaALC, lsaC, salA, vgaD, msrE, vgaB, lsaE, msrC, oleB, oleC, lmrD, lmrC, vgaA, tlrC, sav1866, bcrA, vgaE, macB, carA | efflux pump conferring antibiotic resistance; macrolide resistance gene |
| 0 | Escherichia coli mutant GlpT conferring resistance to fosfomycin | Escherichia coli mutant GlpT conferring resistance to fosfomycin | fosfomycin resistance gene; antibiotic resistant gene variant or mutant |
| 1.84E-12 | evgA | evgA, PmrB, sul3, gadE, AAC(6')-IIc, sdiA | gene modulating antibiotic efflux; macrolide resistance gene; fluoroquinolone resistance gene; efflux pump conferring antibiotic resistance; tetracycline resistance gene; beta-lactam resistance gene |
| 1.56E-15 | mdtN | mexH, emrK, acrE, emrA, mtrC, smeA, mdtN, cmeA, mexX, mexV, TriA, amrA, TriB, adeF, mexM, mexJ, mdtE, mexE, mexC, mexA, mdtA, macA | efflux pump conferring antibiotic resistance |
| 1.40E-40 | mdtO | dfrA10, tetE, mdtO, mtrR | efflux pump conferring antibiotic resistance |
| 1.98E-19 | bcrA | srmB, otrC, novA, mel, lsaA, vgaD, vgaALC, msrE, salA, lsaB, lsaC, vgaB, lsaE, msrC, oleB, oleC, lmrD, lmrC, vgaA, tlrC, msrA, sav1866, bcrA, vgaE, macB, carA | efflux pump conferring antibiotic resistance; peptide antibiotic resistance gene |
| 3.22E-07 | dfrA3 | dfrA19, dfrA20, dfrA14, dfrA15, dfrA16, dfrA17, dfrA12, dfrA13, dfrA22, dfrD, mdfA, dfrG, dfrA8, mdsC, dfrA3, dfrA1, dfrA7, dfrA5, dfrA21, mtrD, dfrC, dfrA25, dfrA24, dfrF, dfrA26, dfrK, IND-9 | antibiotic target replacement protein; trimethoprim resistance gene |
| 3.16E-08 | blt | mefE, tet42, tet43, tet40, tet41, norB, norA, qacB, qacA, emrD, emrB, blt, emeA, qepA, tetG, tet39, pmrA efflux pump, tet33, tet31, cmrA, tetY, dfrC, floR, mdtM, bmr, tetH, cmlA4, cmlA5, mdtG, tetL, cmlA1, tetA, tetB, tetE, facT, mdtL, tetK, cmlA6, pp-flo, cmlA, cmlB, cmlB1, tetJ, cmx, bcr-1, Streptococcus agalactiae mprF | efflux pump conferring antibiotic resistance; fluoroquinolone resistance gene |
| 2.10E-38 | oleC | srmB, OXA-46, otrC, novA, OXA-119, msrA, lmrC, OXA-205, mel, lsaB, vgaALC, lsaC, lsaE, vgaD, salA, mexT, msrE, vgaB, msrC, lsaA, vgaA, OXA-3, oleB, oleC, lmrD, Bacillus Cluster A intrinsic mph, tlrC, OpmH, sav1866, OXA-21, bcrA, vgaE, macB, carA | efflux pump conferring antibiotic resistance |
| 1.49E-05 | mfd | mfd, dfrE, novA, bcrA, mexI, Bacillus Cluster A intrinsic mph | antibiotic target protection protein; fluoroquinolone resistance gene |
| 1.63E-20 | carA | srmB, FomB, otrC, novA, mel, salA, vgaD, vgaALC, lsaC, lsaA, lsaB, msrE, vgaB, lsaE, ErmD, msrC, oleB, oleC, lmrD, lmrC, vgaA, tlrC, msrA, sav1866, bcrA, vgaE, macB, carA | efflux pump conferring antibiotic resistance; macrolide resistance gene |
| 1.99E-47 | rifampin phosphotransferase | rifampin phosphotransferase, AAC(2')-Ic, AAC(2')-Ib | rifampin resistance gene; antibiotic inactivation enzyme |
| 1.82E-06 | macB | tlrC, srmB, lsaC, sav1866, otrC, mel, vgaD, msrE, vgaB, msrC, novA, bcrA, oleB, macB, lmrD, lsaA, carA, mdtO, salA, lmrC, oleC | efflux pump conferring antibiotic resistance; macrolide resistance gene |
| 8.63E-20 | bcrC | bcrC, Mycobacterium tuberculosis mutant embC conferring resistance to ethambutol | peptide antibiotic resistance gene; gene conferring antibiotic resistance via molecular bypass |
| 3.19E-30 | vanHA | vanHB, vanHA, vanHF, vanHD, vanHO, vanHM | glycopeptide resistance gene; antibiotic resistance gene cluster, cassette, or operon; gene conferring antibiotic resistance via molecular bypass |
| 4.34E-41 | cmlv | otrB, tet41, norA, blt, emeA, fexA, facT, tet39, tet31, cmrA, tetZ, tetH, cmlv, tetJ, mdtG, tetL, tetA, tcr3, tetB, lmrB, mdtM, cml, Brucella suis mprF, qepA, cmx, cmr | chloramphenicol resistance gene; antibiotic inactivation enzyme |
| 8.56E-05 | Mycobacterium tuberculosis murA | mtrE, oprJ, AAC(6')-If, IND-5, OXA-251, Chlamydia trachomatis murA, QnrVC7, QnrVC4, QnrVC5, lsaE, IND-9, CGB-1 beta-lactamase, Mycobacterium tuberculosis murA, IND-14 | fosfomycin resistance gene |
| 3.93E-14 | arlR | evgA, baeR, evgS, acrE, vanRA, vanRB, vanRC, vanRD, vanRE, vanRF, vanRG, vanRI, vanRL, vanRM, vanRN, vanRO, mtrA, adeR, arlR, cpxR, MOX-9, kdpE, smeR | efflux pump conferring antibiotic resistance; gene modulating antibiotic efflux; fluoroquinolone resistance gene |
| 5.52E-34 | PmrB | cpxA, adeS, PmrB, arlS, baeS, AAC(6')-30/AAC(6')-Ib' fusion protein, vanSD, vanSG, vanSF, vanSE, evgS, vanSC, vanSB, vanSA, vanSO, vanSN, vanSM, vanSL, smeS, vanSI | polymyxin resistance gene; gene altering cell wall charge conferring antibiotic resistance |
| 5.70E-24 | golS | mexN, leuO, golS, mel | efflux pump conferring antibiotic resistance; chloramphenicol resistance gene; beta-lactam resistance gene; gene modulating antibiotic efflux |
| 6.82E-06 | msrC | srmB, FomB, mel, lsaA, vgaD, vgaALC, msrE, salA, lsaB, lsaC, vgaB, msrC, lsaE, oleB, oleC, vgaA, lmrC, tlrC, msrA, vgaE, bcrA, macB, carA | efflux pump conferring antibiotic resistance; streptogramin resistance gene; macrolide resistance gene |
| 0 | macB | macB | efflux pump conferring antibiotic resistance; macrolide resistance gene |
| 2.17E-48 | abeM | mdtK, mdsB, abeM, OXA-62 | efflux pump conferring antibiotic resistance |
| 1.21E-55 | vanRI | evgA, baeR, evgS, vanRB, vanRA, dfrA8, vanRC, vanRD, vanRE, vanRF, vanRG, vanRI, vanRL, vanRM, vanRN, vanRO, mtrA, adeR, arlR, cpxR, kdpE, smeR | glycopeptide resistance gene; antibiotic resistance gene cluster, cassette, or operon; gene conferring antibiotic resistance via molecular bypass |
| 2.09E-15 | arlR | evgA, mtrA, adeR, baeR, vanRM, vanRL, arlR, smeR, cpxR, vanRA, vanRB, evgS, vanRD, vanRE, vanRF, vanRG, vanRI, kdpE, vanRC, vanRN, vanRO | efflux pump conferring antibiotic resistance; gene modulating antibiotic efflux; fluoroquinolone resistance gene |
| 4.63E-112 | robA | marA, ramA, mdtB, AAC(6')-Ir, tetD, robA | chloramphenicol resistance gene; gene modulating antibiotic efflux; fluoroquinolone resistance gene; efflux pump conferring antibiotic resistance; tetracycline resistance gene; rifampin resistance gene; beta-lactam resistance gene |
| 2.90E-25 | evgA | evgA, baeR, evgS, vanRA, vanRB, vanRC, vanRD, vanRE, vanRF, vanRG, vanRI, vanRL, vanRM, vanRN, vanRO, mtrA, adeR, arlR, cpxR, kdpE, sdiA, smeR | gene modulating antibiotic efflux; macrolide resistance gene; fluoroquinolone resistance gene; efflux pump conferring antibiotic resistance; tetracycline resistance gene; beta-lactam resistance gene |
| 7.78E-19 | adiY | adiY, bcrA, gadW, gadX, robA | efflux pump conferring antibiotic resistance |
| 1.08E-28 | otrA | tetB(P), Streptomyces rishiriensis parY mutant conferring resistance to aminocoumarin, cat-TC, tetS, tet44, tetT, otrA, cat, tetQ, Streptomyces cinnamoneus EF-Tu mutants conferring resistance to elfamycin, tetM, tetW, tetO, tet36, APH(3')-Ia, Escherichia coli EF-Tu mutants conferring resistance to kirromycin, tet32, vanN | antibiotic target protection protein; tetracycline resistance gene |
| 5.31E-21 | emrK | mdtE, mdsA, emrK, acrE, emrA, adeI, mexX, smeD, smeA, mdtN, cmeA, mtrC, amrA, TriB, adeF, mexM, adeA, mexH, mexE, mexC, mexA, acrA | efflux pump conferring antibiotic resistance; tetracycline resistance gene |
| 3.27E-11 | Bifidobacteria intrinsic ileS conferring resistance to mupirocin | mdtC, Bifidobacteria intrinsic ileS conferring resistance to mupirocin, smeB | mupirocin resistance gene |
| 5.74E-30 | dfrA3 | dfrA19, dfrA14, dfrA15, dfrA16, dfrA17, dfrA12, dfrA13, dfrA22, dfrA25, dfrG, dfrA8, dfrA3, dfrA1, dfrA7, dfrA5, dfrA21, dfrA20, dfrA23, dfrC, dfrD, dfrA24, dfrF, dfrA26, dfrK, Clostridium perfringens mprF, oleD, mgt | antibiotic target replacement protein; trimethoprim resistance gene |
| 1.90E-13 | QnrA4 | QnrB38, lnuC, QnrB32, QnrB33, QnrB30, QnrB31, QnrB36, QnrB37, QnrB34, QnrB35, QnrB29, QnrB28, QnrB21, QnrB20, QnrB23, QnrB22, QnrB25, QnrB24, QnrB27, QnrB26, DIM-1, QnrVC6, QnrVC7, QnrVC4, QnrVC5, QnrVC3, QnrVC1, QnrB14, QnrB15, QnrB16, QnrB17, QnrB10, QnrB11, QnrB12, QnrB13, QnrB18, QnrB19, iri, mfpA, QnrB74, QnrB72, QnrB73, QnrB70, QnrB71, QnrD1, QnrD2, QnrS1, QnrS3, QnrS2, QnrS5, QnrS4, QnrS7, QnrS6, QnrS9, QnrS8, QnrB65, QnrB64, QnrB67, QnrB66, QnrB61, QnrB60, QnrB62, QnrC, QnrB69, QnrB68, QnrB50, QnrB54, QnrB55, QnrB56, QnrB57, QnrB58, QnrB59, QnrB2, QnrB3, QnrB1, QnrB6, QnrB7, QnrB4, QnrB5, QnrB8, QnrB9, QnrB49, QnrB48, QnrB47, QnrB46, QnrB45, QnrB44, QnrB43, QnrB42, QnrB41, QnrB40, QnrA7, QnrA6, QnrA5, QnrA4, QnrA3, QnrA2, QnrA1 | antibiotic target protection protein; fluoroquinolone resistance gene |
| 2.14E-10 | mel | srmB, ANT(2'')-Ia, novA, mel, lmrD, lsaA, lsaB, vgaALC, lsaC, PBP2b, mexV, salA, vgaD, msrE, vgaB, msrC, lsaE, oleB, oleC, vgaA, lmrC, tlrC, msrA, sav1866, vanWI, bcrA, vgaE, macB, carA, cmr | efflux pump conferring antibiotic resistance; streptogramin resistance gene; macrolide resistance gene |
| 0.000615909 | tetT | tetM, oleR, tetT, tetW, fyuA | antibiotic target protection protein; tetracycline resistance gene |
| 0.300065 | Erm(30) | Erm(30) | antibiotic target modifying enzyme; lincosamide resistance gene; macrolide resistance gene; streptogramin resistance gene |
| 4.70E-07 | facT | Enterococcus faecium adeC, otrB, tetK, qepA, tcmA, tet39, qacB, qacA, tcr3, facT | efflux pump conferring antibiotic resistance; elfamycin resistance gene |
| 6.65E-45 | tcmA | emrY, mefE, mdtD, tet42, rosA, tet41, norB, cmlB1, qacB, emrB, emrD, qacA, mdtH, tet43, mdfA, blt, emeA, fexA, tetG, tet39, tet38, pmrA efflux pump, tet33, tet31, tet30, tetY, tetZ, AAC(6')-33, floR, tetC, otrB, bmr, tetH, cmlA4, cmlA5, mdtG, tetL, cmlA1, tetA, tcr3, tetB, tetE, lmrB, facT, mdtL, tetK, cmlA6, pp-flo, cmlA, cmlB, tet45, norA, mdtM, qepA, tcmA, cmx, bcr-1, cmrA, cmr, tetJ | efflux pump conferring antibiotic resistance |
| 1.87E-68 | mfd | AAC(3)-Xa, mfd, mexA | antibiotic target protection protein; fluoroquinolone resistance gene |
| 7.72E-43 | PBP1a | Erm(39), PBP1a, PBP1b, OXA-96, OXA-97, OXA-309, OXA-397, AAC(3)-IXa, PBP2b, OXA-58, OXA-231, mdtE, pp-flo, PBP2x, Erm(38), OXA-253, OXA-333, OXA-212, OXA-211, OXA-143, OXA-164, OXA-420 | antibiotic target replacement protein; beta-lactam resistance gene |
| 2.38E-35 | cmlv | OXA-3, cmlA1, norB, cmlB1, acrA, OXA-226, OXA-119, OXA-118, qacB, qacA, emrD, qepA, emeA, OXA-205, OXA-32, OXA-34, fexA, OXA-37, OXA-415, tet39, tet33, tet30, tetZ, OXA-53, floR, OXA-15, cmlA6, OXA-2, cmlA5, cmlA4, mdtD, tcr3, facT, tetK, cml, pp-flo, cmlA, cmlB, OXA-161, OXA-210, cmlv, OXA-141, OXA-21, OXA-20, tcmA, cmx, bcr-1, cmrA, cmr | chloramphenicol resistance gene; antibiotic inactivation enzyme |
| 0 | adeG | mtrD, adeB, ceoB, mdsB, smeE, mexY, smeB, amrB, mexQ, acrB, adeG, acrF, mdtF, acrD, mexF, mexD, mexB, cmeB, adeJ | efflux pump conferring antibiotic resistance; tetracycline resistance gene; fluoroquinolone resistance gene |
| 1.00E-13 | aminocoumarin resistant cysB | NmcR, aminocoumarin resistant cysB | aminocoumarin resistance gene |
| 3.57E-08 | robA | oleD, carA, AAC(3)-Ib, robA | chloramphenicol resistance gene; gene modulating antibiotic efflux; fluoroquinolone resistance gene; efflux pump conferring antibiotic resistance; tetracycline resistance gene; rifampin resistance gene; beta-lactam resistance gene |
| 8.26E-42 | robA | marA, TLA-3, TLA-1, tetD, evgS, gadX, dfrA3, Salmonella serovars soxS mutants, ramA, robA | chloramphenicol resistance gene; gene modulating antibiotic efflux; fluoroquinolone resistance gene; efflux pump conferring antibiotic resistance; tetracycline resistance gene; rifampin resistance gene; beta-lactam resistance gene |
| 2.31E-11 | sav1866 | srmB, vgaALC, novA, mel, lsaA, lsaB, MSI-1, lsaC, salA, vgaD, msrE, vgaB, lsaE, msrC, oleB, oleC, lmrD, lmrC, vgaA, tlrC, msrA, sav1866, bcrA, macB, carA | efflux pump conferring antibiotic resistance |
| 3.36E-21 | bcrA | srmB, otrC, novA, mel, lmrD, salA, lsaB, vgaALC, lsaC, mexY, lsaA, vgaD, msrE, vgaB, msrC, amrB, lsaE, oleB, oleC, vgaA, lmrC, tlrC, msrA, sav1866, bcrA, vgaE, macB, carA | efflux pump conferring antibiotic resistance; peptide antibiotic resistance gene |
| 0 | adeG | mtrD, adeB, ceoB, mdsB, smeE, mexY, smeB, amrB, mexQ, acrB, adeG, acrF, mdtF, acrD, mexF, mexD, mexB, cmeB, adeJ | efflux pump conferring antibiotic resistance; tetracycline resistance gene; fluoroquinolone resistance gene |
| 2.32E-33 | tetD | dfrA3, marA, TLA-3, APH(6)-Ic, TLA-1, tetD, evgS, CMY-73, OXA-9, CMY-49, gadW, Salmonella serovars soxS mutants, ramA, robA | efflux pump conferring antibiotic resistance; tetracycline resistance gene |
| 6.18E-06 | NmcR | tetA(P), leuO, mexT, AAC(3)-IIb, NmcR, aminocoumarin resistant cysB | antibiotic inactivation enzyme; beta-lactam resistance gene; gene modulating beta-lactam resistance |
| 2.79E-13 | nalD | acrS, nalD, APH(9)-Ia, mexL, mtrR, adeN | chloramphenicol resistance gene; gene modulating antibiotic efflux; trimethoprim resistance gene; macrolide resistance gene; fluoroquinolone resistance gene; efflux pump conferring antibiotic resistance; aminocoumarin resistance gene; tetracycline resistance gene; polymyxin resistance gene; beta-lactam resistance gene |
| 2.66E-30 | mdtL | emrY, tet43, cmlB1, qacB, emrB, emrD, qacA, mdfA, blt, qepA, tet39, salA, tet30, tetY, floR, cmlA6, cmlA4, cmlA5, tetL, cmlA1, tetA, adeN, tetB, mdtM, mdtL, pp-flo, cmlA, cmlB, tetK, tetJ, tcmA, bcr-1 | efflux pump conferring antibiotic resistance |
| 1.89E-25 | vanHB | tetX, rgt1438, vanHB, vanHA, vanHF, oleC, vanHD, vanHO, vanHM | glycopeptide resistance gene; antibiotic resistance gene cluster, cassette, or operon; gene conferring antibiotic resistance via molecular bypass |
| 3.46356 | acrF | acrF | efflux pump conferring antibiotic resistance; beta-lactam resistance gene; fluoroquinolone resistance gene |
| 1.04E-13 | sav1866 | OXA-55, novA, sav1866 | efflux pump conferring antibiotic resistance |
| 8.56E-11 | arnA | Bifidobacteria intrinsic ileS conferring resistance to mupirocin, arnA, LRA-12, PEDO-1 | polymyxin resistance gene; gene altering cell wall charge conferring antibiotic resistance |
| 2.75E-40 | sav1866 | srmB, otrC, novA, mel, lsaA, vgaD, vgaALC, lsaC, salA, lsaB, msrE, vgaB, lsaE, msrC, oleB, oleC, lmrD, lmrC, vgaA, tlrC, msrA, sav1866, bcrA, vgaE, macB, carA | efflux pump conferring antibiotic resistance |
| 6.14E-27 | novA | srmB, otrC, rosB, mel, lmrD, salA, vgaD, vgaALC, msrE, lsaA, lsaB, lsaC, vgaB, msrC, lsaE, oleB, novA, vgaA, lmrC, tlrC, msrA, sav1866, bcrA, vgaE, macB, carA, oleC | efflux pump conferring antibiotic resistance; aminocoumarin resistance gene |
| 9.93E-05 | tetD | marA, adiY, tetD, APH(6)-Ic, OXA-60, gadX, ramA, robA | efflux pump conferring antibiotic resistance; tetracycline resistance gene |
| 1.81E-23 | mdtL | tap, tet42, otrB, tet41, cmlB1, emrD, tetC, mdfA, blt, emeA, qepA, mdtM, tet39, tet31, SHV-67, floR, bmr, tetH, cmlA4, cmlA5, cmlA1, tetA, tcr3, tetB, tetE, tetG, mdtL, cmlA6, pp-flo, cmlA, cmlB, norA, adeG, tcmA, bcr-1, tetJ | efflux pump conferring antibiotic resistance |
| 2.35E-19 | oleC | srmB, FomB, otrC, novA, mel, lsaA, vgaD, vgaALC, lsaC, AAC(3)-IXa, salA, lsaB, msrE, vgaB, lsaE, msrC, oleB, oleC, lmrD, lmrC, vgaA, tlrC, msrA, sav1866, bcrA, vgaE, macB, carA | efflux pump conferring antibiotic resistance |
| 5.58E-50 | salA | srmB, otrC, novA, mel, lmrD, aminocoumarin resistant alaS, lsaA, cmeC, vgaD, vgaALC, lsaC, salA, lsaB, msrE, vgaB, msrC, lsaE, oleB, oleC, vgaA, lmrC, tlrC, msrA, sav1866, bcrA, vgaE, macB, LRA-3, carA | efflux pump conferring antibiotic resistance; lincosamide resistance gene; streptogramin resistance gene; pleuromutilin resistance gene |
| 8.79E-115 | mexA | mexH, mdsA, acrA, amrA, acrE, emrA, mexC, mtrC, smeD, SHV-105, smeA, adeI, cmeA, SHV-32, SHV-45, mexX, mexV, SHV-27, mexP, TriB, adeF, mexM, mexJ, adeA, mdtE, mexE, mdtN, mexA, ceoA, SHV-93, mdtA, emrK, macA | chloramphenicol resistance gene; trimethoprim resistance gene; macrolide resistance gene; fluoroquinolone resistance gene; efflux pump conferring antibiotic resistance; aminocoumarin resistance gene; tetracycline resistance gene; polymyxin resistance gene; beta-lactam resistance gene |
| 1.48E-09 | PmrF | Erm(36), PmrF | polymyxin resistance gene; gene altering cell wall charge conferring antibiotic resistance |
| 4.51E-06 | AAC(6')-33 | AAC(2')-Ia, tet42, AAC(6')-I30, sat-4, sat-3, lnuB, AAC(6')-Iai, AAC(6')-Iak, AAC(6')-Iaj, dfrC, AAC(6')-33, AAC(6')-Iih, AAC(1), AAC(6')-Ia, AAC(6')-Ii, linB, AAC(6')-Iu, AAC(6')-Ip, AAC(6')-Is, AAC(3)-Ia, arr-3, arr-2, AAC(6')-Iz | antibiotic inactivation enzyme; aminoglycoside resistance gene |
| 3.54E-10 | mtrR | nalD, acrS, mexL, MOX-5, mtrR | efflux pump conferring antibiotic resistance; gene modulating antibiotic efflux |
| 4.00E-12 | PmrC | catB, PmrC, CfxA6, MCR-1, OXA-118, OXA-119, BcII | polymyxin resistance gene; gene altering cell wall charge conferring antibiotic resistance |
| 1.72E-16 | oprJ | mtrE, oprJ, OpmH, mdtP, adeH, oprN, oprM, smeF, oprA, smeC, opmD, opmE, OXA-324, OXA-329, mdsC, tolC, cmeC, adeK | chloramphenicol resistance gene; beta-lactam resistance gene; macrolide resistance gene; fluoroquinolone resistance gene; efflux pump conferring antibiotic resistance; trimethoprim resistance gene |
| 1.39E-05 | TEM-178 | PBP1b, OKP-B-19, TEM-92, TEM-93, TEM-90, TEM-91, TEM-96, TEM-94, TEM-95, TEM-178, TEM-176, TEM-177, TEM-171, TEM-67, TEM-63, TEM-60, TEM-68, TEM-183, TEM-182, TEM-187, TEM-186, TEM-185, OKP-B-18, OKP-B-17, TEM-189, TEM-188, OKP-B-13, OKP-B-12, OKP-B-11, OKP-B-10, TEM-89, TEM-88, TEM-81, TEM-80, TEM-83, TEM-82, TEM-85, TEM-84, TEM-87, TEM-86, TEM-8, TEM-109, TEM-108, TEM-1, TEM-2, TEM-3, TEM-107, TEM-106, TEM-105, TEM-7, TEM-12, TEM-10, TEM-11, TEM-16, TEM-17, TEM-15, OKP-A-12, OKP-A-13, OKP-A-10, TEM-19, OKP-A-16, TEM-101, OKP-A-14, TEM-190, TEM-191, TEM-211, TEM-193, TEM-217, TEM-195, TEM-215, TEM-197, TEM-198, TEM-199, TEM-219, TEM-6, TEM-102, TEM-104, TEM-118, TEM-110, TEM-111, TEM-112, TEM-113, TEM-114, TEM-115, TEM-116, TEM-117, TEM-201, TEM-205, TEM-206, TEM-207, TEM-208, TEM-209, TEM-125, TEM-124, TEM-127, TEM-126, TEM-121, TEM-120, TEM-123, TEM-122, TEM-129, TEM-128, OKP-A-11, TEM-34, TEM-30, TEM-33, TEM-213, SHV-186, TEM-59, TEM-57, TEM-54, TEM-55, TEM-52, TEM-192, TEM-132, TEM-133, TEM-130, TEM-131, TEM-136, TEM-137, TEM-134, TEM-135, TEM-194, TEM-138, TEM-139, TEM-216, TEM-214, EreB, TEM-29, TEM-28, TEM-22, TEM-21, TEM-20, TEM-26, TEM-24, TEM-49, TEM-48, TEM-45, TEM-47, TEM-40, TEM-43, TEM-42, TEM-147, TEM-146, TEM-145, TEM-144, TEM-143, TEM-142, TEM-141, TEM-149, TEM-148, TEM-78, TEM-79, OKP-A-6, TEM-70, TEM-71, TEM-72, TEM-73, TEM-75, TEM-76, TEM-77, OKP-A-1, OKP-A-2, OKP-A-3, OKP-A-4, OKP-A-5, OKP-B-20, OKP-A-7, OKP-A-8, OKP-A-9, TEM-154, TEM-155, TEM-156, TEM-157, TEM-150, TEM-151, TEM-152, TEM-153, TEM-184, TEM-158, TEM-159, OKP-B-8, OKP-B-3, OKP-B-2, OKP-B-1, OKP-B-7, OKP-B-6, OKP-B-5, OKP-B-4, TEM-160, TEM-163, TEM-162, TEM-164, TEM-167, TEM-166, TEM-169, TEM-168, TEM-53 | antibiotic inactivation enzyme; beta-lactam resistance gene |
| 3.19E-05 | aminocoumarin resistant cysB | leuO, bleomycin resistance protein (BRP), SMB-1 beta-lactamase, aminocoumarin resistant cysB | aminocoumarin resistance gene |
| 1.38E-08 | aminocoumarin resistant cysB | leuO, NmcR, APH(3')-Vb, aminocoumarin resistant cysB | aminocoumarin resistance gene |
| 9.84E-10 | QnrD1 | QnrB38, QnrB32, QnrB33, QnrB30, QnrB31, QnrB36, QnrB37, QnrB34, QnrB35, QnrB29, QnrB28, QnrB21, QnrB20, QnrB23, QnrB22, QnrB25, QnrB24, QnrB27, QnrB26, QnrVC6, QnrVC7, QnrVC4, QnrVC5, QnrVC3, QnrVC1, QnrB14, QnrB15, QnrB16, QnrB17, QnrB10, QnrB11, QnrB12, QnrB13, QnrB18, QnrB19, mfpA, QnrB74, QnrB72, QnrB73, QnrB70, QnrB71, QnrD1, QnrD2, QnrS1, QnrS3, QnrS2, QnrS5, QnrS4, QnrS7, QnrS6, QnrS9, QnrS8, QnrB65, QnrB64, QnrB67, QnrB66, QnrB61, QnrB60, QnrB62, QnrC, QnrB69, QnrB68, cat, QnrB50, QnrB54, QnrB55, QnrB56, QnrB57, QnrB58, QnrB59, QnrB2, QnrB3, QnrB1, QnrB6, QnrB7, QnrB4, QnrB5, QnrB8, QnrB9, QnrB49, QnrB48, QnrB47, QnrB46, QnrB45, QnrB44, QnrB43, QnrB42, QnrB41, QnrB40, QnrA7, QnrA6, QnrA5, QnrA4, QnrA3, QnrA2, QnrA1 | antibiotic target protection protein; fluoroquinolone resistance gene |
| 4.08E-12 | aminocoumarin resistant cysB | leuO, srmB, vgaE, NmcR, aminocoumarin resistant cysB | aminocoumarin resistance gene |
| 7.65169 | ErmC | ErmC | antibiotic target modifying enzyme; lincosamide resistance gene; macrolide resistance gene; streptogramin resistance gene |
| 2.84E-47 | NmcR | leuO, mexT, NmcR, aminocoumarin resistant cysB, Enterococcus faecium adeC | antibiotic inactivation enzyme; beta-lactam resistance gene; gene modulating beta-lactam resistance |
| 1.60E-40 | mecC | PBP1a, OXA-114a, OXA-327, OXA-326, OXA-325, OXA-324, OXA-323, OXA-322, AAC(6')-Iaf, OXA-329, OXA-328, mepA, OXA-235, OXA-101, OXA-236, OXA-5, OXA-233, OXA-7, OXA-9, OXA-334, OXA-330, OXA-331, OXA-332, OXA-333, OXA-183, OXA-184, OXA-388, OXA-384, OXA-454, OXA-309, OXA-35, OXA-13, mecA, OXA-56, OXA-57, OXA-11, OXA-50, OXA-209, OXA-59, OXA-129, OXA-256, mecR1, OXA-251, OXA-28, OXA-42, OXA-134, OXA-246, OXA-240, OXA-363, OXA-362, OXA-360, LCR-1, mdtP, OXA-74, OXA-75, OXA-378, OXA-379, OXA-147, OXA-278, OXA-145, OXA-142, OXA-61, OXA-347, OXA-349, OXA-348, OXA-19, PBP2b, mecC, mecB, OXA-10, ErmA, OXA-17, OXA-14, OXA-352, OXA-353, OXA-350, OXA-351, OXA-357, OXA-354, OXA-358, OXA-359, PBP2x, OXA-213, OXA-212, OXA-211, OXA-237, OXA-215, OXA-214 | antibiotic resistance gene cluster, cassette, or operon; beta-lactam resistance gene; antibiotic target replacement protein |
| 0 | emrB | emrY, emrB | efflux pump conferring antibiotic resistance; fluoroquinolone resistance gene |
| 2.03E-34 | abeM | mdtK, abeM, vanN | efflux pump conferring antibiotic resistance |
| 6.80E-05 | vanD | vanA, vanC, vanE, vanD, vanI, vanO | glycopeptide resistance gene; antibiotic resistance gene cluster, cassette, or operon; gene conferring antibiotic resistance via molecular bypass |
| 4.42E-06 | fyuA | fyuA, MOX-9, PBP1b, tet40 | tetracycline resistance gene; aminoglycoside resistance gene |
| 7.26E-43 | oleC | srmB, otrC, novA, mel, lmrD, lsaA, vgaD, vgaALC, msrE, salA, lsaB, lsaC, vgaB, msrC, lsaE, oleB, oleC, vgaA, lmrC, tlrC, msrA, sav1866, bcrA, vgaE, macB, APH(4)-Ia, carA | efflux pump conferring antibiotic resistance |
| 2.94E-13 | fexA | tetA(P), tet45, tet42, otrB, PBP1b, tet41, cmlB1, tcr3, tetB, blt, fexA, tetG, tet39, pmrA efflux pump, tet31, tet30, tetY, tetZ, cmlv, tetJ, mdtG, tetL, tetA, mexD, mdtH, lmrB, facT, cml, tetK, AAC(3)-IIb, qepA, cmx, bcr-1, cmr | efflux pump conferring antibiotic resistance; phenicol resistance gene; chloramphenicol resistance gene |
| 3.81E-13 | vanSA | cpxA, adeS, PmrB, arlS, baeS, vanSD, vanSG, vanSF, vanSE, evgS, vanSC, vanSB, vanSA, mexI, vanSO, vanSN, vanSM, vanSL, smeS, vanSI | glycopeptide resistance gene; antibiotic resistance gene cluster, cassette, or operon; gene conferring antibiotic resistance via molecular bypass |
| 5.63E-06 | Bifidobacteria intrinsic ileS conferring resistance to mupirocin | AAC(6')-Ib-SK, vanE, TriC, Bifidobacteria intrinsic ileS conferring resistance to mupirocin | mupirocin resistance gene |
| 1.11E-08 | tsnr | QnrB70, QnrB22, QnrB28, QnrVC6, QnrB25, PBP1b, QnrB42, QnrB14, QnrB15, QnrB16, QnrB17, QnrB10, QnrB11, QnrB12, QnrB13, QnrB40, QnrB43, QnrB19, QnrS4, QnrB50, QnrB74, QnrB72, QnrB55, QnrB56, QnrB71, QnrB32, QnrB59, QnrB31, QnrB36, QnrB37, QnrB35, QnrB2, QnrB3, QnrB1, QnrB6, QnrB7, QnrB4, QnrB5, QnrB8, QnrB9, QnrS1, QnrB29, QnrS3, QnrS2, QnrS5, QnrB57, QnrS7, tlrC, QnrS9, QnrS8, QnrB73, TriA, QnrB27, QnrB24, QnrB38, QnrB48, QnrB54, QnrB66, tsnr, QnrB65, QnrB64, QnrB67, QnrB58, QnrB61, QnrB60, QnrB49, QnrB62, QnrB47, QnrB46, QnrB45, QnrB44, QnrB69, QnrB68, QnrB41, QnrB26, QnrB21, QnrS6, QnrB33, QnrB20, QnrB23, QnrB18 | antibiotic target modifying enzyme; peptide antibiotic resistance gene |
| 2.54E-07 | OpmH | vgaALC, oprM, OpmH, TriB, tolC, vgaA | efflux pump conferring antibiotic resistance; triclosan resistance gene |
| 1.24465 | tet42 | mecB, tet42 | efflux pump conferring antibiotic resistance; tetracycline resistance gene |
| 3.62E-73 | sav1866 | srmB, otrC, novA, mel, lsaA, vgaD, vgaALC, lsaC, salA, lsaB, msrE, vgaB, lsaE, msrC, oleB, oleC, lmrD, lmrC, vgaA, tlrC, sav1866, bcrA, vgaE, macB, carA | efflux pump conferring antibiotic resistance |
| 2.41E-11 | golS | y56 beta-lactamase, golS, MSI-1, ErmO | efflux pump conferring antibiotic resistance; chloramphenicol resistance gene; beta-lactam resistance gene; gene modulating antibiotic efflux |
| 3.88E-11 | PmrC | PmrC, MCR-1, NmcR | polymyxin resistance gene; gene altering cell wall charge conferring antibiotic resistance |
| 5.67E-13 | PmrE | QnrC, mexJ, PmrE | polymyxin resistance gene; gene altering cell wall charge conferring antibiotic resistance |
| 1.89E-45 | vanTrL | vanTG, LRA-13, vanTE, vanT, vanTN, vanTrL, vanTC | glycopeptide resistance gene; antibiotic resistance gene cluster, cassette, or operon; gene conferring antibiotic resistance via molecular bypass |
| 1.44E-125 | Streptomyces rishiriensis parY mutant conferring resistance to aminocoumarin | Escherichia coli parE conferring resistance to fluoroquinolones, vanSL, Streptomyces rishiriensis parY mutant conferring resistance to aminocoumarin | gene involved in self resistance to antibiotic; aminocoumarin resistance gene; antibiotic resistant gene variant or mutant |
| 1.56E-19 | macB | srmB, otrC, novA, mel, salA, vgaD, vgaALC, vanXI, lsaA, lsaB, lsaC, vgaB, lsaE, msrC, oleB, oleC, lmrD, lmrC, vgaA, tlrC, msrA, sav1866, adiY, msrE, bcrA, vgaE, macB, carA | efflux pump conferring antibiotic resistance; macrolide resistance gene |
| 8.44E-30 | oleC | srmB, otrC, novA, amrA, mel, lsaA, vgaD, vgaALC, lsaC, mexX, salA, mexT, msrE, vgaB, lsaE, msrC, oleB, oleC, lmrD, lmrC, vgaA, tlrC, msrA, sav1866, lsaB, bcrA, vgaE, macB, carA | efflux pump conferring antibiotic resistance |
| 2.90E-53 | clbB | clbC, clbB, clbA, mfd, vanSB, cfrA | linezolid resistance gene; macrolide resistance gene; antibiotic target modifying enzyme; phenicol resistance gene; lincosamide resistance gene; streptogramin resistance gene |
| 8.42E-09 | qacB | emrY, tet42, otrB, tet41, cmlB1, qacB, emrB, emrD, qacA, norA, mexE, mdfA, blt, emeA, fexA, qepA, tet39, pmrA efflux pump, tet33, tet31, tet30, tetY, tetZ, floR, cmlA6, tetH, cmlA4, cmlA5, mdtG, cmlA1, tetA, tetC, tetB, tetE, mdtM, mdtL, bmr, pp-flo, cmlA, cmlB, cmlv, bcrB, tetJ, tcmA, cmrA, cmr | efflux pump conferring antibiotic resistance; fluoroquinolone resistance gene |
| 1.21E-63 | H-NS | OpmH, mexM, H-NS, sat-4, mgt | gene modulating antibiotic efflux; macrolide resistance gene; fluoroquinolone resistance gene; efflux pump conferring antibiotic resistance; tetracycline resistance gene; beta-lactam resistance gene |
| 2.72E-06 | fyuA | fyuA, Mrx, vanRM | tetracycline resistance gene; aminoglycoside resistance gene |
| 3.68E-07 | adeN | mtrE, catB7, nalD, lsaC, acrS, cmeR, opmE, mexL, mtrR, adeN | chloramphenicol resistance gene; gene modulating antibiotic efflux; lincosamide resistance gene; macrolide resistance gene; fluoroquinolone resistance gene; efflux pump conferring antibiotic resistance; aminocoumarin resistance gene; tetracycline resistance gene; rifampin resistance gene; beta-lactam resistance gene; trimethoprim resistance gene |
| 1.88E-68 | Klebsiella pneumoniae acrR mutant resulting in high level antibiotic resistance | acrS, AAC(2')-Id, nalD, cmeR, mexL, Klebsiella pneumoniae acrR mutant resulting in high level antibiotic resistance, mtrR, adeN | chloramphenicol resistance gene; gene modulating antibiotic efflux; fluoroquinolone resistance gene; efflux pump conferring antibiotic resistance; antibiotic resistant gene variant or mutant; tetracycline resistance gene; rifampin resistance gene; beta-lactam resistance gene |
| 5.26E-60 | Bifidobacteria intrinsic ileS conferring resistance to mupirocin | Bifidobacteria intrinsic ileS conferring resistance to mupirocin | mupirocin resistance gene |
| 1.68E-13 | NmcR | leuO, mexT, vanYB, NmcR, aminocoumarin resistant cysB | antibiotic inactivation enzyme; beta-lactam resistance gene; gene modulating beta-lactam resistance |
| 2.10E-17 | fexA | emrY, tet45, tet42, otrB, tet41, norB, cmlB1, qacB, emrB, emrD, qacA, vanTG, mdfA, blt, fexA, qepA, tetG, tet39, tet38, pmrA efflux pump, tet33, tet31, tet30, tetY, floR, tetC, tet43, cmlA6, tetH, cmlv, tetJ, mdtM, tetL, mdtD, tcr3, tetE, lmrB, facT, mdtL, tetK, cml, pp-flo, cmlA, cmlB, cmlA1, norA, cmlA5, tcmA, cmx, bcr-1, cmrA, cmr, adeA | efflux pump conferring antibiotic resistance; phenicol resistance gene; chloramphenicol resistance gene |
| 1.47E-05 | AAC(6')-Ia | AAC(6')-Ia, AAC(6')-I30, CGB-1 beta-lactamase, sat-4, AAC(6')-Iae, AAC(6')-Iai, AAC(6')-Iak, AAC(6')-Iaj, mecI, dfrA22, AAC(6')-33, mdtB, AAC(6')-Ie-APH(2'')-Ia, AAC(1), IND-2a, IND-11, IND-10, IND-12, IND-15, IND-14, IND-1, IND-3, IND-2, IND-5, IND-4, IND-7, IND-6, IND-9, IND-8, AAC(6')-Ii, AAC(6')-Iq, AAC(6')-Ip, AAC(3)-Ia, AAC(6')-Iz | antibiotic inactivation enzyme; aminoglycoside resistance gene |
| 1.02E-48 | tetT | tetB(P), mefB, tet44, otrA, OXA-35, Streptomyces cinnamoneus EF-Tu mutants conferring resistance to elfamycin, tet36, tet32, OXA-56, OXA-19, tetQ, OXA-13, tetS, tetT, tetW, OXA-101, tetM, tetO, OXA-147, OXA-145, TLA-3, OXA-183, Escherichia coli EF-Tu mutants conferring resistance to kirromycin, OXA-28 | antibiotic target protection protein; tetracycline resistance gene |
| 4.41E-11 | cat | catB7, catB6, vatH, catB3, catB2, PmrC, catB9, catB8, vatC, vatB, vatA, qacH, vatF, vatE, vatD, cat, catB10 | chloramphenicol resistance gene; antibiotic inactivation enzyme |
| 8.13E-11 | golS | golS, PDC-8 | efflux pump conferring antibiotic resistance; chloramphenicol resistance gene; beta-lactam resistance gene; gene modulating antibiotic efflux |
| 2.47E-23 | mexS | EreB, mexJ, mexS, mgt | efflux pump conferring antibiotic resistance; chloramphenicol resistance gene; trimethoprim resistance gene; gene modulating antibiotic efflux; fluoroquinolone resistance gene |
| 4.19E-12 | arnA | arnA, vanO | polymyxin resistance gene; gene altering cell wall charge conferring antibiotic resistance |
| 2.38E-123 | mexH | mexV, mexH | efflux pump conferring antibiotic resistance; fluoroquinolone resistance gene |
| 3.74E-12 | tet32 | TEM-190, TEM-192, tetB(P), TEM-189, tet44, TEM-73, otrA, TEM-77, TEM-154, TEM-157, TEM-158, TEM-116, TEM-171, tetQ, Streptomyces cinnamoneus EF-Tu mutants conferring resistance to elfamycin, tet36, tet32, TEM-205, TEM-185, TEM-45, tetS, vgaE, tetT, tetW, TEM-125, TEM-108, tetM, tetO, TEM-81, TEM-80, TEM-83, TEM-109, TEM-162, TEM-169, Escherichia coli EF-Tu mutants conferring resistance to kirromycin, TEM-33 | antibiotic target protection protein; tetracycline resistance gene |
| 3.31E-07 | vanRF | baeR, PBP1b, evgS, emrA, vanRA, vanRC, vanRD, vanRE, vanRF, vanRG, vanRI, vanRL, vanRM, vanRN, mtrA, adeR, cpxR, IND-14, IND-3, CdeA, IND-5, IND-6, smeR, cat, EBR-1 beta-lactamase | glycopeptide resistance gene; antibiotic resistance gene cluster, cassette, or operon; gene conferring antibiotic resistance via molecular bypass |
| 3.20E-46 | Streptomyces cinnamoneus EF-Tu mutants conferring resistance to elfamycin | Streptomyces cinnamoneus EF-Tu mutants conferring resistance to elfamycin, adeB | gene involved in self resistance to antibiotic; antibiotic resistant gene variant or mutant; elfamycin resistance gene |
| 9.41E-20 | Bifidobacteria intrinsic ileS conferring resistance to mupirocin | Bifidobacteria intrinsic ileS conferring resistance to mupirocin, ErmU | mupirocin resistance gene |
| 4.22E-16 | macB | Bifidobacteria intrinsic ileS conferring resistance to mupirocin, srmB, TEM-193, otrC, novA, CTX-M-151, mel, lsaA, lsaB, vgaALC, lsaC, salA, vgaD, msrE, vgaB, lsaE, msrC, y56 beta-lactamase, oleB, oleC, lmrD, lmrC, vgaA, tlrC, msrA, sav1866, bcrA, vgaE, macB, carA | efflux pump conferring antibiotic resistance; macrolide resistance gene |
| 7.79E-42 | abeM | AAC(6')-Ib10, AAC(6')-Ib11, vanTC, ANT(3'')-Ii-AAC(6')-IId fusion protein, AAC(6')-Ib', AAC(6')-Ib9, tet38, AAC(6')-Ib4, abeM, AAC(6')-IId, AAC(6')-Ib3, AAC(3)-Ib/AAC(6')-Ib'', AAC(6')-Ib-Hangzhou, AAC(6')-30/AAC(6')-Ib' fusion protein, tetV, mdtK, vanT, mphB, AAC(6')-Ib-Suzhou, AAC(6')-Ib, AAC(6')-Ib-cr, vanN | efflux pump conferring antibiotic resistance |
| 9.76E-89 | sav1866 | srmB, otrC, novA, mel, smeB, lsaA, vgaD, vgaALC, vgaE, salA, lsaB, lsaC, vgaB, lsaE, msrC, oleB, oleC, lmrD, lmrC, vgaA, tlrC, msrA, sav1866, bcrA, macB, carA | efflux pump conferring antibiotic resistance |
| 3.37E-05 | sat-4 | Erm(39), AAC(6')-I30, sat-4, sat-3, sat-1, AAC(6')-Iaa, AAC(6')-Iak, AAC(6')-33, AAC(6')-Ig, AAC(6')-If, AAC(6')-Ic, AAC(6')-Ih, AAC(6')-Ik, AAC(6')-Iu, AAC(6')-It, AAC(6')-Iw, AAC(6')-Iv, AAC(6')-Is, AAC(6')-Ir, AAC(6')-Iy, AAC(6')-Ix, AAC(6')-Iz | streptothricin resistance gene; antibiotic inactivation enzyme |
| 3.31E-12 | baeS | vanSL, vanSD, baeS, PmrB, arlS, smeS, vanSG, vanSF, vanSE, evgS, vanSC, vanSB, vanSA, vanSO, vanSN, vanSM, dfrA1, lmrC, vanSI | efflux pump conferring antibiotic resistance; aminocoumarin resistance gene; aminoglycoside resistance gene; gene modulating antibiotic efflux |
| 2.41E-08 | mtrA | evgA, mtrA, adeR, baeR, arlR, smeR, cpxR, vanRA, evgS, vanRD, vanRE, vanRF, vanRG, vanRI, kdpE, vanRM | efflux pump conferring antibiotic resistance; gene modulating antibiotic efflux |
| 1.40E-07 | evgA | evgA, adeR, ErmB, smeE, arlR, IMI-3, cpxR, vanRA, vanRB, vanRC, acrF, mdtF, sdiA, vanRO, kdpE, adeJ, vanRE | gene modulating antibiotic efflux; macrolide resistance gene; fluoroquinolone resistance gene; efflux pump conferring antibiotic resistance; tetracycline resistance gene; beta-lactam resistance gene |
| 5.34E-05 | ErmB | vgaALC, ErmA, Streptomyces rishiriensis parY mutant conferring resistance to aminocoumarin, Erm(33), ErmB, ErmC, Erm(34), Erm(35), ErmD, vgaA, Erm(31), Erm(37), ErmY, Erm(42), cpxR, ErmE, ErmR, ErmQ, ErmF, ErmT | antibiotic target modifying enzyme; lincosamide resistance gene; macrolide resistance gene; streptogramin resistance gene |
| 4.71E-07 | norA | tap, tet41, norB, norA, qacB, qacA, mdfA, blt, emeA, fexA, tet39, tet33, cmrA, tetY, tetZ, bmr, cmlv, mdtG, tetL, mdtD, tetA, tcr3, tetB, tetE, lmrB, tetG, cml, cmx, cmr | efflux pump conferring antibiotic resistance; fluoroquinolone resistance gene |
| 9.93E-14 | bcrA | srmB, dfrA17, otrC, novA, mel, salA, lsaA, lsaB, vgaALC, lsaC, mexW, vgaD, msrE, vgaB, lsaE, msrC, oleB, oleC, lmrD, lmrC, vgaA, tlrC, msrA, sav1866, bcrA, vgaE, macB, carA | efflux pump conferring antibiotic resistance; peptide antibiotic resistance gene |
| 6.02E-22 | aminocoumarin resistant cysB | DHA-17, DHA-3, leuO, mexT, DHA-7, DHA-6, DHA-5, OXA-214, DHA-22, DHA-19, DHA-21, NmcR, OXA-215, aminocoumarin resistant cysB, DHA-13, DHA-12, DHA-14, OXA-349, DHA-1, DHA-15, DHA-20 | aminocoumarin resistance gene |
| 1.88E-15 | pmrA efflux pump | emrY, mefB, mefC, tet42, otrB, tet40, tet41, norB, norA, QnrS5, qacB, qacA, tcr3, QnrS1, emrB, mdtH, blt, emeA, fexA, tetG, tet39, pmrA efflux pump, tet33, tet31, tet30, tetY, QnrS3, tetZ, Mrx, QnrS4, QnrS7, QnrS9, QnrS8, mdtM, tetH, cmlv, tetJ, mdtG, tetL, tetA, tetC, tetB, tetE, facT, mdtL, tetK, AAC(6')-Ih, qepA, tcmA, bcr-1, cmrA | efflux pump conferring antibiotic resistance; fluoroquinolone resistance gene |
| 2.86E-05 | FosB3 | FosX, FosK, FosA, FosB, FosA2, FosA3, bleomycin resistance protein (BRP), FosA4, FosA5, FosC2, FosB3, tsnr | fosfomycin resistance gene; antibiotic inactivation enzyme |
| 7.61E-09 | tcmA | tetK, emrY, emrB, fexA, blt, emeA, rosA, tet43, bmr, norB, norA, bcr-1, tcmA, qacB, qacA, emrD, lmrB, tetG, tetL | efflux pump conferring antibiotic resistance |
| 2.13E-15 | aminocoumarin resistant cysB | mdsB, IMP-31, IMP-33, NmcR, IMP-35, aminocoumarin resistant cysB, IMP-11, IMP-41, IMP-44, IMP-21 | aminocoumarin resistance gene |
| 0 | mdtH | mdtH | efflux pump conferring antibiotic resistance |
| 3.40E-07 | emrE | ykkD, GIM-2, GIM-1, tmrB, qacH, qacB, qacA, emrE, abeS | efflux pump conferring antibiotic resistance |
| 4.31E-72 | bcr-1 | bcr-1 | efflux pump conferring antibiotic resistance |
| 6.84E-161 | baeS | baeS | efflux pump conferring antibiotic resistance; aminocoumarin resistance gene; aminoglycoside resistance gene; gene modulating antibiotic efflux |
| 2.70E-06 | vatA | catB7, catB6, vatH, catB3, catB2, vatF, catB9, catB8, vatC, vatB, vatA, desR, vatE, vatD, cat, tet32, catB10 | antibiotic inactivation enzyme; streptogramin resistance gene |
| 1.19E-15 | CMY-23 | ACT-35, LRA-13, ACT-15, LRA-18, CMY-29, CMY-99, CMY-98, CMY-93, CMY-90, CMY-95, CMY-94, CMY-13, CMY-12, CMY-11, CMY-10, CMY-17, CMY-16, CMY-15, CMY-14, DHA-3, DHA-2, DHA-1, CMY-18, DHA-7, DHA-6, DHA-5, FOX-5, FOX-4, FOX-7, CMY-76, FOX-1, FOX-3, FOX-2, FOX-9, FOX-8, ACT-6, ACT-7, ACT-4, ACT-5, ACT-2, ACT-3, CMY-28, ACT-1, OCH-6, ACT-9, CMY-31, MIR-8, MIR-9, CMY-30, MIR-2, MIR-3, MIR-1, MIR-6, MIR-4, MIR-5, CMY-66, CMY-67, CMY-64, CMY-65, CMY-62, CMY-63, CMY-60, CMY-61, DHA-13, DHA-12, DHA-10, DHA-17, DHA-16, CMY-68, CMY-69, ACC-1, ACC-3, ACC-2, ACC-5, ACC-4, CMY-6, OCH-4, OCH-1, MIR-14, MIR-15, MIR-16, MIR-17, MIR-10, MIR-11, MIR-12, MIR-13, CMY-75, CMY-74, CMY-77, DHA-18, CMY-71, CMY-70, CMY-73, CMY-72, CMY-79, CMY-78, CMY-23, CMY-2, CMY-1, CMY-7, ACT-16, CMY-5, CMY-4, CMY-9, CMY-8, MOX-2, MOX-3, MOX-1, MOX-6, MOX-7, MOX-4, MOX-5, MOX-8, MOX-9, DHA-15, OCH-3, FOX-10, ACT-13, CMY-26, ACT-36, CMY-41, CMY-42, CMY-43, CMY-44, CMY-45, ACT-30, CMY-47, CMY-48, CMY-49, ADC-2, DHA-14, CMY-19, ACT-31, ACT-25, ACT-24, ACT-27, ACT-21, ACT-20, CMY-59, ACT-22, CMY-57, CMY-56, CMY-55, CMY-54, CMY-53, ACT-28, CMY-51, CMY-50, CMY-39, ACT-23, CMY-58, CMY-119, CMY-118, CMY-113, CMY-112, CMY-111, CMY-110, CMY-117, CMY-116, CMY-115, CMY-114, LAT-1, ACT-29, ACT-10, OCH-5, ACT-12, OCH-7, ACT-14, DHA-19, OCH-2, ACT-17, ACT-18, ACT-19, CMY-20, CMY-21, OCH-8, CMY-27, CMY-24, CMY-25, CepS beta-lactamase, SRT-2, SRT-1, CMY-108, CMY-100, CMY-101, CMY-102, CMY-103, CMY-104, CMY-105, CMY-40, ACT-37, DHA-22, CMY-38, DHA-20, DHA-21, AQU-1, ACT-32, CMY-33, CMY-32, CMY-35, CMY-34, CMY-37, CMY-46, CMY-22, CFE-1, PDC-10, CMY-84, CMY-85, CMY-86, CMY-87, CMY-80, CMY-81, CMY-82, CMY-83, PDC-5, PDC-4, PDC-7, PDC-6, PDC-1, PDC-3, PDC-2, AER-1, PDC-9, PDC-8 | antibiotic inactivation enzyme; beta-lactam resistance gene |
| 1.14E-15 | vanRB | evgA, baeR, evgS, NDM-5, NDM-4, vanRA, vanRB, vanRC, vanRD, vanRE, vanRF, vanRG, vanRI, vanRL, vanRM, NDM-13, vanRO, mtrA, adeR, arlR, cpxR, mexD, kdpE, adeJ, vanTmL, smeR, vanRN, NDM-12 | glycopeptide resistance gene; antibiotic resistance gene cluster, cassette, or operon; gene conferring antibiotic resistance via molecular bypass |
| 1.42E-06 | fyuA | SHV-66, SHV-77, SHV-183, SHV-70, SHV-67, SHV-31, SHV-56, SHV-35, SHV-182, SHV-36, SHV-123, SHV-62, SHV-128, SHV-129, SHV-53, SHV-108, SHV-109, SHV-89, SHV-85, SHV-86, SHV-80, SHV-81, catB9, SHV-107, fyuA, SHV-2A, SHV-40, SHV-144, SHV-64, SHV-29, Mrx, SHV-60, SHV-61, SHV-79, SHV-173, SHV-69, SHV-134, SHV-158, SHV-157, SHV-156, SHV-155, SHV-172, SHV-93, SHV-178, SHV-110, SHV-96, SHV-159, SHV-94, SHV-13, SHV-12, SHV-11, SHV-15, SHV-92 | tetracycline resistance gene; aminoglycoside resistance gene |
| 6.24E-40 | tetT | MSI-1, tetB(P), tetQ, tetS, tetT, otrA, tet32, Streptomyces cinnamoneus EF-Tu mutants conferring resistance to elfamycin, tetM, tetW, tetO, tet36, tet44 | antibiotic target protection protein; tetracycline resistance gene |
| 5.18E-05 | sat-4 | sat-4, AAC(6')-If, AAC(6')-33, AAC(6')-Ic, AAC(6')-I30, CTX-M-151, AAC(1), sdiA, CTX-M-53, sat-1, sat-3, AAC(3)-Ia | streptothricin resistance gene; antibiotic inactivation enzyme |
| 1.26E-18 | macB | SRT-2, srmB, SRT-1, novA, mel, lmrD, lsaA, lsaB, vgaALC, lsaC, salA, vgaD, msrE, vgaB, msrC, lsaE, oleB, oleC, msrA, lmrC, vgaA, tlrC, sav1866, bcrA, vgaE, macB, carA | efflux pump conferring antibiotic resistance; macrolide resistance gene |
| 2.76E-26 | abeM | mdtK, mtrD, CdeA, abeM, mepA | efflux pump conferring antibiotic resistance |
| 3.28E-92 | mdtG | emrY, SFB-1, mefA, mefB, mdtD, tet42, tet43, otrA, tet41, norB, cmlB1, APH(3')-Ib, qacB, emrB, tetC, norA, mdtH, mdfA, blt, emeA, fexA, otrB, tetG, tet39, tet38, pmrA efflux pump, tet33, cmeB, tet31, tet30, tetY, tetZ, floR, SIM-1 beta-lactamase, rosA, cmlA6, tetH, cmlA4, tetJ, mdtG, tet40, cmlA1, tetA, tcr3, tetB, tetE, lmrB, facT, bmr, pp-flo, cmlA, cmlB, tetK, mdtM, cmlA5, qepA, tcmA, qacA, bcr-1, tetL | efflux pump conferring antibiotic resistance |
| 4.64E-05 | Enterococcus faecium adeC | Enterococcus faecium adeC, Brucella suis mprF | efflux pump conferring antibiotic resistance; tetracycline resistance gene |
| 1.87E-05 | vatB | catB7, evgA, vatH, catB3, catB2, catB6, QnrVC7, QnrVC4, QnrVC5, catB9, cat, vatC, vatB, vatF, vatE, vatD, vanSN, catB8, catB10 | antibiotic inactivation enzyme; streptogramin resistance gene |
| 1.77E-53 | vanHO | tetX, vanHB, vanHA, vanHF, vanHD, vanHO, vanHM | glycopeptide resistance gene; antibiotic resistance gene cluster, cassette, or operon; gene conferring antibiotic resistance via molecular bypass |
| 2.53E-09 | arnA | arnA, vanO | polymyxin resistance gene; gene altering cell wall charge conferring antibiotic resistance |
| 3.96246 | AAC(6')-Iq | smeS, OXA-192, AAC(6')-Iq, arlS | antibiotic inactivation enzyme; aminoglycoside resistance gene |
| 5.23E-20 | macB | srmB, otrC, PBP1b, mel, lmrD, msrA, salA, abeM, vgaD, vgaALC, msrE, lsaA, lsaB, lsaC, vgaB, msrC, lsaE, oleB, oleC, vgaA, lmrC, tlrC, novA, sav1866, bcrA, vgaE, macB, carA | efflux pump conferring antibiotic resistance; macrolide resistance gene |
| 1.45E-17 | aminocoumarin resistant cysB | catQ, NmcR, aminocoumarin resistant cysB, leuO | aminocoumarin resistance gene |
| 3.20E-32 | Streptomyces cinnamoneus EF-Tu mutants conferring resistance to elfamycin | tetB(P), tetQ, tetS, dfrA17, tetT, otrA, dfrA7, Streptomyces cinnamoneus EF-Tu mutants conferring resistance to elfamycin, tetM, tetW, tetO, tet36, mexG, tet32, tet44 | gene involved in self resistance to antibiotic; antibiotic resistant gene variant or mutant; elfamycin resistance gene |
| 7.78E-60 | sav1866 | srmB, otrC, novA, mel, lsaA, lsaB, vgaALC, lsaC, salA, vgaD, msrE, vgaB, lsaE, msrC, oleB, oleC, lmrD, lmrC, vgaA, tlrC, msrA, CdeA, sav1866, OXA-145, bcrA, vgaE, macB, carA | efflux pump conferring antibiotic resistance |
| 8.57E-28 | mdtL | tetK, Brucella suis mprF, emrD, mdtF, mdtL | efflux pump conferring antibiotic resistance |
| 9.45E-31 | Salmonella serovars soxS mutants | marA, vanRL, arlR, tetD, gadX, robA, adiY, gadW, Salmonella serovars soxS mutants, ramA, vanRN | chloramphenicol resistance gene; gene modulating antibiotic efflux; gene modulating permeability to antibiotic; fluoroquinolone resistance gene; efflux pump conferring antibiotic resistance; antibiotic resistant gene variant or mutant; tetracycline resistance gene; rifampin resistance gene; beta-lactam resistance gene |
| 4.66E-22 | macB | srmB, otrC, CRP, novA, mel, lmrD, lsaA, lsaB, vgaALC, lsaC, salA, vgaD, msrE, vgaB, msrC, lsaE, mdtC, oleB, oleC, vgaA, lmrC, tlrC, msrA, sav1866, bcrA, vgaE, macB, carA | efflux pump conferring antibiotic resistance; macrolide resistance gene |
| 8.50E-07 | mdtO | ACT-37, mdtO, ACT-19, mtrR | efflux pump conferring antibiotic resistance |
| 2.19E-08 | bcr-1 | mefB, RlmA(II), cmlB1, OXA-170, OXA-171, OXA-133, emrD, fexA, mdfA, OXA-366, OXA-435, OXA-73, floR, cmlA6, cmlv, cmlA5, cmlA1, mdtM, mdtL, OXA-169, pp-flo, cmlA, cmlB, Brucella suis mprF, cmlA4, OXA-167, OXA-166, OXA-23, OXA-27, OXA-422, OXA-423, bcr-1, vanJ | efflux pump conferring antibiotic resistance |
| 1.24113 | mexI | AAC(6')-Iai, CfxA6, srmB, mexI | efflux pump conferring antibiotic resistance; fluoroquinolone resistance gene |
| 3.14E-131 | Streptomyces cinnamoneus EF-Tu mutants conferring resistance to elfamycin | Streptomyces cinnamoneus EF-Tu mutants conferring resistance to elfamycin | gene involved in self resistance to antibiotic; antibiotic resistant gene variant or mutant; elfamycin resistance gene |
| 3.64E-54 | evgS | evgA, baeR, baeS, evgS, cpxA, cmeR, vanRA, vanRB, vanRC, vanRD, vanRE, vanRF, vanRG, vanRI, vanRL, smeR, vanRN, vanRO, mtrA, aminocoumarin resistant cysB, adeR, adeS, arlS, arlR, cpxR, kdpE, smeS, vanRM, vanSG, vanSF, vanSE, vanSD, vanSC, vanSB, vanSA, PmrB, vanSN, vanSM, vanSL, vanSI | gene modulating antibiotic efflux; macrolide resistance gene; fluoroquinolone resistance gene; efflux pump conferring antibiotic resistance; tetracycline resistance gene; beta-lactam resistance gene |
| 1.24E-35 | vanTG | vanTG, vanTE, vanT, vanTN, msrE, sul2, IMI-7, vanTrL, tetG, vanTC | glycopeptide resistance gene; antibiotic resistance gene cluster, cassette, or operon; gene conferring antibiotic resistance via molecular bypass |
| 2.06E-09 | mdtL | emrY, tap, mefA, cmlA1, tet42, tet43, tet41, norB, cmlB1, qepA, qacB, qacA, emrD, tetC, norA, mdtH, mdfA, blt, emeA, myrA, fexA, vanRC, vanRE, tet39, tet38, pmrA efflux pump, tet33, tet31, tet30, tetY, tetZ, Mrx, floR, otrB, cmlA6, tetH, cmlv, cmlA5, mdtG, tetL, mdtD, tetA, tcr3, tetB, tetE, lmrB, tetG, mdtL, tetK, bmr, pp-flo, cmlA, cmlB, cmlA4, mdtM, tetJ, tcmA, cmx, bcr-1, cmrA, cmr | efflux pump conferring antibiotic resistance |
| 1.248 | EreA2 | mdfA, EreA2, vanSD | antibiotic inactivation enzyme; macrolide resistance gene |
| 1.17E-29 | PBP1a | leuO, PBP1a, PBP1b, OXA-62, cepA beta-lactamase, lmrC | antibiotic target replacement protein; beta-lactam resistance gene |
| 1.07E-05 | emrB | emrY, emrB, QnrB37, QnrB60, QnrB21, QnrB25, QnrB69, tet43, QnrB35, norB, QnrB38, QnrB11, QnrB12, qacB, qacA, abeM, lmrB, QnrB8 | efflux pump conferring antibiotic resistance; fluoroquinolone resistance gene |
| 1.56E-46 | catB9 | catB7, catB6, catB3, catB2, catB9, cat, vatC, vatB, vatA, vatF, vatE, vatD, vatH, catB8, catB10 | chloramphenicol resistance gene; antibiotic inactivation enzyme |
| 2.65E-13 | macB | srmB, otrC, novA, mel, lsaA, lsaB, vgaALC, lsaC, salA, vgaD, msrE, vgaB, msrC, lsaE, oleB, oleC, vgaA, lmrC, tlrC, msrA, sav1866, bcrA, vgaE, macB, carA | efflux pump conferring antibiotic resistance; macrolide resistance gene |
| 4.51E-10 | mtrA | evgA, baeR, evgS, vanRA, vanRB, vanRC, vanRD, vanRE, vanRF, vanRG, vanRI, vanRL, vanRM, vanRN, vanRO, mtrA, adeR, arlR, cpxR, kdpE, OXA-85, smeR, bcrA | efflux pump conferring antibiotic resistance; gene modulating antibiotic efflux |
| 1.14E-25 | floR | emrY, mefB, mdtD, tet42, tet43, tet41, norB, norA, qacB, emrB, emrD, tetC, mdfA, blt, emeA, fexA, qepA, tetG, tet39, tet38, pmrA efflux pump, tet33, tet31, tet30, tetY, tetZ, floR, otrB, cmlA6, tetH, qacA, tetJ, mdtG, tetL, cmlA1, tetA, tcr3, tetB, tetE, lmrB, facT, mdtL, tetK, bmr, cml, pp-flo, cmlA, cmlB, tet45, cmlv, cmlA4, mdtM, cmlA5, cmlB1, tcmA, cmx, bcr-1, cmrA, cmr | efflux pump conferring antibiotic resistance; phenicol resistance gene; chloramphenicol resistance gene |
| 7.32E-49 | sav1866 | srmB, otrC, novA, mel, lsaA, vgaD, vgaALC, lsaC, salA, lsaB, msrE, vgaB, lsaE, msrC, oleB, mdtF, lmrD, lmrC, vgaA, tlrC, msrA, sav1866, bcrA, vgaE, macB, vanHD, carA, oleC | efflux pump conferring antibiotic resistance |
| 0.16216 | LRA-3 | leuO, LRA-3 | antibiotic inactivation enzyme; beta-lactam resistance gene |
| 3.23125 | OpmH | APH(9)-Ia, OpmH, APH(6)-Ib | efflux pump conferring antibiotic resistance; triclosan resistance gene |
| 2.68E-32 | cpxA | CMY-99, CMY-98, CMY-93, CMY-90, CMY-95, CMY-94, CMY-13, CMY-12, CMY-17, CMY-16, CMY-15, CMY-14, CMY-18, CMY-76, smeS, ACT-6, ACT-7, ACT-4, ACT-5, ACT-2, ACT-3, ACT-1, ACT-9, MIR-8, MIR-9, MIR-2, MIR-3, MIR-6, MIR-4, MIR-5, CMY-66, CMY-67, CMY-64, evgS, CMY-62, CMY-63, CMY-60, CMY-61, CMY-68, CMY-69, CMY-6, MIR-14, MIR-15, MIR-16, MIR-17, MIR-10, MIR-11, MIR-12, MIR-13, CMY-75, CMY-74, CMY-77, CMY-65, CMY-71, CMY-70, CMY-73, CMY-72, CMY-79, CMY-78, CMY-2, CMY-7, ACT-32, CMY-5, CMY-4, ACT-18, CMY-23, cpxA, ACT-13, ACT-36, CMY-41, CMY-42, CMY-43, CMY-44, CMY-45, CMY-46, ACT-31, CMY-48, CMY-49, baeS, CMY-47, PmrB, ACT-25, ACT-24, ACT-27, ACT-21, ACT-20, CMY-59, CMY-58, CMY-57, CMY-56, CMY-55, CMY-54, CMY-53, ACT-28, CMY-51, CMY-50, ACT-23, ACT-22, CMY-119, CMY-118, CMY-113, CMY-112, CMY-111, CMY-110, CMY-117, CMY-116, CMY-115, CMY-114, LAT-1, ACT-29, ACT-10, CMY-28, CMY-29, ACT-14, ACT-15, ACT-16, ACT-17, CMY-22, ACT-19, CMY-20, CMY-21, CMY-26, CMY-27, CMY-24, CMY-25, adeS, CMY-108, CMY-100, CMY-101, CMY-102, CMY-103, CMY-104, CMY-105, CMY-40, ACT-37, ACT-12, CMY-39, CMY-38, ACT-35, CMY-31, CMY-30, CMY-33, CMY-32, CMY-35, CMY-34, CMY-37, arlS, CFE-1, CMY-84, CMY-85, CMY-86, CMY-87, CMY-80, CMY-81, CMY-82, CMY-83, vanSG, vanSF, vanSE, vanSD, vanSC, vanSB, vanSA, vanSO, vanSN, vanSM, vanSL, vanSI | efflux pump conferring antibiotic resistance; aminocoumarin resistance gene; aminoglycoside resistance gene; gene modulating antibiotic efflux |
| 2.29E-11 | oleC | srmB, ANT(2'')-Ia, otrC, novA, msrC, Streptococcus agalactiae mprF, lsaC, mel, lsaB, msrE, lsaE, msrA, oleB, oleC, lmrD, lmrC, mphC, tlrC, sav1866, bcrA, carA, vanL | efflux pump conferring antibiotic resistance |
| 1.07E-15 | robA | robA, tetW | chloramphenicol resistance gene; gene modulating antibiotic efflux; fluoroquinolone resistance gene; efflux pump conferring antibiotic resistance; tetracycline resistance gene; rifampin resistance gene; beta-lactam resistance gene |
| 1.32E-13 | OpmH | oprJ, OpmH, mdtP, oprM, Streptomyces rishiriensis parY mutant conferring resistance to aminocoumarin, smeF, oprA, smeC, Bacillus subtilis mprF, lsaE, APH(2'')-Ig, mdtA, mdsC, tolC, APH(2'')-IIa, adeH | efflux pump conferring antibiotic resistance; triclosan resistance gene |
| 1.15E-08 | Bifidobacteria intrinsic ileS conferring resistance to mupirocin | acrB, FOX-10, Bifidobacteria intrinsic ileS conferring resistance to mupirocin, FOX-7, FOX-5, arnA, acrF, FOX-2, GOB-1 beta-lactamase | mupirocin resistance gene |
| 5.18E-29 | arlR | evgA, VEB-9, VEB-8, baeR, VEB-5, VEB-4, VEB-7, VEB-6, VEB-1, VEB-3, VEB-2, evgS, vanRA, vanRB, vanRC, vanRD, vanRE, vanRF, vanRG, vanRI, vanRL, smeR, vanRN, VEB-1b, mtrA, adeR, mexV, arlR, cpxR, vanTrL, kdpE, adeK, VEB-1a, vanRM, vanRO | efflux pump conferring antibiotic resistance; gene modulating antibiotic efflux; fluoroquinolone resistance gene |
| 2.36E-13 | vgaA | srmB, otrC, novA, mel, salA, lsaB, vgaALC, msrE, lsaA, vgaD, lsaC, vgaB, msrC, lsaE, oleB, oleC, lmrD, lmrC, vgaA, tlrC, msrA, sav1866, PER-2, PER-1, PER-7, PER-6, PER-5, PER-4, bcrA, vgaE, macB, carA | efflux pump conferring antibiotic resistance; streptogramin resistance gene; pleuromutilin resistance gene |
| 1.05E-85 | vanL | vanC, vanA, AAC(6')-Iw, vanB, vanE, vanD, vanG, vanF, vanI, vanM, vanL, vanO, vanN | glycopeptide resistance gene; antibiotic resistance gene cluster, cassette, or operon; gene conferring antibiotic resistance via molecular bypass |
| 2.19E-10 | mfd | mfd | antibiotic target protection protein; fluoroquinolone resistance gene |
| 4.03E-105 | aminocoumarin resistant cysB | aminocoumarin resistant cysB | aminocoumarin resistance gene |
| 3.57E-07 | tetD | SRT-2, SRT-1, DHA-3, DHA-2, DHA-1, DHA-7, DHA-6, DHA-5, DHA-22, DHA-20, DHA-21, gadE, tetK, mexB, tetD, adiY, DHA-19, DHA-18, DHA-13, DHA-12, DHA-10, DHA-17, DHA-16, DHA-15, DHA-14 | efflux pump conferring antibiotic resistance; tetracycline resistance gene |
| 2.13E-07 | NmcR | leuO, mexT, NmcR | antibiotic inactivation enzyme; beta-lactam resistance gene; gene modulating beta-lactam resistance |
| 1.88E-25 | tet36 | tetB(P), tetQ, tetS, tetT, otrA, tet32, tetM, tetW, tetO, tet36, tet44 | antibiotic target protection protein; tetracycline resistance gene |
| 1.31E-79 | qacA | emrY, tet45, tet42, tet43, tet41, norB, cmlB1, qacB, emrB, emrD, qacA, mdtH, mdfA, blt, emeA, fexA, facT, tet39, tet38, pmrA efflux pump, tet33, tet31, tet30, tetY, tetZ, floR, tetC, otrB, bmr, tetH, cmlA4, cmlA5, mdtG, tetL, mdtD, tetA, tcr3, tetB, tetE, lmrB, mdtM, mdtL, tetK, cmlA6, pp-flo, cmlA, cmlB, cmlA1, norA, qepA, tcmA, bcr-1, cmrA, tetJ | efflux pump conferring antibiotic resistance; fluoroquinolone resistance gene |
| 2.45E-107 | baeR | smeR, baeR | efflux pump conferring antibiotic resistance; aminocoumarin resistance gene; aminoglycoside resistance gene; gene modulating antibiotic efflux |
| 6.24E-06 | cpxR | Mrx, adeR, baeR, vanRM, vanRL, oprA, smeR, cpxR, vanRA, vanRB, vanRC, vanRD, vanRE, vanRF, vanRG, mtrA, vanRI, kdpE, PBP2x, vanRN, vanRO | efflux pump conferring antibiotic resistance; aminocoumarin resistance gene; aminoglycoside resistance gene; gene modulating antibiotic efflux |
| 1.22E-14 | emrA | vanYD, mdsA, emrK, acrE, emrA, mtrC, smeD, smeA, mdtN, mexV, cmeA, mexX, AIM-1, TriA, amrA, mexP, TriB, mdtA, mexM, mexJ, mdtE, mexE, lmrC, mexC, mexA, ceoA, adeF, acrA, macA | efflux pump conferring antibiotic resistance; fluoroquinolone resistance gene |
| 3.55E-19 | lsaA | srmB, otrC, novA, mel, lsaA, lsaB, vgaALC, msrE, salA, vgaD, lsaC, vgaB, lsaE, msrC, oleB, oleC, vgaA, lmrC, tlrC, msrA, sav1866, Chlamydia trachomatis murA, bcrA, vgaE, macB, carA | efflux pump conferring antibiotic resistance; lincosamide resistance gene; streptogramin resistance gene; pleuromutilin resistance gene |
| 1.15E-06 | LRA-8 | CAU-1 beta-lactamase, LRA-12, SFB-1, GIM-1, LRA-19, OXA-61, CGB-1 beta-lactamase, TUS-1 beta-lactamase, NDM-9, NDM-8, FEZ-1 beta-lactamase, NDM-5, NDM-4, NDM-7, NDM-6, NDM-1, NDM-3, NDM-2, LRA-17, BJP-1, NDM-12, IND-14, Rm3 beta-lactamase, MUS-2 beta-lactamase, SIM-1 beta-lactamase, THIN-B beta-lactamase, GOB-1 beta-lactamase, NDM-10, NDM-13, SPG-1, MSI-1, LRA-2, AIM-1, cphA4, IND-4, MUS-1 beta-lactamase, L1 beta-lactamase, PEDO-1, IND-2a, IND-11, IMP-40, SLB-1, IND-12, IND-15, LRA-3, SMB-1 beta-lactamase, KHM-1 beta-lactamase, IND-1, PEDO-3, IND-3, IND-2, IND-5, IMP-2, IND-7, IND-6, IND-9, IND-8, IMP-3, LRA-9, LRA-8, DIM-1, IND-10, JOHN-1 beta-lactamase, IMP-34, mexQ, EBR-1 beta-lactamase, BcII | antibiotic inactivation enzyme; beta-lactam resistance gene |
| 1.71E-07 | aminocoumarin resistant cysB | ErmO, mexT, cpxR, NmcR, aminocoumarin resistant cysB, mexI | aminocoumarin resistance gene |
| 2.31E-73 | aminocoumarin resistant alaS | aminocoumarin resistant alaS | aminocoumarin resistance gene |
| 3.63E-30 | vanHB | tetX, emrY, vanHB, norA, vanHA, vanHF, vanHD, vanHO, vanHM | glycopeptide resistance gene; antibiotic resistance gene cluster, cassette, or operon; gene conferring antibiotic resistance via molecular bypass |
| 1.92E-13 | NmcR | cfrA, leuO, NmcR, aminocoumarin resistant cysB, Streptomyces rishiriensis parY mutant conferring resistance to aminocoumarin | antibiotic inactivation enzyme; beta-lactam resistance gene; gene modulating beta-lactam resistance |
| 3.79E-07 | tet34 | OXA-363, OXA-362, tet34, mexI | gene conferring antibiotic resistance via molecular bypass |
| 1.76E-53 | mexG | mexG | efflux pump conferring antibiotic resistance; fluoroquinolone resistance gene |
| 8.35E-06 | lsaC | srmB, novA, CGB-1 beta-lactamase, mel, IND-12, IND-15, lsaA, lsaB, vgaALC, vgaE, salA, vgaD, lsaC, vgaB, lsaE, msrC, mecR1, oleB, IND-2a, IND-11, lmrD, lmrC, vgaA, tlrC, IND-2, IND-4, sav1866, IND-9, bcrA, macB, carA | efflux pump conferring antibiotic resistance; lincosamide resistance gene; streptogramin resistance gene; pleuromutilin resistance gene |
| 1.23E-30 | Streptomyces cinnamoneus EF-Tu mutants conferring resistance to elfamycin | tetB(P), tetQ, tetS, tet44, tetT, otrA, Streptomyces cinnamoneus EF-Tu mutants conferring resistance to elfamycin, tetM, tetW, tetO, mexG, tet36, gadW, tet32 | gene involved in self-resistance to antibiotic; antibiotic resistant gene variant or mutant; elfamycin resistance gene |
| 2.10E-30 | Streptomyces cinnamoneus EF-Tu mutants conferring resistance to elfamycin | Streptomyces cinnamoneus EF-Tu mutants conferring resistance to elfamycin, mexB, adeB | gene involved in self-resistance to antibiotic; antibiotic resistant gene variant or mutant; elfamycin resistance gene |
| 2.04E-12 | aminocoumarin resistant cysB | AAC(2')-Ia, leuO, mexT, Listeria monocytogenes mprF, NmcR, aminocoumarin resistant cysB | aminocoumarin resistance gene |
| 1.09E-22 | aminocoumarin resistant cysB | leuO, mexT, NmcR, aminocoumarin resistant cysB, sul2 | aminocoumarin resistance gene |
| 1.40E-13 | tcmA | emrY, mdtD, tet42, tet43, TEM-77, norB, cmlB1, cmlA6, qacB, emrB, emrD, qacA, TEM-73, norA, mdtH, mdfA, blt, emeA, fexA, mdtM, tet39, pmrA efflux pump, tet33, tet31, cmrA, tetZ, mepA, floR, tetC, otrB, bmr, cmlv, cmlA5, mdtG, tetL, cmlA1, tcr3, tetB, tetE, lmrB, facT, mdtL, tet41, TEM-146, TEM-145, pp-flo, cmlA, cmlB, cmlA4, qepA, tcmA, cmx, bcr-1, TEM-30, cmr | efflux pump conferring antibiotic resistance |
| 1.08E-23 | Mycobacterium tuberculosis gidB mutation conferring resistance to streptomycin | ceoB, spd, mdtF, cmeA, Mycobacterium tuberculosis gidB mutation conferring resistance to streptomycin | antibiotic target modifying enzyme; antibiotic resistant gene variant or mutant; aminoglycoside resistance gene |
| 6.18E-06 | novA | tetK, dfrA25, tet45, IMP-9, novA, PBP1a, mdtA, tetL, ErmS, IMP-45 | efflux pump conferring antibiotic resistance; aminocoumarin resistance gene |
| 7.82E-106 | kdpE | evgA, baeR, APH(3'')-Ia, GES-24, GES-20, GES-21, SHV-50, evgS, GES-15, vanTG, GES-16, vanRA, vanRB, vanRC, vanRD, vanRE, vanRF, vanRG, vanRI, abeM, vanRL, smeR, vanRN, GES-6, mtrA, GES-14, adeR, vanRM, GES-18, arlR, vanXM, OXA-9, cpxR, kdpE, GES-5, GES-4, vanRO, arr-1 | aminoglycoside resistance gene |
| 1.54E-31 | mexS | tetS, mexS, vanRE | efflux pump conferring antibiotic resistance; chloramphenicol resistance gene; trimethoprim resistance gene; gene modulating antibiotic efflux; fluoroquinolone resistance gene |
| 1.68E-05 | arnA | vanTG, mefE, rmtC, BlaB beta-lactamase, opmE, PEDO-3, arnA, JOHN-1 beta-lactamase | polymyxin resistance gene; gene altering cell wall charge conferring antibiotic resistance |
| 1.31E-47 | sav1866 | srmB, ANT(2'')-Ia, otrC, PBP1a, novA, mel, lsaA, vgaD, vgaALC, lsaC, salA, lsaB, msrE, vgaB, msrC, lsaE, oleB, oleC, lmrD, lmrC, vgaA, tlrC, msrA, sav1866, bcrA, vgaE, macB, carA | efflux pump conferring antibiotic resistance |
| 1.12E-10 | NmcR | OXA-208, OXA-92, OXA-69, OXA-390, OXA-250, OXA-380, PmrB, catII, OXA-248, cat, OXA-110, OXA-112, NmcR, OXA-116, OXA-371, OXA-107, OXA-120 | antibiotic inactivation enzyme; beta-lactam resistance gene; gene modulating beta-lactam resistance |
| 3.99E-51 | macB | srmB, otrC, novA, mel, lmrD, lsaA, lsaB, vgaALC, lsaC, oprN, salA, vgaD, msrE, vgaB, msrC, lsaE, oleB, oleC, vgaA, lmrC, tlrC, msrA, sav1866, Listeria monocytogenes mprF, bcrA, vgaE, macB, carA | efflux pump conferring antibiotic resistance; macrolide resistance gene |
| 2.13E-24 | evgA | evgA, mefA, baeR, OXA-48, OXA-244, OXA-245, OXA-247, OXA-199, OXA-204, vanRA, vanRB, vanRC, vanRD, vanRE, vanRF, vanRG, vanRI, vanRM, vanRN, vanRO, OXA-54, mtrA, adeR, gadE, arlR, cpxR, mdtA, adeG, OXA-232, mexF, kdpE, sdiA, OXA-370, OXA-163, OXA-162, vanI, OXA-181 | gene modulating antibiotic efflux; macrolide resistance gene; fluoroquinolone resistance gene; efflux pump conferring antibiotic resistance; tetracycline resistance gene; beta-lactam resistance gene |
| 7.46E-50 | macB | srmB, otrC, novA, dfrA13, mel, lmrD, lsaA, abeM, lsaB, dfrA21, vgaALC, lsaC, salA, vgaD, msrE, vgaB, msrC, lsaE, oleB, oleC, vgaA, lmrC, tlrC, msrA, sav1866, bcrA, vgaE, macB, vanG, carA | efflux pump conferring antibiotic resistance; macrolide resistance gene |
| 1.29E-46 | vanTG | AAC(3)-Ib/AAC(6')-Ib'', MSI-1, vanTE, vanT, vanTG, vanTN, APH(6)-Id, vanTrL, vanTC | glycopeptide resistance gene; antibiotic resistance gene cluster, cassette, or operon; gene conferring antibiotic resistance via molecular bypass |
| 1.23E-34 | arnA | arnA | polymyxin resistance gene; gene altering cell wall charge conferring antibiotic resistance |
| 1.42E-22 | lmrC | srmB, otrC, vgaALC, novA, OXA-16, OXA-454, OXA-246, OXA-240, msrC, EreB, OXA-35, mel, AAC(6')-IIa, AAC(6')-IIc, AAC(6')-IIb, lsaB, OXA-56, OXA-13, OXA-19, OXA-74, AAC(6')-32, AAC(6')-31, tlrC, vgaD, salA, OXA-10, lsaC, vgaB, lsaE, lsaA, OXA-17, OXA-101, OXA-129, OXA-5, OXA-233, OXA-7, OXA-14, lmrD, lmrC, vgaA, OXA-256, msrA, carA, OXA-251, sav1866, OXA-147, AAC(6')-IId, msrE, OXA-142, vanRM, bcrA, vgaE, macB, OXA-183, oleB, OXA-28, OXA-11, oleC | efflux pump conferring antibiotic resistance; lincosamide resistance gene |

**ARO: Antibiotic Resistance Ontology, Model_type used is** protein homolog model or protein variant model
