# Supplementary material for: Genome sequencing and analysis of the first spontaneous Nanosilver resistant bacterium Proteus mirabilis strain SCDR1
Source: Antimicrob Resist Infect Control. 2017 Nov 23;6:119. doi: 10.1186/s13756-017-0277-x (PMC5701452; doi:10.1186/s13756-017-0277-x)
Supplement: Supplementary file 7 — Drug Resistance related protiens and its corresponding genes or proteins GenBank access numbers. (DOCX 16 kb) [file 13756_2017_277_MOESM7_ESM.docx]

| Table S7: Drug Resistance related protiens and its corresponding genes or proteins GenBank access numbers. | |
| --- | --- |
| **GenBank Reference sequence/Accession Number** | **Corresponding Protein** |
| AY061647.1 | Proteus mirabilis regulatory protein AcrR, membrane fusion protein AcrA, and inner membrane RND family protein AcrB genes, complete cds. (AcrAB multidrug efflux pump). |
| WP_004245070.1 | DNA-binding transcriptional repressor AcrR |
| WP_020945213.1 | Inner membrane rnd family protein acrb |
| CP004022.1 | baeR: is a response regulator that promotes the expression of MdtABC and AcrD efflux complexes. |
| CP004022.1 | baeS: is a sensor kinase in the BaeSR regulatory system. While it phosphorylates BaeR to increase its activity, BaeS is not necessary for overexpressed BaeR to confer resistance. |
| KM006423.1 | Proteus mirabilis strain N2 OprD-like porin (impR) gene, complete cds. |
| M11587.1 | Proteus mirabilis PM13 cat gene |
| WP_020945422.1 | type A chloramphenicol O-acetyltransferase (cata4) |
| GQ457568.1 | Proteus mirabilis strain PmrIp UDP-glucuronic acid decarboxylase gene, complete cds |
| WP_020945551.1 | bifunctional UDP-glucuronic acid oxidase/UDP-4-amino-4-deoxy-L-arabinose formyltransferase |
| AF038993.1 | Proteus mirabilis repressor protein (tetRJ) and tetracycline resistance protein (tetAJ) genes, complete cds |
| WP_020946223.1 | TetH/TetJ family tetracycline resistance MFS efflux pump |
| WP_012367924.1 | MFS family transporter (drug resistance transporter, EmrB/QacA subfamily) |
| CP004022.1 | EmrR (emrA): EmrAB-TolC overexpression |
| CP004022.1 | Multidrug resistance protein ErmA |
| CP004022.1 | Multidrug resistance protein ErmB |
| CP004022.1 | Membrane fusion component of tripartite multidrug resistance system |
| WP_020945848.1 | Multidrug transporter subunit MdtA |
| WP_020945849.1 | Multidrug transporter subunit MdtB |
| WP_020945850.1 | Multidrug transporter subunit MdtC |
| WP_012367928.1 | Multidrug efflux system protein MdtL |
| WP_020945656.1 | Multidrug transporter subunit MdtL |
| WP_004242957.1 | Multidrug transporter subunit MdtN |
| WP_020945578.1 | Multidrug resistance protein MdtH |
| WP_004242769.1 | Multidrug transporter subunit MdtJ |
| WP_004247986.1 | Multidrug transporter subunit MdtI |
| WP_004248167.1 | Multidrug resistance protein MdtK |
| WP_020946480.1 | Multidrug efflux protein (Multiple Antibiotic Resistance) |
| WP_020945124.1 | Multidrug efflux protein (AcrA) |
| WP_017827763.1, WP_020945212.1 | Multidrug ABC transporter permease/ATP-binding protein (Defense mechanisms) |
| WP_020945214.1 | MexE family multidrug efflux RND transporter periplasmic adaptor subunit (Drug Resistance) |
| WP_004249194.1 | MexH family multidrug efflux RND transporter periplasmic adaptor subunit |
| WP_020945223.1 | MATE family efflux transporter, Na+-driven multidrug efflux pump (multidrug and toxic compound extrusion and Defense mechanisms) |
| WP_004252255.1, WP_020946480.1 | Multidrug export protein EmrA |
| WP_020945660.1 | Channel-forming component of a multidrug resistance efflux pump |
| WP_004245854.1, WP_017628500.1 | Acriflavine resistance protein B (ethidium bromide and acriflavine Resistance, Defense mechanisms) |
| WP_004243278.1 | Methyl viologen resistance protein (ethidium resistance protein) |
| CP004022.1 | Ethidium bromide-methyl viologen resistance protein EmrE |
| WP_004243958.1 | NADPH quinone reductase MdaB |
| WP_004245856.1, WP_020946318.1 | QacE family quaternary ammonium compound efflux SMR transporter (Defense mechanisms, Ethidium bromide Resistance protein) !!!! |
| WP_004246423.1 | Multidrug resistance protein D |
| WP_004245413.1 | Quaternary ammonium compound-resistance protein SugE |
| WP_020945304.1, WP_004247640.1  WP_012367562.1 | Metallo-beta-lactamase superfamily protein |
| WP_012368746.1, WP_004246179.1 | beta-lactam binding protein AmpH |
| WP_020945124.1 | Multidrug efflux pump (AcrA) |
| WP_020945215.1 | Potassium efflux protein KefA |
| WP_023843549.1 | bicyclomycin/multidrug efflux system |
| AGS58943.1 | type I secretion outer membrane protein, TolC precursor |
| AGS58943.1 | Type_I_sec_TolC |
| AGS58943.1 | OEP |
| NC_002695 | Histone-like protein (hns) |
| WP_020945915.1 | DNA gyrase subunit A (gyrA) |
| CP004022.1 | Topoisomerase IV subunit A |
| CP004022.1 | Topoisomerase IV subunit B |
| CP004022.1 | Dihydropteroate synthase sulfonamide antibiotics resistant folP |
| CP004022.1 | SoxR: up regulate many multidrug efflux pumps |
| CP004022.1 | Translation elongation factor (Tu) |
| CP004022.1 | cpxA: Copper sensory histidine kinase |
| CP004022.1 | cpxR: Copper-sensing two-component system response regulator CpxR |
| CP004022.1 | Macrolide-specific efflux protein MacA |
| CP004022.1 | Macrolide export ATP-binding/permease protein MacB |
| CP004022.1 | phoP: a direct repressor of the macAB efflux genes. |
| CP004022.1 | phoQ: Sensor histidine kinase PhoQ |
| CP004022.1 | Outer membrane porin OmpC |
